# Rigid and planar π-conjugated molecules leading to long-lived intramolecular charge-transfer states exhibiting thermally activated delayed fluorescence

**DOI:** 10.1038/s41467-024-53740-1

**Published:** 2024-11-07

**Authors:** Suman Kuila, Hector Miranda-Salinas, Julien Eng, Chunyong Li, Martin R. Bryce, Thomas J. Penfold, Andrew P. Monkman

**Affiliations:** 1 Department of Physics, Durham University, South Road, Durham, DH1 3LE UK; 2 Department of Chemistry, Durham University, South Road, Durham, DH1 3LE UK; 3 Chemistry, School of Natural and Environmental Sciences, Newcastle University, Newcastle upon Tyne, NE1 7RU UK; 4 Present Address: Renewable and Sustainable Energy Institute, University of Colorado Boulder, Boulder, Colorado 80309 US

**Keywords:** Optical materials, Organic LEDs

## Abstract

Intramolecular charge transfer (ICT) occurs when photoexcitation causes electron transfer from an electron donor to an electron acceptor within the same molecule and is usually stabilized by decoupling of the donor and acceptor through an orthogonal twist between them. Thermally activated delayed fluorescence (TADF) exploits such twisted ICT states to harvest triplet excitons in OLEDs. However, the highly twisted conformation of TADF molecules results in limited device lifetimes. Rigid molecules offer increased stability, yet their typical planarity and π-conjugated structures impedes ICT. Herein, we achieve dispersion-free triplet harvesting using fused indolocarbazole-phthalimide molecules that have remarkably stable co-planar ICT states, yielding blue/green-TADF with good photoluminescence quantum yield and small singlet-triplet energy gap < 50 meV. ICT formation is dictated by the bonding connectivity and excited-state conjugation breaking between the donor and acceptor fragments, that stabilises the planar ICT excited state, revealing a new criterion for designing efficient TADF materials.

## Abstract (web summary)

The authors report thermally activated delayed fluorescence in rigid planar donor-acceptor molecules achieving decoupling of ground and excited states through a bonding/antibonding connectivity between donor and acceptor without alarge dihedral twist angle between them.

## Introduction

Significant progress has been made in the field of luminescent materials having efficient ambient triplet harvesting for applications in organic light-emitting diodes (OLEDs), bio-imaging and photo-catalysis^1–3^. In this regard, triplet harvesting via thermally activated delayed fluorescence (TADF) has emerged as a challenger technology to the incumbent room-temperature phosphorescence in OLEDs^1,4–6^. The efficiency of TADF relies in part on minimizing the energy gap between the lowest excited singlet and triplet states. This is usually achieved using donor-acceptor charge-transfer chromophores, where orthogonally oriented D and A units, i.e. twisted D-A molecules^1,6–9^, minimises the electron exchange energy and therefore the splitting between the lowest excited singlet and triplet states, provided they exhibit the same spatial symmetry^10^. Different strategies have also been used to realize this objective, including spiro-linked D and A^11,12^, arranging D and A in spatial proximity to achieve through-space charge transfer^13,14^, or forming molecular exciplexes^15,16^. One of the primary concerns with twisted D-A molecules is stability during device operation, as TADF OLED lifetimes are as yet very limited^17^. To achieve high stability in an OLED, it is believed that all rotationally flexible tertiary N-C bonds must be removed from all molecules including TADF molecules as these are the weakest chemical bonds present in their structure and therefore prone to degradation^18,19^. It is hoped that spiro-type TADF emitters^20^, especially used in hyperfluorescence schemes^21–23^, could overcome this, as recently demonstrated by Stavrou et al.^23^, but as yet they also have not demonstrated great lifetime improvement. Fully rigid structures should result in photochemically stable molecules, offering short CT lifetimes with fast fluorescence decay rates, high PLQY, and fast delayed fluorescence with negligible energy dispersion^24–26^. The latter is very large in the flexible, twisted D-A molecules, because of distributions of the (dihedral) twist angles between D and A sub units, which result in very broad emission bands and long-lived TADF components, detrimental to OLED operational lifetime because of enhanced exciton-charge quenching possibilities^27^. However, the main obstacle to exploiting a fully rigid D-A molecular structure is the inherent planarity and conjugation which is believed to make small exchange energy intramolecular charge transfer (ICT) improbable^7–10^. There has been a significant amount of work developing the multi-resonance (MR-TADF) approach where short-range charge transfer^28–31^ between two adjacent atoms plays a dominant role over long-range charge-transfer^32–35^ within the molecules in their excited states. However, while this has achieved exciting results, it represents an entirely new framework^30^ with a limited chemical space due to molecular design restrictions required to achieve MR-TADF. MR-TADF materials with small ∆E_ST_ and fast rISC rates are emerging^35–37^, but the majority of these emitters are still central in hyperfluorescence OLEDs as the end-acceptors, i.e., terminal emitter, owing to their exceptional colour-purity, and typically twisted D-A TADF emitters act as FRET (Förster Resonance Energy Transfer) sensitizers^17,22,23^.Figure panels ‘e’ and ‘f’ are mentioned in Figure 2 legend but are not present in the figure. Please indicate its position in the figure.

With the objective of achieving desirable rigidity for low CT dispersion energies and high fluorescence rates, our research explored an unconventional pathway of utilising rigidly planar D-A molecules that give unexpected ambient triplet harvesting based on a long-range intramolecular charge-transfer state, distinctly different from the MR-TADF and twisted D-A ICT molecules. Previously, a few rigidified aminobenzonitriles have shown ICT states without the need of a large-amplitude rotational motion required to form a twisted conformation^38^. However, the stability of these co-planar ICT states depend on the energy gap and coupling between the low-lying singlet states, and ICT emission is observed only in highly polar solvents, albeit no triplet contribution has ever been reported^39–41^. To the best of our knowledge, a fully-conjugated, co-planar and rigidified donor-acceptor chromophore giving strong ICT emission involving both singlet (prompt fluorescence) and triplet excited state (TADF) in non-polar environments has not been observed. Rajamalli et al. proposed a D-A-D system showing TADF which they claimed was planarized through hydrogen bonding between D and A^42^. Subsequently, Chen et al.^43^ and He et al.^44^ invoked similar D-A hydrogen bonding schemes in HMAT-TRZ and ICZ-TRZ, respectively for potential TADF design strategy. However, both these derivatives show large ∆E_ST_ ( > 0.43 eV) and do not show any TADF in dilute films, hinting towards triplet-triplet annihilation (TTA)^43,45^ or weak exciplex derived^44^ TADF when doped in appropriate hosts at high doping concentrations. Notably, detailed potential energy surface analysis combined with experimental time-resolved spectroscopy studies on several model intramolecular H-bond containing D-A molecules by Hempe et al.^46^ and Bergmann et al.^47^ suggested that such D-A hydrogen bonds are unlikely to form and drive the rISC process.

**Fig. 1:**
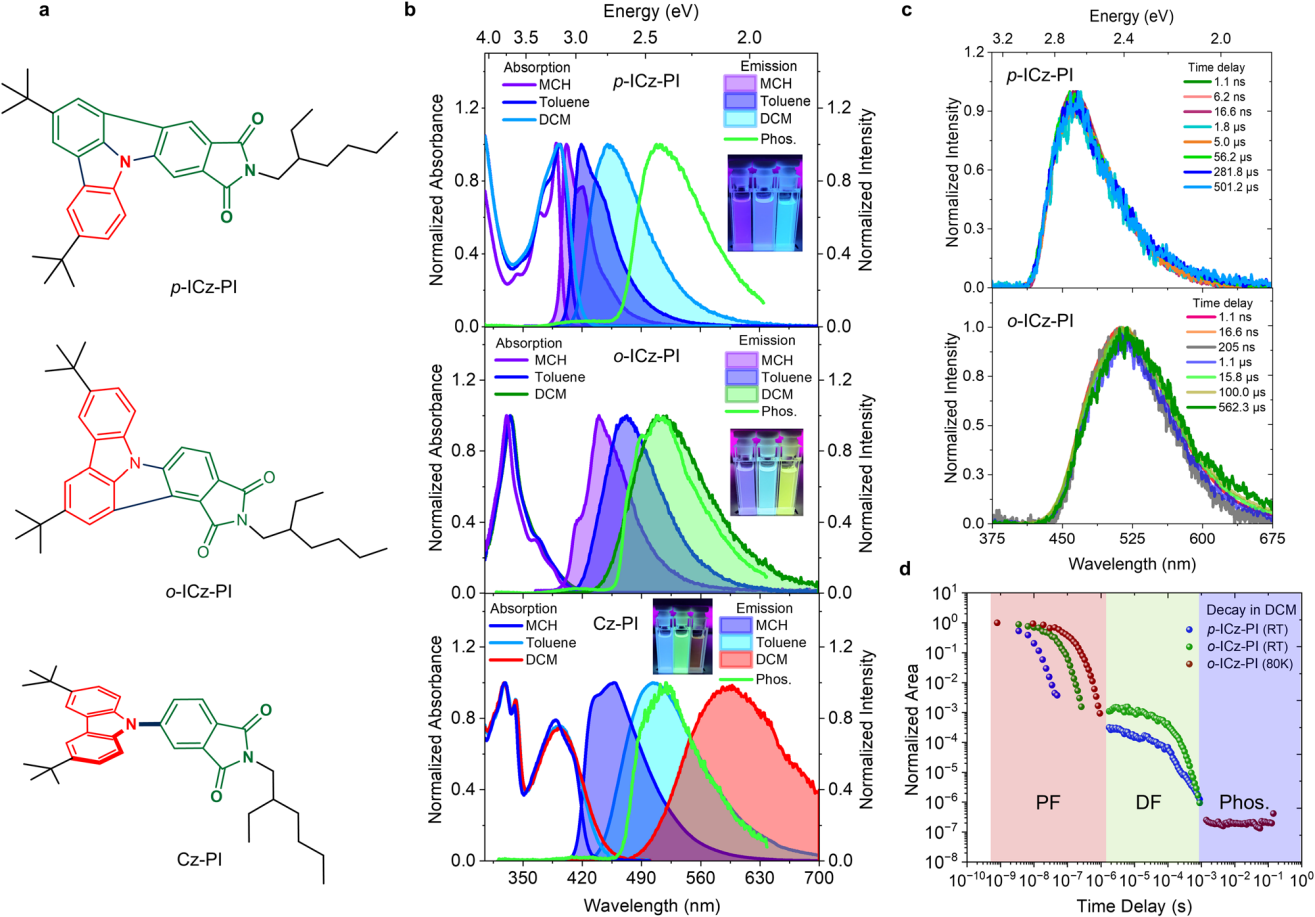
Absorption, fluorescence, delayed fluorescence, and phosphorescence of the investigated molecules. **a** Molecular structures of the three charge-transfer emitters (***p*-ICz-PI,** ***o*-ICz-PI**, and **Cz-PI**) studied in this work. The donor and acceptor units are shown in red and green colors, respectively. **b** Normalized absorption and steady-state emission spectra of dilute ***p*-ICz-PI** (top panel), ***o*-ICz-PI** (middle panel) and **Cz-PI** (bottom panel) solutions in methylcyclohexane, toluene and dichloromethane ([c] = 2 × 10^−5^ M). Inset shows the change in emission color upon changing the solvent polarity when excited at 365 nm by a UV-lamp. Phosphorescence (measured using time-gated emission at 80 K with a high time-delay of 80 ms) in 1 wt.% zeonex shown here to highlight the locally-excited nature of the T_1_ state in all three molecules. **c** Time-resolved emission spectra and (**d**) time-resolved decay of ***p*-ICz-PI** (room temperature) and ***o*-ICz-PI** (room temperature and 80 K) in degassed dichloromethane. λ_exc_ = 355 nm, [c] = 2 × 10^−5^ M. Three different regions of prompt fluorescence (PF), delayed fluorescence (DF) and phosphorescence (Phos.) are shown in Fig. 1d, with the DF completely quenched at 80 K.

To approach this seemingly intractable problem, we have developed a new molecular design utilizing a fused carbazole (indolocarbazole, ICz)^48^ in combination with a phthalimide^49^ (PI) acceptor unit, in fully planar rigid molecular structures (ICz-PI; Fig. 1) giving efficient TADF. In the context of triplet harvesting, phthalimides are particularly useful as they give efficient π-π * and n-π* intersystem crossing^43^. In addition, they are relatively weak donors and acceptors with significantly high triplet energies, ideal for blue-emitting charge-transfer systems^24,49^. Furthermore, two isomers of ICz-PI which differ in the position of N-C and C-C bonds connecting the donor-acceptor fragments, exhibit very different excited state behaviour and photophysics. Most importantly, these emitters show stable ICT states and triplet harvesting properties both in solution and doped solid films with good photoluminescence quantum yields. We propose a mechanism that explains how conjugation is broken in the excited state of these ICz-PI molecules, such that the planar ICT states are stabilised to give long CT prompt lifetimes of >30 ns and persistent TADF. Our observations break new ground in showing that it is possible; (i) to stabilise ICT states in a fully-planar conjugated rigidified molecules and (ii) to achieve TADF without orthogonal D-A or highly spatially separated D and A units.

## Results and Discussion

### Molecular design and synthesis

***o-*ICz-PI** and ***p*-ICz-PI** (Fig. 1a) were synthesized in one-pot via a synchronous palladium-catalyzed Buchwald-Hartwig and Heck-coupling reaction pathway from previously reported bromine substituted di-*tert*-butylcarbazole and phthalimide precursors. **Cz-PI** (Fig. 1a) was synthesized following similar conditions using unsubstituted di-*tert*-butylcarbazole via Buchwald-Hartwig coupling. 2-Ethylhexyl chains were attached to ensure good solubility of the molecules. Detailed synthetic procedures are given in the Supporting Information. All the molecules synthesized in this work were characterized by ^1^H, ^13^C NMR spectroscopy and high-resolution mass spectrometry (HRMS).

### Optical properties

The optical properties of both isomers, ***p*-ICz-PI** and ***o*-ICz-PI** were measured in three solvents of varying polarity: methylcyclohexane (MCH), toluene, and dichloromethane (DCM), (Fig. 1). ***p*-ICz-PI** has a distinct vibronically structured absorption band between 360–420 nm in all solvents and a molar extinction coefficient of 37925 Lmol^−1^cm^−1^ in DCM at 400 nm (Fig. 1 and Supplementary Fig. 1). In contrast, the absorption of ***o*-ICz-PI** in this region is significantly weaker (7320 Lmol^−1^cm^−1^) and the first peak in the absorption band is shifted to ~330 nm (Fig. 1b and Supplementary Fig. 1). This signifies a fundamentally different ground state electronic character between the two isomers, a trend observed in different solvents (Fig. 1b and Supplementary Fig. 1a). The ***p*-ICz-PI** absorption band redshifts by only 20 nm in DCM, indicating a weak charge-transfer contribution to this otherwise strongly local transition. The reduced intensity of these transitions in ***o*-ICz-PI** implies stronger CT character, confirmed by the electronic density difference plots for each molecule shown in the Supplementary Information (Section 5) and the trend in oscillator strength (*f*) related to ground state absorption (Supplementary Tables 4–13) which also decreases consistent with a decreased overlap between the initial and final states. For comparison, **Cz-PI**, (Fig. 1), the twisted D-A analogue of ICz-PI^50^ has a strong absorption at 320 and 340 nm as seen in ***o*-ICz-PI**, which is attributed to the carbazole/indolocarbazole unit, and a second band at 400 nm with a longer red absorption tail (> 425 nm) than ***p*-ICz-PI**, characteristic of a typical twisted D-A direct charge-transfer absorption (Fig. 1b and Supplementary Fig. 1a). This confirms that in ***p*-ICz-PI**, the 360–420 nm band is predominantly a local π-π* transition with a weak CT character, whereas in ***o*-ICz-PI** the CT character is far stronger^51^. The absorption of unsubstituted PI occurs at much higher energy, 3.81 eV (325 nm)^52^.

Upon excitation at 355 nm in MCH, ***p*-ICz-PI** exhibits a mirror-image emission band with small Stokes shift (11 nm), characteristic of a strongly local π-π* transition. Increasing the solvent polarity induces spectral broadening, larger Stokes shifts (28 nm in toluene and 56 nm in dichloromethane) and loss of vibronic structure, indicating increasing charge-transfer character. However, the excited state lifetime of ***p*-ICz-PI** does not change substantially, suggesting mixed LE/CT excited state character is retained even in DCM (τ_avg._ = 5.6 ns) and acetonitrile (τ_avg._ = 8.9 ns) as compared to MCH (τ_avg._ = 6.1 ns), see Supplementary Fig. 2a, Supplementary Table 1.

In comparison, ***o*-ICz-PI** has a broader featureless emission spectrum in polar solvents and large solvatochromic shifts (Fig. 1b). The Stokes shift in MCH (49 nm), toluene (81 nm) and DCM (123 nm) is substantially larger than ***p*-ICz-PI**, consistent with strong charge-transfer character. Fluorescence lifetimes confirm this with average lifetimes for ***o*-ICz-PI** increasing substantially from MCH (τ_avg._ = 19.04 ns) to DCM (τ_avg._ = 35.7 ns) (Supplementary Fig. 2b, Supplementary Table 1). Excitation of **Cz-PI** at 355 nm gives similar spectra to ***o***-**ICz-PI** but with significantly larger Stokes shifts (25 nm, 95 nm and 190 nm in MCH, toluene and DCM, respectively) indicative of pure CT character of a twisted DA-TADF molecule. The longer emission wavelength observed for more polar solvents also significantly increases non-radiative decay, as expected from the energy-gap law, indicated by the marked decrease in the excited state lifetime in high polarity solvents (Supplementary Fig. 2c, Supplementary Table 1).

The respective CT and LE character of the emitting states is further highlighted by the photoluminescence quantum yields (PLQY), which are substantially lower in DCM, for ***o*-ICz-PI**, Φ = 0.24 (0.37) ± 0.05, compared to ***p*-ICz-PI**, Φ = 0.41 (0.47) ± 0.05 (figures in brackets are degassed PLQY values, Supplementary Table 2) but show a marked oxygen dependence. The greater charge-transfer character in ***o*-ICz-PI** as compared to ***p*-ICz-PI** gives rise to a relatively slow rate of fluorescence (k_f_ = 2.8 × 10^7^ s^−1^) as compared to ***p*-ICz-PI** (k_f_ =17.9 × 10^7^ s^−1^) (Supplementary Table 2). In addition, both the emitters show similar k_rISC_ (3.05 × 10^4^ s^−1^ in ***o*-ICz-PI** and 3.21 × 10^4^ s^−1^ in ***p*-ICz-PI**) while ***p*-ICz-PI** has substantially faster intersystem crossing rate, 9.5 × 10^7^ vs 1.7 × 10^7^ s^−1^ for ***o*-ICz-PI**, i.e. one order of magnitude faster in rate. This explains a better triplet harvesting for ***o*-ICz-PI** with larger DF:PF ratio (1.52 for ***o*-ICz-PI** vs. 1.14 in ***p*-ICz-PI**). In DCM, **Cz-PI**, has a poor PLQY in air, Φ ~0.04; however, it increases substantially to Φ = 0.39 (0.61) ± 0.05, when measured in toluene. While the increase upon degassing is often attributed to the prevention of triplet state quenching, we note that singlet state quenching by oxygen also occurs when lifetimes are long^53^.

Finally, the two ICz-PI isomers also have different phosphorescence spectra, Fig. 1b (green trace). ***p*-ICz-PI** is rather structureless with onset at 2.64 eV (469 nm) whereas ***o*-ICz-PI** and **Cz-PI** are similar, having vibronically structured phosphorescence, onset at 2.70 eV (461 nm). The vibronic structure indicates that the lowest triplet state has LE character, effectively residing on the carbazole moiety^24^. For ***p*-ICz-PI** the lowest excited triplet state appears to be more delocalised in character.

### Triplet harvesting in degassed solution state

Time-resolved emission measurements of the two ICz-PI isomers were recorded in degassed DCM. ***p*-ICz-PI** emits the same prompt (1 ns − 40 ns) and a weak delayed fluorescence (2 µs − 1 ms) (Fig. 1c–d and Supplementary Fig. 3a). In contrast, ***o*-ICz-PI** showed a more pronounced enhancement in emission intensity upon degassing (Supplementary Fig. 3b, Supplementary Table 2), indicating a larger contribution from up-conversion of triplet states in line with a smaller ∆E_ST_ of 47 meV than ***p*-ICz-PI**, 290 meV (Fig. 1b). The ***o*-ICz-PI** emission has a long-lived prompt contribution (1 ns − 250 ns) and delayed emission (1 µs −1 ms) component (Fig. 1c, d) similar to **Cz-PI** (Supplementary Fig. 4). Notably, the delayed fluorescence of ***o*-ICz-PI** is completely quenched, and only strong very long lived phosphorescence is observed at long time-delays (>5 ms), when time-resolved spectra is measured at 80 K, in frozen DCM (Fig. 1d and Supplementary Fig. 3c). This observation confirms the thermally activated nature of the delayed fluorescence at room temperature and very low vibronic mediated internal conversion at 80 K. Furthermore, time-resolved emission in MCH shows clear vibronic features for ***p*-ICz-PI**, and a shorter lifetime compared to both ***o*-ICz-PI** and **Cz-PI** (Supplementary Fig. 5). We did not observe any delayed fluorescence in MCH, presumably due to a larger ∆E_ST_ in all cases.

### Ambient triplet harvesting in solid doped films

**Fig. 2:**
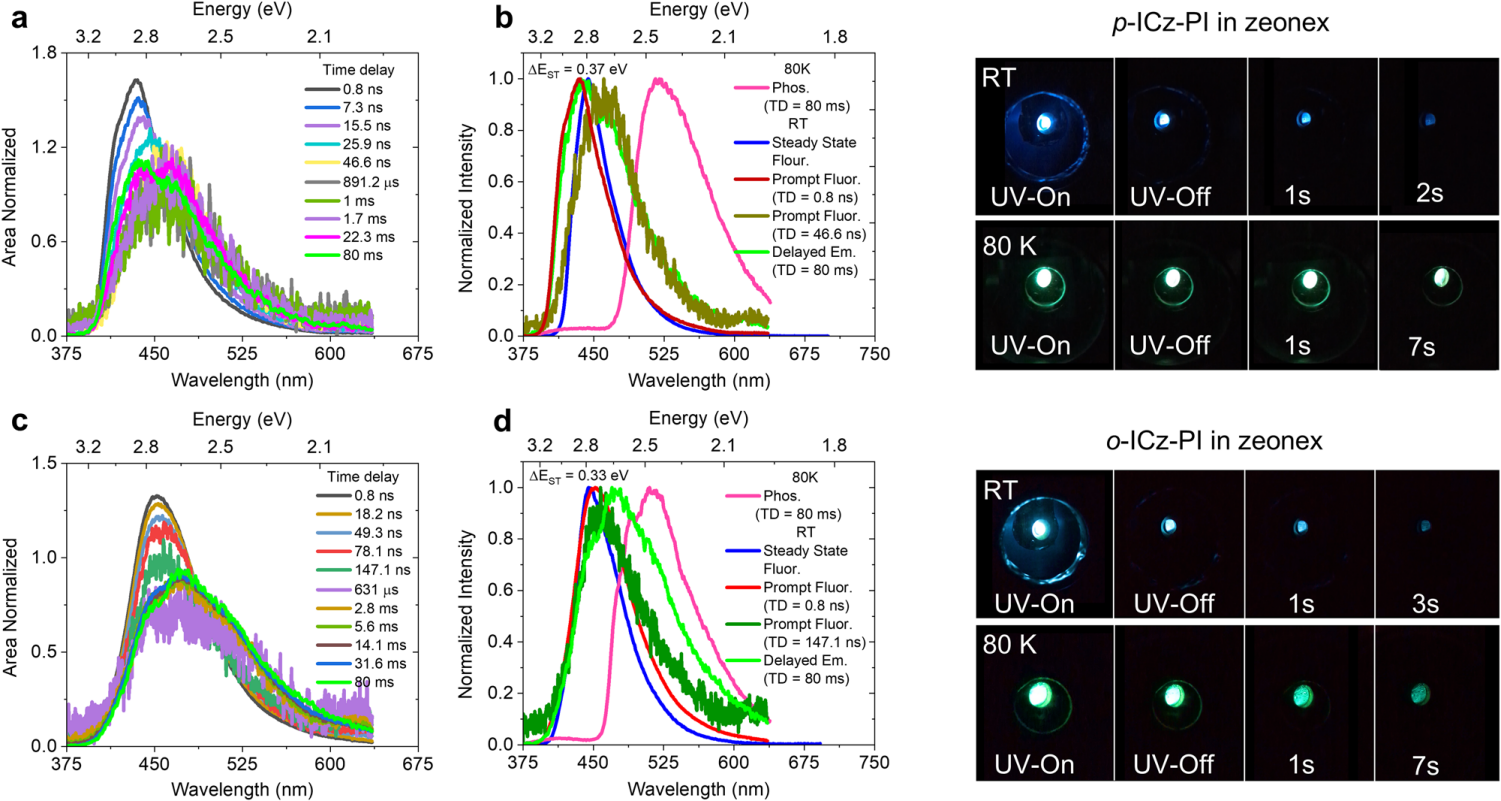
Triplet harvesting in solid state. (**a**) Time-resolved emission spectra, (**b**) steady-state photoluminescence (room temperature) and phosphorescence (80 K, 80 ms delay) of ***p*-ICz-PI** doped in 1 wt.% zeonex films. (**c**) Time-resolved emission spectra, (**d**) steady-state photoluminescence (room temperature), and phosphorescence (80 K, 80 ms delay) of ***o*-ICz-PI** doped in 1 wt.% zeonex films (λ_exc_ = 355 nm). Photographs of ***p*-ICz-PI** upper right panel and ***o*-ICz-PI** lower right panel doped in zeonex (1 wt.%), indicating a clear persistent emission behavior both at RT and 80 K upon excitation with a 355 nm UV source.

Figure 2a shows the time-resolved emission of 1 wt.% ***p*-ICz-PI** in zeonex films. ***p*-ICz-PI** exhibit a clear prompt emission with an onset at 401 nm (λ_max._ = 434 nm). This band red-shifts with time, which is attributed to the presence of aggregates at 1 wt.% doping, confirmed by their absence in 0.1 wt.% films (Supplementary Fig. 6). At long time-delays, > 800 µs, strong blue long-persistent emission, distinct from the phosphorescence (Fig. 2b, c), is observed when the laser is turned-off. This slow DF is consistent with the relatively high ∆E_ST_ = 0.37 eV in zeonex (Fig. 2b and Supplementary Fig. 7)^26^. 0.1 wt.% doped films (Supplementary Figs. 6a, b), also show this delayed fluorescence confirming its monomolecular origin.

In contrast, 1 wt.% ***o*-ICz-PI** in zeonex film shows distinctively different emission features (Fig. 2d). Prompt fluorescence (λ_onset_ = 411 nm and λ_max_ = 451 nm) lasts until 150 ns with no aggregate contribution. Delayed emission is seen at the same wavelengths as prompt fluorescence at time-delays > 630 µs. In addition, a new red-shifted band appears at around 516 nm. Similar emission features are also observed in 0.1 wt.% and 0.02 wt.% in zeonex and neat films (Supplementary Fig. 8). Steady-state fluorescence onset in these films is independent of doping ratio, unlike the ***p*-ICz-PI** isomer (Supplementary Fig. 8a). At 80 K, and long time-delays we observe only well-structured phosphorescence onset at 461 nm, (Fig. 2e and Supplementary Fig. 9). These observations suggest there is additional low levels of charge-transfer excimer (CT-excimer) formation, e.g. the D unit of one molecule interacting with the A unit of another, in ***o*-ICz-PI** doped zeonex films. We believe this propensity to form the CT-excimer state is because ***o*-ICz-PI** may well phase segregate as the zeonex films dry.

The aggregation effects on luminescence efficiency in 1 wt.% zeonex films are apparent compared to DCM solutions; ***p*-ICz-PI** which shows the largest aggregation, has a significant drop in PLQY to 0.23 ± 0.05, ***o*-ICz-PI** showing CT-excimer states suffers less PLQY quenching, Φ = 0.17 ± 0.05, whereas **Cz-PI** shows a near 100-fold increase in PLQY to Φ = 0.43 ± 0.05 in zeonex film (Supplementary Table 2). This suggests that the aggregate states of the ICz-PI isomers are different species. In ***p*-ICz-PI**, the aggregate not only red shifts the entire emission band but quenches PLQY by nearly a half, whereas in ***o*-ICz-PI**, the aggregate is much less pronounced and appears as a separate red emission band, i.e., a small additional CT-excimer contribution, that only moderately reduces PLQY. The ***o*-ICz-PI** CT-excimer state must be emissive, indicative of stronger donor and acceptor fragments in ***o*-ICz-PI** which can form CT-excimers, whereas in ***p*-ICz-PI** they are much weaker, precluding CT-excimer formation. This gives key insight into the differences in electronic structure between the two isomers. **Cz-PI** clearly behaves like a typical twisted D-A TADF molecule showing much higher PLQY in non-polar zeonex films, than in highly polar solvent (Supplementary Fig. 10 and Supplementary Table 2). No CT-excimer emission is observed for ***o*-ICz-PI** at 80 K in the 1 wt. % zeonex films which is fully in line with the thermally activated nature of CT-excimer formation (Supplementary Fig. 9). Notably, a clear dispersion in the CT emission maxima is observed in **Cz-PI**, whereas, the ICz-PI isomers are completely dispersion-free as evident from their time-resolved emission characteristics, owing to their rigidified co-planar structures (Supplementary Fig. 11).

The photophysics of the ICz-PI isomers (***p*-ICz-PI** and ***o*-ICz-PI)**, and **Cz-PI** were further studied in a small-molecule rigid host, mCP [1,3-bis(*N*-carbazolyl)benzene], having high triplet energy (T_1_ = 2.91 eV)^29^. Supplementary Fig. 12a shows the time-resolved emission profile of 10 wt.% doped films of ***p*-ICz-PI** in mCP at room temperature. A gradual red-shift is observed upon increasing time-delay (λ_onset_ = 421 nm, λ_max_ = 456 nm and λ_onset_ = 430 nm, λ_max_ = 475 nm, at the time-delay of 0.8 ns vs 67.6 ns, respectively). Interestingly, the delayed fluorescence onset (at time-delay over 300 µs) matches with the prompt emission at late-time delays (>60 ns) and the steady-state emission spectrum (Supplementary Fig. 12b). The delayed fluorescence nature of this late-time emission is further confirmed by low-temperature time-resolved emission measurements where a highly red-shifted emission appears at an onset of 474 nm and this is assigned to be the phosphorescence band (Figure S12b). It is to be noted that even at a very low doping concentration (0.1 wt.%), a very similar spectral-shift was observed and hence, host-guest (exciplex) interactions (via the host Cz unit) can be envisaged that control the delayed fluorescence characteristics in these films (Supplementary Figs. 12c and 13b)^54^. Furthermore, ***o*-ICz-PI** behaves in a very similar manner to the ***p*-ICz-PI** in terms of delayed fluorescence characteristics (with ∆E_ST_ = 0.24 eV vs ∆E_ST_ = 0.27 eV, for ***p*-ICz-PI**) and a plausible host-induced exciplex formation in mCP, evident from their time-resolved emission profile (Supplementary Figs. 12d–f, 13a, c). These observations clearly show promising triplet harvesting properties with moderate photoluminescence quantum yields for ***o*-ICz-PI** (Supplementary Table 2), even in a small molecule semiconducting host. Lastly, to compare the triplet harvesting properties with a typical twisted donor-acceptor TADF design time-resolved emission measurements were performed with the control molecule, **Cz-PI** in 10 wt.% mCP host matrices. **Cz-PI** showed significantly smaller singlet-triplet gap in mCP (∆E_ST_ = 0.33 eV for 1 wt.% zeonex vs 0.18 eV for 10 wt.% mCP), resulting in relatively stronger delayed fluorescence (Supplementary Figs. 10b, d, 12g–i, 13a). It is worth mentioning that **Cz-PI** also showed strongly red-shifted emission in 10 wt.% mCP as compared to 0.1 wt.% doped films, suggesting that aggregation and intermolecular host-guest interactions effect the emission characteristics significantly (Supplementary Fig. 12i). PLQY in 10 wt.% mCP doped films range from low (Φ = 0.07 ± 0.05 for ***p*-ICz-PI**) to moderate (Φ = 0.23 ± 0.05 and Φ = 0.22 ± 0.05 for ***o*-ICz-PI** and **Cz-PI**) respectively, (Supplementary Table 2).

### Transient photoinduced absorption measurements

**Fig. 3:**
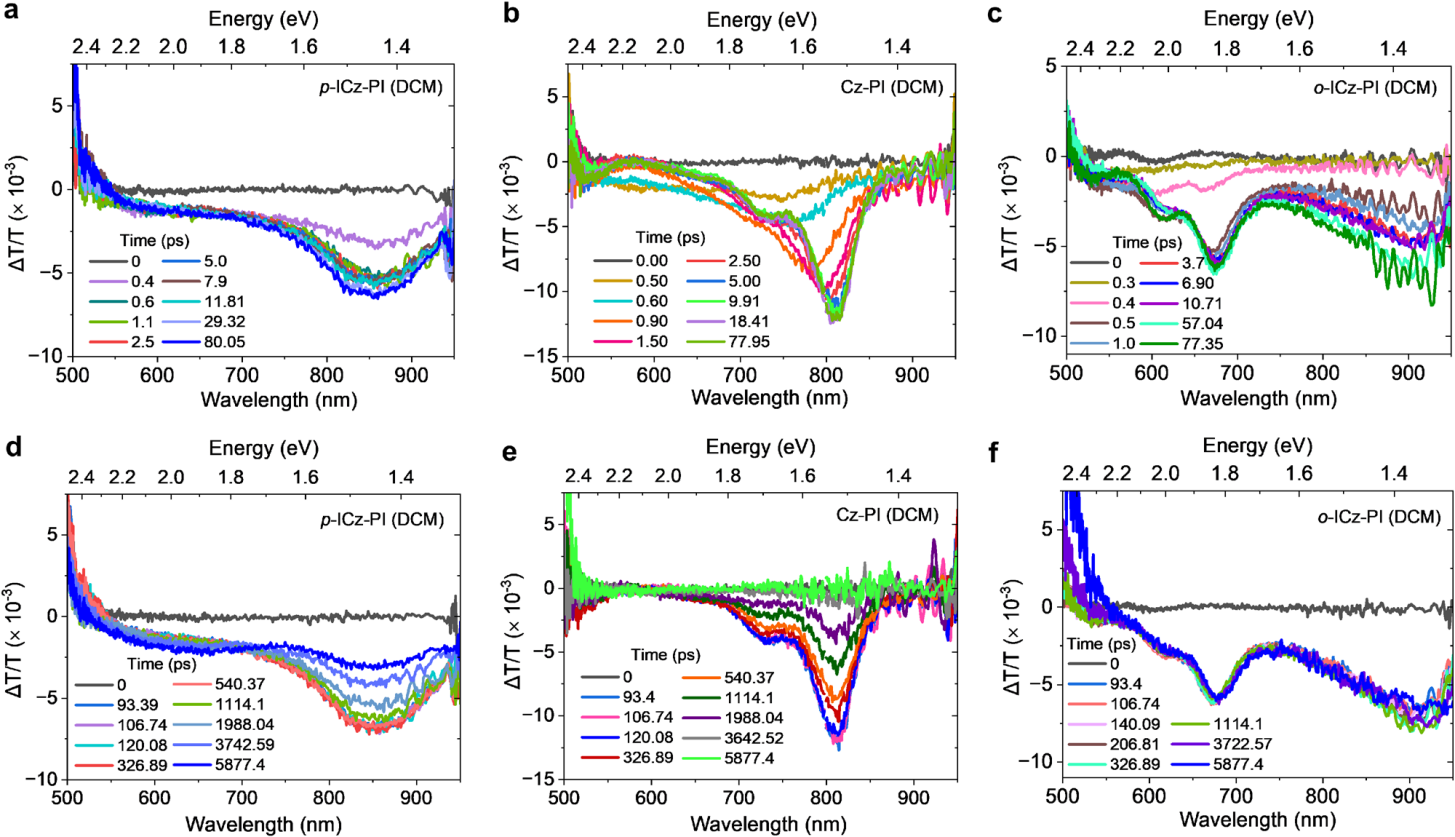
Early time evolution of excited state absorption spectra of *p* ***p*-ICz-PI**, ***o*-ICz-PI** and **Cz-PI** measured in DCM. Excited state absorption measurements in the 0–80 ps and 0–6 ns time windows for (**a**, **d**), ***p*-ICz-PI** (**b**, **e**) **Cz-PI** and (**c**, **f**) ***o*-ICz-PI** in polar DCM solutions showing the evolution of a stable CT state in ***o*-ICz-PI** and **Cz-PI** including relaxation of the CT state through solvent shell reorganisation. However, in ***p*-ICz-PI** very different spectral evolution shows much more locally-excited state characteristics from a large, conjugated fragment of the molecule. λ_exc_ = 343 nm, [c] = 1 × 10^−4^ M.

Transient absorption measurements were performed to study the initial excited state formation dynamics for all three derivatives in degassed solution state (DCM and MCH) (Fig. 3 and Supplementary Fig. 14). In MCH, ***p*-ICz-PI** gives an instantaneous excited state absorption (ESA) at 570 nm, followed by the emergence of an 870 nm band within 5 ps (Supplementary Fig. 15). The 870 nm ESA decays in 6.3 ns which correlates well with the fluorescence decay time of 6 ns (Supplementary Table 1), identifying this as the ESA from the emissive, highly delocalised and conjugated mixed LE/CT species. A residual ESA observed at 520–650 nm with a lifetime of 3.9 ns could potentially arise from the LE state as it is not observed in DCM. In DCM, the instantaneous 570 nm ESA band is again observed with a grow-in of ESA at 860 nm within 30 ps which decays with a lifetime of 6.7 ns (Supplementary Fig. 16), consistent with the fluorescence lifetime of 5.6 ns (Supplementary Table 1). The longer formation time could be due to the higher viscous drag of the DCM solvent shell reorganisation compared with MCH, and increased CT nature of the large delocalised mixed LE/CT excited state in DCM^55^. At the other extreme, **Cz-PI** in MCH (Supplementary Figs. 14 and 17) also has an instantaneous ESA at 560 nm followed by the emergence of a sharp ESA band at 750 nm within approximately 400 fs. This red ESA feature has a lifetime of 12.2 ns, matching closely to the fluorescence lifetime of 15.5 ns, indicating this band originates from the ICT species (Supplementary Table 1 and 3). We also observe a 10–15 nm relaxation of this state within its lifetime. This dispersion arises from dihedral angle inhomogeneity in a twisted D-A molecule, giving a broad-range of excited state lifetimes^56,57^. In DCM, the instantaneous 560 nm band was observed to decay very rapidly within 1 ps, with the formation of an initial ICT ESA at 730 nm, that rapidly relaxes to 820 nm within approximately 1.7 ps (Fig. 3 and Supplementary Fig. 18). This is consistent with the higher polarity and reorganisation of the DCM solvation shell (vide infra). The 820 nm ESA has a lifetime of 1.5 ns that matches the 1.5 ns fluorescence lifetime. Notably, from MCH to DCM a solvent shift of 60 nm is observed in the ICT ESA, which is less than ~100 nm solvatochromic shift observed in fluorescence. The band shape and position of this red ESA is very similar to previous observations of ESA from the ^1^(Cz)^+^ species^58,59^ which correlates with the expected Cz donor fragment in **Cz-PI** and is indicative of a full charge transfer state. This species is not observed in ***p-*ICz-PI**, indicating a different (very weak) donor structure.

Finally, the fast photo-dynamics of ***o*-ICz-PI** has signatures consistent with both the other molecules. In MCH, instantaneous ESA at 570 nm (which we correlate with the 410 nm shoulder observed in the ***o*-ICz-PI** fluorescence) proceeds to rapid formation of ESA at 650 nm in 1.7 ps that slowly relaxes to 660 nm within 10 ps (Supplementary Figs. 14 and 19). This state decays with a lifetime of 14 ns, in line with the fluorescence lifetime (Supplementary Table 1). Again, we associate this ESA feature with a localised ^1^(Cz)^+^ donor species^58,59^. Within approximately 1 ps, a long wavelength ESA emerges extending beyond 830 nm. This band decays in approximately 3.4 ns, indicating a different species as compared to the 660 nm ESA. In DCM (Fig. 3, Supplementary Fig. 20), ESA at 670 nm emerges within 700 fs, followed by ESA at 870 nm over approximately 20 ps. This mirrors to some degree the behaviour of ***p-*ICz-PI**. The slow decay of both the 670 nm (Supplementary Fig. 20b) band and the apparent lifetime of 32 ns for the 870 nm band matches the measured 35 ns fluorescence lifetime, indicating the presence of a long-lived ICT state. All lifetimes are collated in Supplementary Table 3. We thus ascribe the 870 nm ESA band to be from the delocalised cation species of the ***o-*ICz-PI** CT state, different to that in **Cz-PI**.

All three molecules have instantaneous ESA at 560–570 nm, ascribed to the LE state of the initially photoexcited fragment. This is seen to decay faster in more polar solution. The observed long-lived ESA of the ^1^(Cz)^+^ species in ***o*-ICz-PI** validates the description of ***o*-ICz-PI** as a planar rigidified molecule having an ICT excited state. As the dynamics and ESA spectrum of ***o*-ICz-PI** show part similarities to both ***p*-ICz-PI** and **Cz-PI** we believe this fully confirms our idea that breaking of conjugation forms spatially separated D and A intramolecular fragments on ***o*-ICz-PI**, leading to a stable ICT state. These observations are also corroborated with a detailed computational study reported in the next section.

### Electronic structure simulations

**Fig. 4:**
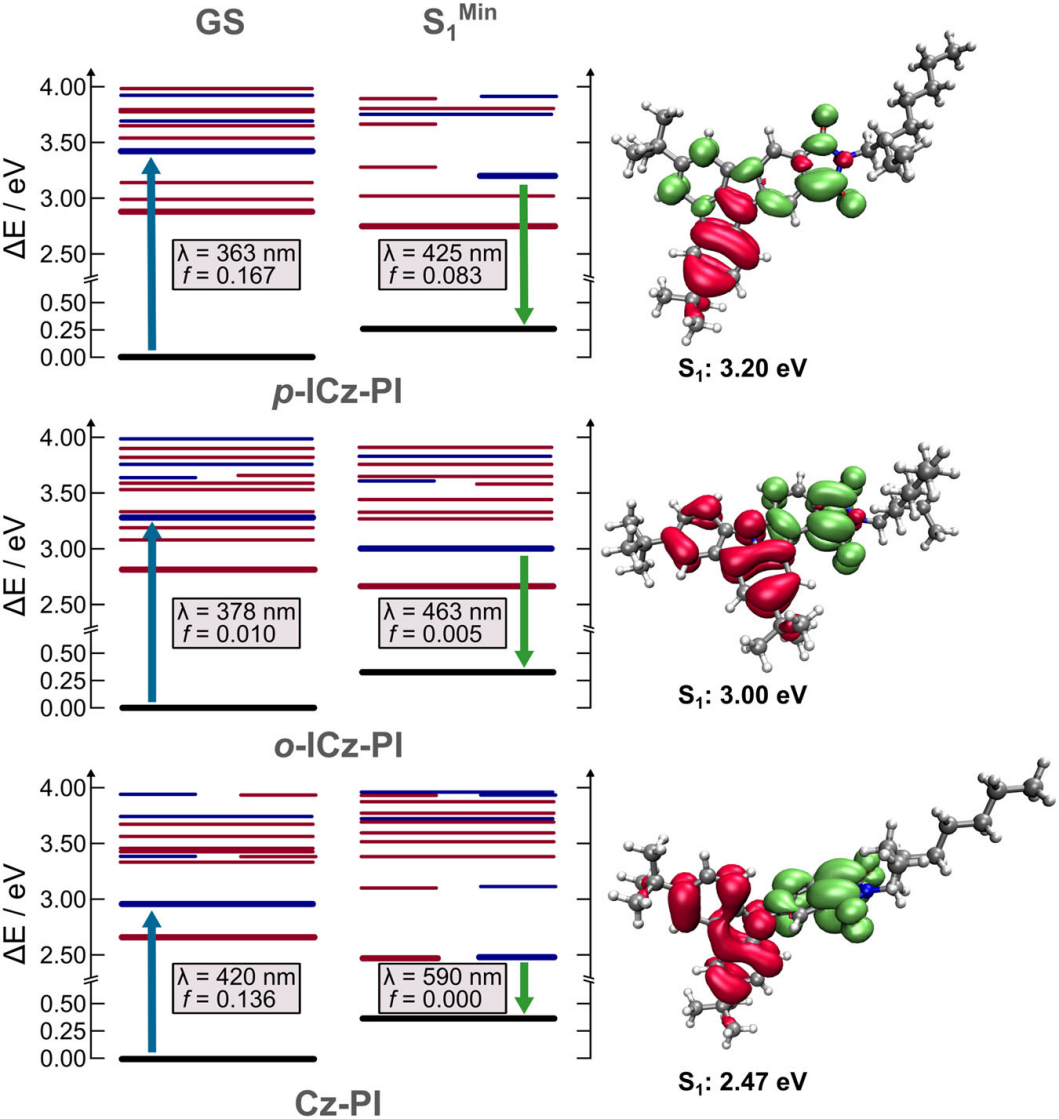
Theoretical energy level diagram with the difference of electronic density associated to the transition from the ground state to S1. Schematic of the low-lying excited states (red = triplet and blue = singlet) of ***p*-ICz-PI,** ***o*-ICz-PI and Cz-PI** at the ground and S_1_ excited state geometries. The electronic density differences (red is loss and green is gain in electron density upon excitation) associated with these transitions are shown on the right. Transition oscillator strengths are given alongside the calculated transition wavelength. All the energies are shown in Supplementary Tables 4, 9 and 14.

Figure 4 shows a schematic of the low-lying excited states (red = triplet and blue = singlet) of **Cz-PI,** ***o*-ICz-PI** and ***p*-ICz-PI** at the ground (S_0_) and S_1_ excited state geometries. The electronic density differences associated with the transition to the lowest excited singlet states are shown on the right. As expected, the orthogonally connected **Cz-PI** shows a strong CT transition from the carbazole donor to phthalimide acceptor. Importantly, despite their structural similarity ***o*-ICz-PI** and ***p*-ICz-PI** show distinct differences in their electronic distribution which is dependent on the bonding pattern between the donor and acceptor, consistent with the experimental differences discussed above.

For ***p*-ICz-PI**, the hole is primarily localized over the single phenyl ring of the *tert*-Cz unit, with some contribution existing in the PI unit (Fig. 4, Supplementary Figs. 21–26 and Supplementary Tables 4 and 6–8). The electron is delocalized over the remaining fragment of the molecule that consists of both the *tert*-Cz and PI units. This configuration results in a very weak “donor” unit, contributing little CT character to the excited state, thus the emission is blue and exhibits predominantly locally-excited (LE) character. In contrast, ***o*-ICz-PI** exhibits a hole that is primarily localized over the whole ICz unit, while the electron is concentrated over the PI unit. This arrangement resembles that of **Cz-PI**, which itself shows even stronger CT character because of the 90° twist between D and A at the N-C bridging bond.

It is remarkable that with effectively 0° twist i.e. fully planar, the D-A structure of ***o*-ICz-PI** exhibits such strong charge separation, which is consistent with the lower oscillator strength of absorption for ***o*-ICz-PI** (Fig. 4, Supplementary Figs. 27–32 and Supplementary Table 9 and Supplementary Figs. 11−13, resulting in a five-fold reduction in molar absorption coefficient compared to ***p*-ICz-PI** (vide infra). The calculated S_1_ ← S_0_ oscillator strength (OS) completely reflects this, showing significantly reduced OS for ***o*-ICz-PI** (f = 0.010), compared to ***p*-ICz-PI** (f = 0.167). Similarly, the S_0_ ← S_1_ oscillator strength decreases substantially in ***o*-ICz-PI** (f = 0.005) compared to ***p*-ICz-PI** (f = 0.083), indicating a stronger charge-transfer contribution in both the ground and excited states for ***o*-ICz-PI**. Spin-orbit coupling matrix elements (SOCMEs) amongst low lying electronic excited states are given in Supplementary Tables 5, 10 and 15 for ***p*-ICz-PI,** ***o*-ICz-PI** and **Cz-PI**, respectively. The SOCMEs between the lowest lying singlet and triplet states are found to be in the range of ~1 cm^-1^. We note that vibronic coupling may increase these couplings and might induce further enhancement through coupling to the high SOC rates observed between the T_4_ and T_5_ states.

**Fig. 5:**
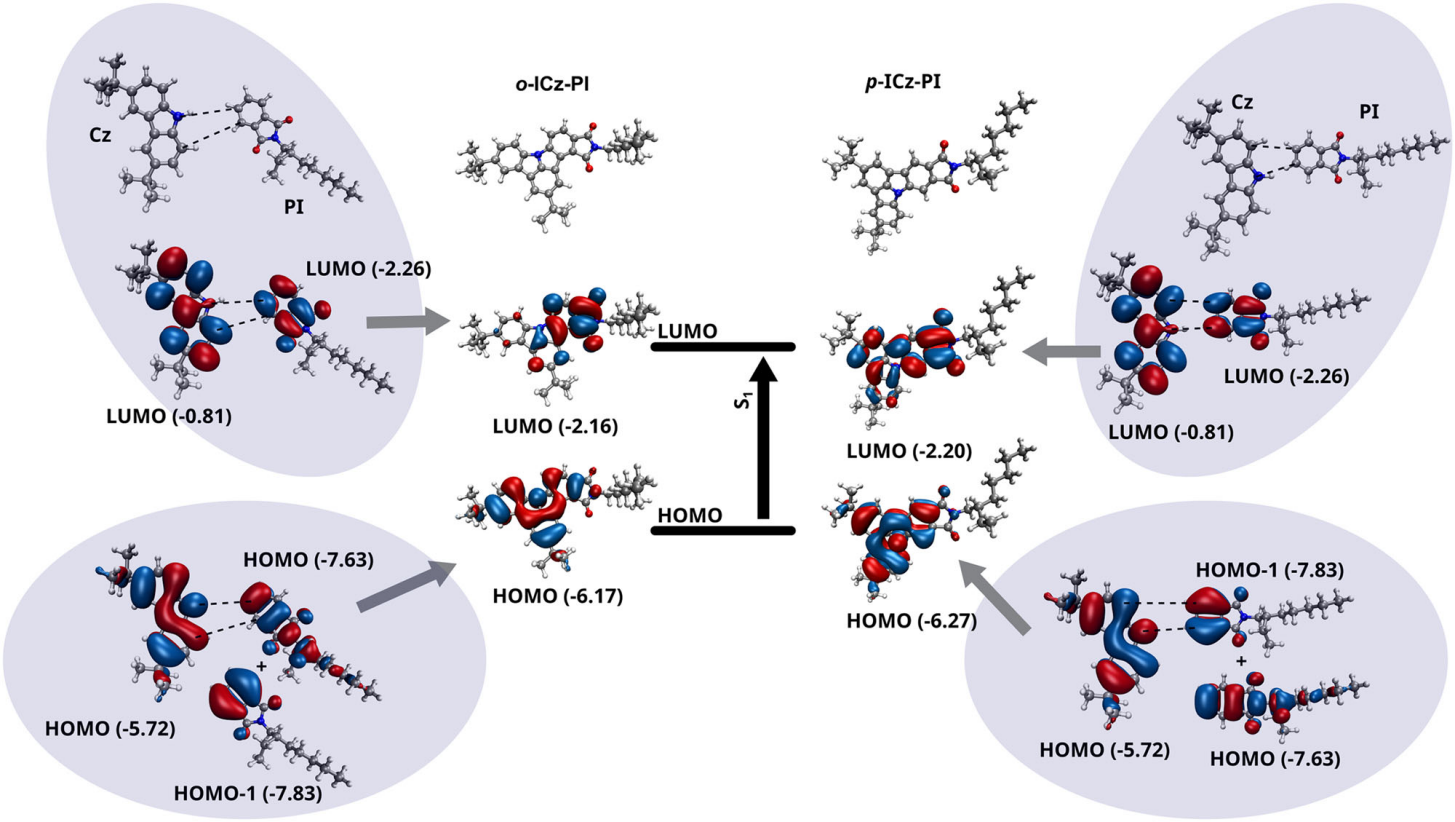
Orbital fragment analysis of ***o*-ICz-PI** and ***p*-ICz-PI** HOMO and LUMO levels. Fragment molecular orbital interaction for HOMOs and LUMOs of ***o*-ICz-PI** and ***p*-ICz-PI**. As shown in the supporting information, the S_1_ state is predominantly HOMO-LUMO character. The character of the HOMO does not change between the two isomers, but the delocalisation of the LUMO is controlled by the interaction between the two fragments. Orbital energies are given in bracket in eV.

To understand the stark difference between ***o*-ICz-PI** and ***p*-ICz-PI** we used the fragment orbital interaction approach that had previously characterized the competition between twisted and planar intramolecular charge transfer states in a range of D-A chromophores^60^. Fig. 5 shows the HOMO and LUMO molecular orbitals of ***o*-ICz-PI** and ***p*-ICz-PI** which are dominant in generating the low-lying excited states. For both isomers, the HOMO orbitals remain largely unchanged, while the LUMO orbitals exhibit a difference, becoming more localised on the acceptor for the ***o*-ICz-PI**. This can be explained by investigating how the LUMO is formed from each donor (Cz) and acceptor (PI) fragment. For ***p*-ICz-PI**, there is a bonding interaction across both bonding sites leading to a delocalisation of the LUMO across the whole molecule. In contrast, for ***o*-ICz-PI** there is an anti-bonding interaction across the N-C bond which enforces localisation of the LUMO onto the PI fragment increasing the CT character of the HOMO-LUMO transition. All the photophysics of the two **ICz-PI** isomers result from the different bonding pattern and separation of D and A fragments in the molecule.

These simulations demonstrate how sensitive the molecular electronic structure can be to bonding and anti-bonding patterns and that even in a fully-rigidified, co-planar D-A molecule, the CT state can be stabilised making it possible to achieve TADF, counter to previous thinking about these photophysical phenomena. Femtosecond induced absorption measurements further reveal ***o*-ICz-PI** to have an instantaneous induced absorption at 570 nm from an initial excited LE state; this is a more localised state compared to that seen in ***p-*ICz-PI**, rapid formation within 700 fs of a new state that absorbs at lower energy (at 660 nm) is observed which we ascribed to the formation of the CT excited state (Fig. 3 and Supplementary Fig. 14). In DCM, this state at 670 nm has much stronger intensity than in MCH, but the formation rate is slower, ascribed to inductively slow charge reorganisation due to the solvent shell inertia (Fig. 3 and Supplementary Fig. 14)^55^. The lifetime of the excited state is concomitant with the fluorescence lifetime measured. The induced absorption at 670 nm (in DCM) or 660 nm (in MCH) is never lost indicating a high level of electronic decoupling of the D and A fragments, independent of solvent polarity, indicating that the broken conjugation effect can fully stabilize the ICT excited state, as predicted by our calculations.

To conclude, we have successfully developed a novel rigidized planar charge-transfer design strategy for triplet harvesting in purely organic molecules. Importantly, these co-planar ICT states are highly sensitive to subtle differences in the bonding pattern between the donor and acceptor fragments and therefore through judicious choice of the connecting bonds, charge-transfer stabilization and access to the triplet excited state can be achieved without the need of a twisted excited geometry, previously considered essential for ICT molecules exhibiting TADF. Notably, these emitters are shown to be free of any dynamic charge-transfer state relaxation, typically associated with torsional motion between the donor-acceptor single bond leading to a dispersion of excited charge-transfer energy states and lifetimes. Consequently, development of these emitters could lead to materials with efficient triplet harvesting and highly stable charge transfer states yielding TADF with no dispersive (long-lifetime components) rISC. As recently shown, this is also essential for sensitisation in hyperfluorescent OLED applications and potential long device lifetime^23^.

This work opens a new paradigm for triplet harvesters exploiting rigidly planar charge-transfer design strategies. Developing this concept should focus upon identifying suitable donor-acceptor combinations which achieve smaller singlet-triplet gaps in low polarity environments and higher mono-dispersed reverse intersystem crossing rates, both of which will improve device performance. The similarity between the behaviour of the twisted D-A TADF emitter, **Cz-PI** and its highest performing rigidized planar variant, ***o*-ICz-PI** indicates that a large library of potential candidates based upon planarized versions of existing twisted D-A TADF emitters could be efficiently screened to identify high performing candidates. This will enable our planar rigidized charge-transfer design strategy to become a key focus for future molecular development for efficient triplet harvesting.

## Methods

### Synthesis

The detailed synthetic procedures for ***p*-ICz-PI,** ***o*-ICz-PI** and **Cz-PI**, their chemical structure characterizations including ^1^H NMR, ^13^C NMR, and high-resolution mass spectrometry can be found in Supplementary Information (Section 3) and Supplementary Figs. 39–52.

### Spectroscopic methods

#### Steady state measurements

Absorption, photoluminescence, and excitation measurements were obtained using drop-cast films on sapphire substrates at 1% by weight for zeonex (1 wt. %) and 10 wt. % by weight (10 wt. %) for the other hosts; for the solution measurement, concentrations of 20 μM were used. A Jobin Yvon Horiba Fluorolog (double-double) spectrofluorimeter and a Shimadzu UV–vis–NIR 3600 spectrophotometer were used for emission and absorption measurements, respectively. All spectral onset energies were corrected using the Jacobian conversion of wavelengths to energies.

#### Time-resolved measurements

The time-resolved measurements were obtained using a Stanford Computer Optics 4Picos gated iCCD camera (250–950 nm) with sub-nanosecond resolution and spectrograph equipped with 300 lines/mm grating, 500 nm blaze wavelength system. For the temperature-dependent measurements, a helium-closed cycle cryopump was used, equipped with optical windows, Si thermodiode, and sample mount, attached directly to the cold head. Excitation was from the 3rd or 4th harmonic of an EKSPLA 200 ps Nd:YAG 10 Hz repetition rate laser. Time-resolved measurements were made using a variable CCD gate and delay times relative to the laser trigger, allowing emission decays to be constructed from the changes in spectrum area (normalised by gate time). TCSPC measurements were recorded with a Horiba DeltaFlex TCSPC system using a Horiba NanoLED (357 nm) and SpectraLED (330 nm) as light sources. PLQY measurements were made using a HORIBA Fluorolog-QM spectrofluorometer equipped with a high-efficiency “ECO” friendly continuous 75 W Xenon arc lamp and a HORIBA integrating sphere. The excitation wavelength used was 375 nm, and the monitored emission depended on the molecule. The spectrofluorometer specific parameters were the excitation and emission slits and exits set to 1 nm, the step size for scanning the spectra was 1 nm, and integration time was 0.1 seconds. The PLQY was then calculated using the built in Quantum Yield Calculator of the spectrofluorometer.

#### Transient absorption spectroscopy

The laser used in the transient absorption setup is PHAROS from Light Conversion (wavelength: 1030 nm, pulse duration: 180 fs, pulse energy: 0.6 mJ, repetition rate: 1 KHz). Part of the output is used to do third harmonic generation (THG), which produces 343 nm output. We use this 343 nm output to pump our samples and the pulse energy used is about 0.5 μJ. Another part of the output is used to pump a 2 mm sapphire plate to generate white light continuum (WLC). We use this WLC to probe the dynamics of the excited states. The polarization of the pump is vertical to the optical table, while the polarization of the probe is parallel to the optical table. The spot sizes (FW @ 1/e^2^) of the pump and probe are 270 μm and 200 μm, respectively. The time delay between pump and probe is controlled by a 1-meter motorized translation stage (Zaber Technologies Inc. A-LST1000AKT07G06SU), which can generate about 6 ns delay range. The pump is modulated by an optical chopper (Thorlabs Inc. MC2000B-EC) that is locked to the half frequency of the laser repetition rate to create an iterated pump on / pump off situation. The WLC goes through a spectrometer and the intensities at different wavelengths are monitored by a camera (Imaging Solutions Group LightWise LW-ELIS-1024A-1394). The camera is synchronized with the laser pulse to ensure it captures the spectrum of WLC pulse by pulse. The transient absorption spectrum at one fixed time delay is calculated by ref. ^55^

1 $$\frac{\varDelta T}{T}=\left\{{\sum}_{i=1}^{N}\frac{{Spectru}{m}_{{pumpon}}-{Spectru}{m}_{{pumpoff}}}{{Spectru}{m}_{{pumpoff}}}\right\}/N$$

where *N* is average number, in our case *N* = 500. By changing the time delay between pump and probe, we obtain the whole transient absorption spectra.

### Computational methods

All calculations were performed using the ORCA 5 quantum chemistry package^61,62^.

Ground state and S_1_ and T_1_ minimum geometries were optimised using density functional (DFT) and time-dependent DFT (TD-DFT), respectively. The PBE0 functional^63^ was employed throughout in conjunction with the def2-TZVP basis set^64^ within the Tamm-Dancoff approximation^65^. All optimised geometries were confirmed to be minima of the potential energy surfaces by normal mode analysis. Calculations were performed in gas phase and methylcyclohexane (MCH), toluene and dichloromethane (DCM) solvent environments described using the conductor-like polarizable continuum medium (CPCM)^66^. XYZ Cartesian coordinates files are available on request.

## Supplementary information

Supplementary information

Transparent Peer Review file

## Data Availability

The data that support the plots and tables and conclusions in this work are given within the paper and supplementary information. The source data is available upon request from the corresponding author.
