# Supplementary material for: Rigid and planar π-conjugated molecules leading to long-lived intramolecular charge-transfer states exhibiting thermally activated delayed fluorescence
Source: Nat Commun. 2024 Nov 7;15:9611. doi: 10.1038/s41467-024-53740-1 (PMC11544105; doi:10.1038/s41467-024-53740-1)
Supplement: Supplementary file 1 — Supplementary information [file 41467_2024_53740_MOESM1_ESM.pdf]

Rigid and planar π-conjugated molecules leading to long-lived intramolecular charge-transfer states exhibiting thermally activated delayed fluorescence

Suman Kuila[1,2,4*], Hector Miranda-Salinas[1], Julien Eng[3], Chunyong Li[1], Martin R. Bryce[2], Thomas J. Penfold[3] and Andrew P. Monkman[1*]

[1]Department of Physics, [2]Department of Chemistry, Durham University, South Road, Durham, DH1 3LE, UK

[3]Department of Chemistry, School of Natural and Environmental Sciences, Newcastle University, Newcastle upon Tyne, NE1 7RU, UK

[4]Present Address: Renewable and Sustainable Energy Institute, University of Colorado Boulder, Boulder, Colorado 80309, United States

[*]E-mail: suman.kuila@colorado.edu, a.p.monkman@durham.ac.uk

Table of Content

1. General Experimental Details – Methods and Instruments. 2

2. Sample Preparation for Optical Measurements 2

3. Synthesis and Characterization of the Compounds 3

4. Optical Properties............ 6

5. Electronic Structure Simulations............ 24

6. Characterizations Spectra............ 56

7. References............ 65

1. General Experimental Details – Methods and Instruments.

Commercial reagents were purchased and used without further purification. Reactions were conducted under an argon atmosphere, unless otherwise stated. Glassware were dried overnight in an oven at 80 °C. Solvents and liquid reagents were added by syringe or cannula, and solid reagents were added under a positive pressure of argon. Degassing was performed by bubbling argon through the reaction mixture using an argon-filled balloon fitted with a syringe needle. Thin layer chromatography (TLC) analysis was performed by using Merck Silica gel 60 F254 TLC plates and spots were visualized by UV irradiation at 365 and 254 nm. Column chromatography was performed using silica gel 60 purchased from Fluorochem. NMR spectroscopy was carried out on Bruker AV400, Varian VNMRS 600 and 700 spectrometers. Spectra were recorded at 295 K in commercially available deuterated solvents and referenced internally to the residual solvent proton resonances.[1] Atmospheric pressure solids analysis probe (ASAP) ionization mass spectra were obtained using an LCT Premier XE mass spectrometer and an Acquity® UPLC from Waters Ltd at 350°C. High-resolution mass spectrometry was carried out on a Quantum time-of-flight (QToF) mass spectrometer.

2. Sample Preparation for Optical Measurements.

Solutions (in methylcyclohexane (MCH), toluene, and dichloromethane (DCM) of all the studied samples for photophysical characterization were prepared at low concentration of 2.5-20 μM to strictly prevent intermolecular interactions. Degassed solutions were obtained by 5 freeze-pump-thaw cycles to remove all dissolved oxygen. Solid state samples were fabricated by drop casting onto quartz. To prepare the 10 wt. % doped films of emitters in a host matrix, 90 % w/w (0.9 mg) of the host was dissolved in 0.1 mL of solvent and to this was added 10 % w/w (0.1 mg) of the emitter.

3. Synthesis and Characterization of the Compounds.

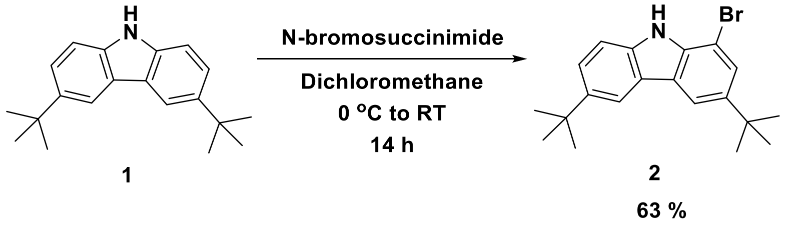

Scheme 1. Synthesis of **2**.

Compound 2 was synthesized according to a literature procedure with slight modification and the characterization agrees with the previous report.[2]

In a 250 mL 2-necked round bottomed flask 3,6-di-*tert*-butyl-9*H*-carbazole (**1**, 5 g, 17.9 mmol) was dissolved in dichloromethane (75 mL). Then a solution of *N*-bromosuccinimide (NBS) (3.5 g, 19.7 mmol) in dichloromethane (75 mL) was added dropwise using a dropping funnel at 0 °C. The reaction mixture was slowly warmed to room temperature and stirred for 14 h in the dark. When the reaction was completed, saturated sodium thiosulfate solution (100 mL) was added to quench the excess NBS. This mixture was then extracted with dichloromethane (3 x 60 mL). The combined organic extracts were washed with water (200 mL) and dried over $MgSO_4$, filtered and concentrated in vacuo. A colorless, sticky liquid was obtained, which was adsorbed onto silica gel and purified by column chromatography by using dichloromethnae/hexane, 1/9 to 1/5, v/v, solvent mixture to obtain **2** as a glassy solid. Yield of **2** = 4.1 g (63 %). ^{1}H NMR (400 MHz, $CDCl_3$) δ 8.05-8.01 (m, 3H), 7.60 (d, *J* = 1.6 Hz, 1H), 7.51 (dd, *J* = 8.6, 1.9 Hz, 1H), 7.40 (dd, *J* = 8.6, 0.6 Hz, 1H), 1.44 (s, 18H). ^{13}C NMR (101 MHz, $CDCl_3$) δ 144.05, 142.93, 137.65, 136.65, 125.76, 124.58, 124.38, 123.63, 116.68, 115.49, 110.48, 103.67, 34.90, 34.77, 32.00.

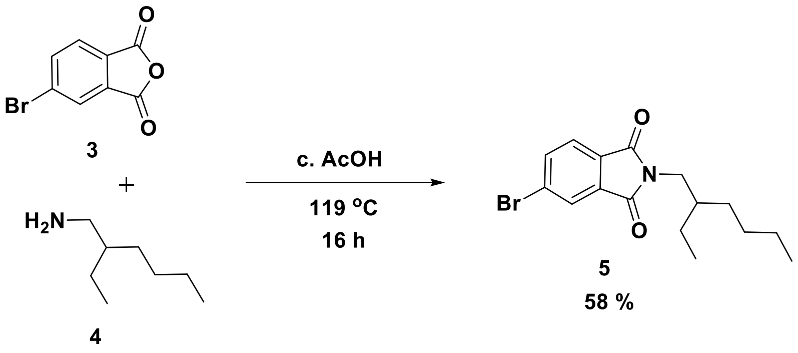

Scheme 2. Synthesis of **5.**

Compound 5. 4-bromophthalic anhydride (**3**, 4.5 g, 19.8 mmol) was added into a 100 mL round bottom flask containing glacial acetic acid (30 mL). 2-Ethylhexyl amine (2.94 g, 22.8 mmol) was added into this suspension after 5 minutes. The reaction mixture was refluxed at 119 °C for 16 h. Upon completion of the reaction, the reaction mixture was cooled to room temperature and slowly added to water (200 mL) to obtain a white precipitate. The precipitate was filtered from the solution and washed with water (300 mL). The resulting solid was dried in air and then under vacuum to obtain **5** as a white powder. This was used in the next step without further purification. The characterization data matches well with the previous report.[3]

Yield of **5** = 3.9 g (58 %, white solid). ^{1}H NMR (400 MHz, $CDCl_3$) δ 7.97 (d, *J* = 1.6 Hz, 1H), 7.84 (dd, *J* = 7.9, 1.7 Hz, 1H), 7.70 (d, *J* = 7.9 Hz, 1H), 3.57 (d, *J* = 7.3 Hz, 2H), 1.81 (m, 1H), 1.40 – 1.17 (m, 8H), 0.90 (t, *J* = 7.6 Hz, 6H), 0.87 (t, *J* = 6.4 Hz, 3H). ^{13}C NMR (101 MHz, $CDCl_3$) δ 167.92, 167.37, 136.86, 133.75, 130.62, 128.78, 126.59, 124.57, 42.14, 38.25, 30.48, 28.48, 23.81, 23.00, 14.08, 10.40. HRMS-ESI$^+$ *m/z* calculated for $[M+H]^+$ $C_{16}H_{21}NO_2Br$, 338.0777; found: 338.0772.

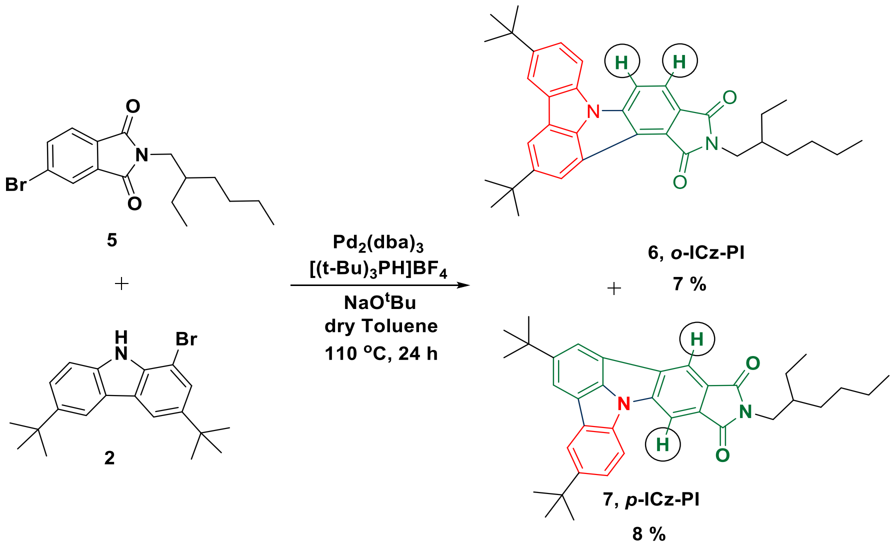

Scheme 3. Synthesis of ***o*-ICz-PI** and ***p*-ICz-PI.**

p (-para) and *o* (-ortho) are named based on the positions of hydrogen atoms in the phthalimide (PI) units for the ***p*-ICz-PI** and ***o*-ICz-PI**; ICz stands for indolocarbazole.

Synthesis of *p*-ICz-PI (7) and *o*-ICz-PI (6). An oven-dried 2-necked 100 mL round bottom flask was degassed under vacuum and backfilled with argon, which was repeated 3-times. Compounds **2** (300 mg, 0.84 mmol) and **5** (312 mg, 0.92 mmol) were transferred to the flask and kept under high vacuum for 10 min, then refilled with Ar gas. Dry toluene (20 mL) was added to the flask and Ar gas was bubbled through the solution for 20 min. $Pd_2(dba)_3$ (77 mg, 0.084 mmol) and $[(t\text{-}Bu)_3PH]BF_4$ (49 mg, 0.17 mmol) were then added to the flask and Ar gas was bubbled for another 15 minutes. NaO*t*Bu (323 mg, 3.36 mmol) was added to the reaction mixture and the resulting solution was heated to reflux at 110 °C for 24 h. The solvent was evaporated on a rotary evaporator and the resulting solid was adsorbed onto silica gel and purified by column chromatography by using dichloromethane/hexane, 1/20 to 1/5, v/v, solvent

mixture. Yield of ***p*-ICz-PI** = 35 mg (8 %). R_f = 0.15 (DCM/hexane, 1/5, v/v); ***o*-ICz-PI** = 29 mg (7 %). R_f = 0.14 (DCM/hexane, 1/5, v/v).

***p*-ICz-PI** (light green solid): ^{1}H NMR (400 MHz, $CDCl_3$) δ 8.55 (d, *J* = 0.8 Hz, 1H), 8.26 (d, *J* = 0.7 Hz, 1H), 8.24 (d, *J* = 1.0 Hz, 1H), 8.19 (d, *J* = 1.1 Hz, 1H), 8.18 (d, *J* = 2 Hz, 1H), 7.88 (d, *J* = 8.4 Hz, 1H), 7.65 (dd, *J* = 8.5, 1.9 Hz, 1H), 3.65 (d, *J* = 7.3 Hz, 2H), 1.92 (m, 1H), 1.58 (s, 9H), 1.49 (s, 9H), 1.43 – 1.28 (m, 9H), 0.95 (t, *J* = 7.4 Hz, 3H), 0.90 (t, *J* = 6.8 Hz, 3H). ^{13}C NMR (101 MHz, $CDCl_3$) δ 169.10, 169.07, 148.34, 146.56, 144.15, 141.12, 136.77, 134.28, 130.51, 130.26, 124.85, 124.48, 120.13, 119.16, 118.92, 118.22, 117.28, 116.82, 112.19, 107.03, 42.09, 38.41, 36.10, 35.05, 32.72, 31.83, 30.57, 28.57, 23.91, 23.07, 14.12, 10.49. HRMS-ASAP$^+$ *m/z* calculated for $C_{36}H_{43}N_2O_2$ $[M+H]^+$ 535.3325; found: 535.3311.

***o*-ICz-PI** (off-white solid): ^{1}H NMR (400 MHz, $CDCl_3$) δ 8.72 (d, *J* = 1.3 Hz, 1H), 8.27 (d, *J* = 1.2 Hz, 1H), 8.19 (d, *J* = 1.6 Hz, 1H), 8.05 (d, *J* = 8.1 Hz, 1H), 7.96 (d, *J* = 8.0 Hz, 1H), 7.84 (d, *J* = 8.5 Hz, 1H), 7.63 (dd, *J* = 8.5, 1.9 Hz, 1H), 3.68 (d, *J* = 7.4 Hz, 2H), 1.96 (m, 1H), 1.61 (s, 9H), 1.49 (s, 9H) 1.50 – 1.26 (m, 9H), 0.96 (t, *J* = 7.4 Hz, 3H), 0.90 (t, *J* = 6.8 Hz, 3H). ^{13}C NMR (101 MHz, CDCl3) δ 169.61, 169.08, 148.42, 146.34, 145.00, 142.46, 136.62, 130.59, 127.55, 126.02, 124.61, 124.51, 121.14, 120.75, 120.07, 119.25, 118.61, 115.32, 115.02, 112.05, 42.11, 38.34, 36.10, 35.03, 32.77, 31.85, 30.54, 28.57, 23.85, 23.11, 14.12, 10.50. HRMS-ASAP$^+$ *m/z* calculated for $C_{36}H_{43}N_2O_2$ $[M+H]^+$ 535.3325; found: 535.3309.

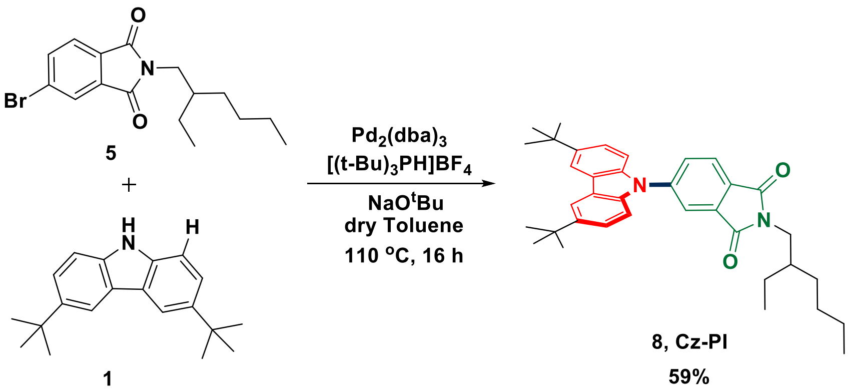

Scheme 4. Synthesis of **Cz-PI**.[4]

In **Cz-PI**, Cz stands for Carbazole and PI stands for phthalimide.

Synthesis of Cz-PI (8). An oven-dried 2-necked round bottom flask equipped with a reflux condenser was degassed under vacuum and backfilled with argon. This process was then repeated 3-times before transferring compounds **1** (136 mg, 0.49 mmol) and **5** (150 mg, 0.44 mmol) to the flask. This was kept under high vacuum for another 10 min, followed by refilling with Ar gas. Dry toluene (20 mL) was added to the flask and spurged with Ar gas for 20 min. $Pd_2(dba)_3$ (40 mg, 0.044 mmol) and $[(t\text{-}Bu)_3PH]BF_4$ (26 mg, 0.088 mmol) were then added to the flask and spurging with Ar gas was repeated for another 15 minutes. NaO*t*Bu (64 mg, 0.66 mmol) was added to the reaction mixture and the resulting solution was refluxed at 110 °C for 16 h. The solvent was evaporated on a rotary evaporator. The resulting solid was adsorbed onto silica gel and purified by column chromatography by using EtOAc/hexane, 1/30 to 1/10, v/v, solvent mixture to obtain **Cz-PI** as a light brown solid. Yield of **Cz-PI** = 141 mg (59 %). R_f =

0.3 (EtOAc/hexane, 1/20, v/v). **Cz-PI** : ^{1}H NMR (400 MHz, $CDCl_3$) δ 8.14 (dd, *J* = 2.0, 0.7 Hz, 2H), 8.08 (dd, *J* = 1.9, 0.6 Hz, 1H), 8.04 (dd, *J* = 7.9, 0.6 Hz, 1H), 7.93 (dd, *J* = 7.9, 1.8 Hz, 1H), 7.50 (dd, *J* = 8.7, 1.9 Hz, 2H), 7.43 (dd, *J* = 8.7, 0.6 Hz, 2H), 3.64 (d, *J* = 7.2 Hz, 2H), 1.89 (m, 1H), 1.47 (s, 18H), 1.44 – 1.28 (m, 9H), 0.96 (t, *J* = 7.4 Hz, 3H), 0.92 (t, *J* = 6.8 Hz, 3H). ^{13}C NMR (101 MHz, $CDCl_3$) δ 168.08, 168.02, 144.25, 143.97, 138.31, 134.27, 130.78, 129.17, 124.82, 124.17, 124.10, 120.50, 116.57, 109.13, 42.18, 38.39, 34.82, 31.95, 30.57, 28.55, 23.88, 23.05, 14.11, 10.46. HRMS-ASAP$^+$ *m/z* calculated for $[M+H]^+$ $C_{36}H_{43}N_2O_2$, 537.3467; found, 537.3481.

4. Optical Properties

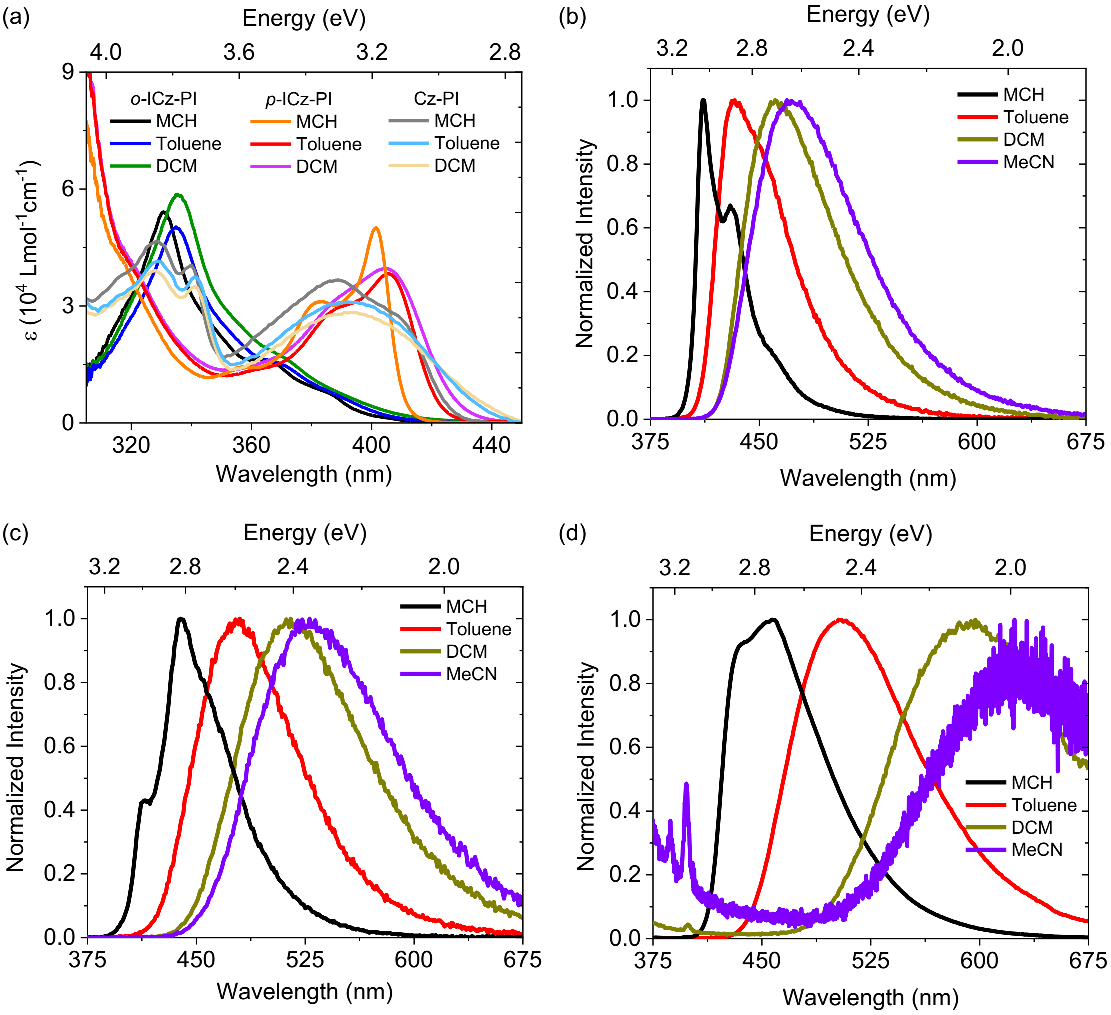

Supplementary Figure 1. Absorption and fluorescence of the investigated molecules. (a) Absorption spectra and molar absorption co-efficient of **Cz-PI**, ***o*-ICz-PI** and ***p*-ICz-PI**. Normalized emission spectra of (b) ***p*-ICz-PI**, (c) ***o*-ICz-PI** and (d) **Cz-PI** at different solvent polarities**.** (λ_{exc} = 355 nm, [c] = 2 × 10^{-5} M).

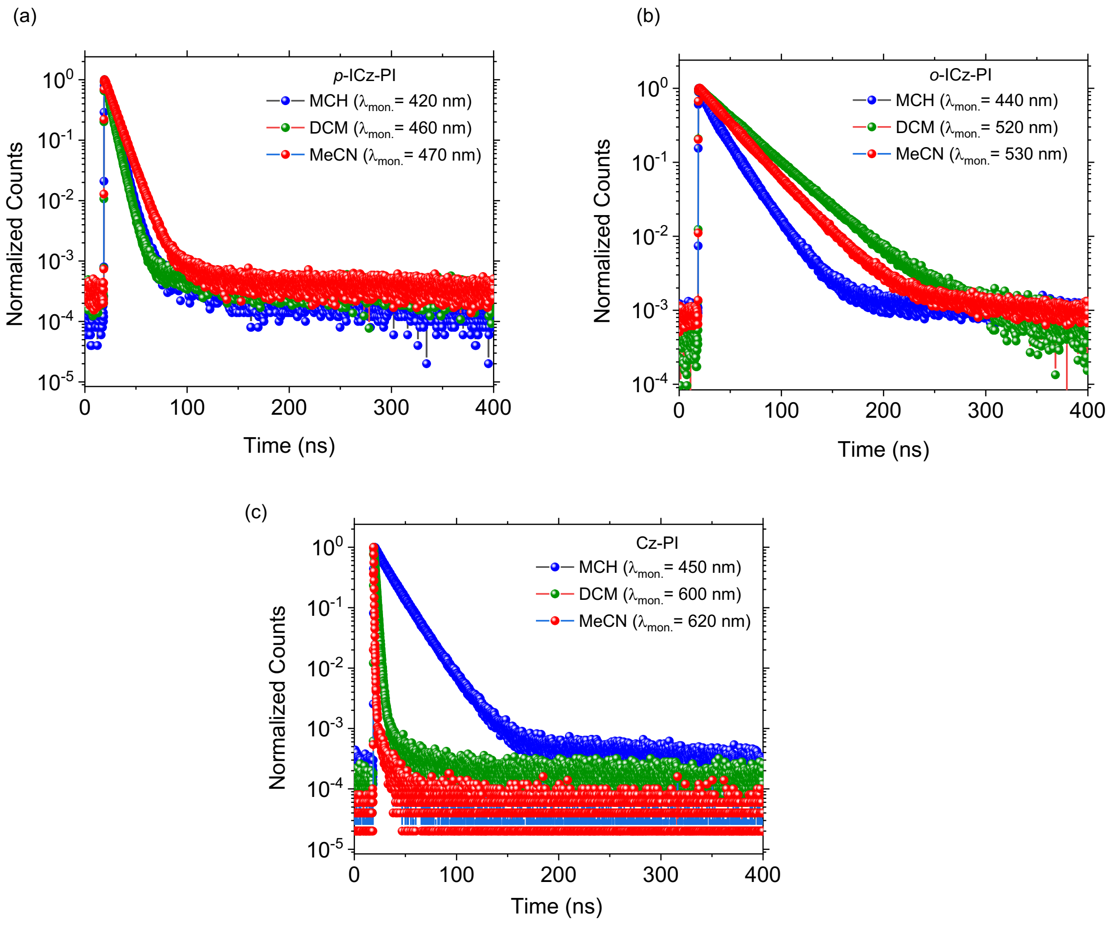

Supplementary Figure 2. Lifetime decays of the investigated molecules. Fluorescence lifetime decay spectra of (a) ***p*-ICz-PI**, (b) ***o*-ICz-PI** and (c) **Cz-PI** at different solvent polarities (λ_{exc} = 340 nm, [c] = 2 × 10^{-5} M, mon. is monitored).

Supplementary Table 1. Summary of time-correlated single photon counting (TCSPC) lifetimes in degassed solutions state and PLQY in air.

Molecule	Solvent	$\lambda_{monitored}$	Average lifetime (ns)	PLQY in air (λ_{exc} = 355 nm)
***p*-ICz-PI**	MCH	420 nm	6.1	0.38±0.05
	DCM	460 nm	5.6	0.41± 0.05
	Acetonitrile	470 nm	8.9	0.58± 0.05
***o*-ICz-PI**	MCH	440 nm	19.04	0.23± 0.05
	DCM	520 nm	35.7	0.24± 0.05
	Acetonitrile	530 nm	28.4	0.17± 0.05
**Cz-PI**	MCH	450 nm	15.5	0.48± 0.05
	DCM	600 nm	1.7	<0.04

	Acetonitrile	620 nm	0.28	<0.01

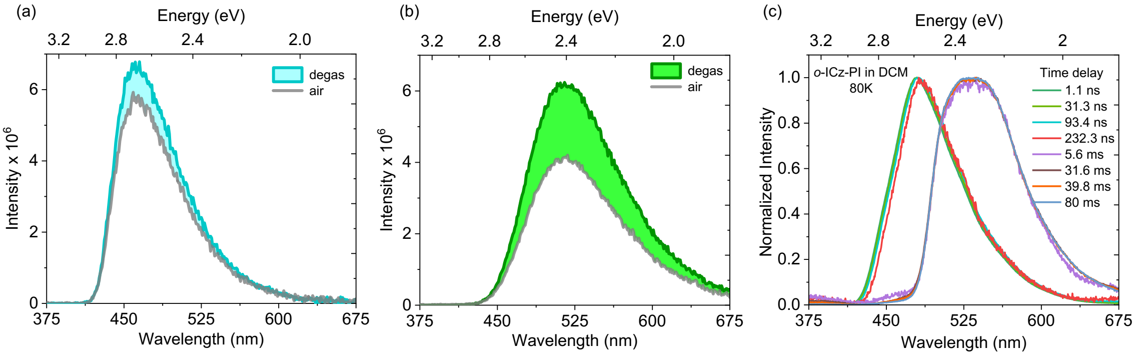

Supplementary Figure 3. Triplet stability studies in dichloromethane for *p*-ICz-PI and ***o*-ICz-PI.** Steady-state emission spectra of (a) ***p*-ICz-PI** and (b) ***o*-ICz-PI** in dichloromethane at room temperature measured in air and degassed conditions. (c) Time-resolved emission spectra at 80 K for ***o*-ICz-PI** in dichloromethane. (λ_{exc} = 355 nm. [c] = 2 × 10^{-5} M).

Supplementary Table 2. Summary of photophysical properties of all compounds.[5]

Solution State ([c]=2 × 10^{-5} M).[a)]

Emitters	τ_{PF} (ns)	τ_{DF} (μs)	k_F (×10^7 s^{-1})	k_{ISC} (× 10^7 s^{-1})	k_{rISC} (× 10^4 s^{-1})	Φ_{PL} (%) In air	Φ_{PL} (%) In N_2	S_1 (eV)	ΔE_{ST} (eV)
***p*-ICz-PI** (DCM)	5.6	66.9	17.9	9.54	3.21	0.41± 0.05	0.47± 0.05	2.93	0.29
***o*-ICz-PI** (DCM)	35.7	83.2	2.8	1.7	3.05	0.24± 0.05	0.37± 0.05	2.75	0.05
**Cz-PI** (Toluene)	14.5	20.7	6.9	4.21	1.24	0.39± 0.05	0.61± 0.05	2.80	0.1

Film State[b)]

Emitters	τ_{PF} (ns)	τ_{DF} (μs)	k_F (×10^7 s^{-1})	k_{ISC} (× 10^7 s^{-1})	k_{rISC} (× 10^1 s^{-1})	Φ_{PL} (%)	S_1 (eV)	T_1 (eV)	ΔE_{ST} (eV)
***p*-ICz-PI** (1 wt.% zeonex)	8.08	28.4	12.4	3.68	35.3	0.24± 0.05	3.01	2.64	0.37
***p*-ICz-PI** (10 wt.% mCP)	8.04	9.11	12.4	1.13	11	0.07± 0.05	2.88	2.61	0.27
***o*-ICz-PI** (1 wt.% zeonex)	22.7	159	4.41	3.08	0.63	0.17± 0.05	3.03	2.70	0.33

***o*-ICz-PI** (10 wt.% mCP)	30.6	42.1	3.27	0.07	2.37	0.23± 0.05	2.88	2.64	0.24
**Cz-PI** (1 wt.% zeonex)	11.4	5.66	8.77	18.5	17.7	0.43± 0.05	2.99	2.70	0.29
**Cz-PI** (10 wt.% mCP)	13.3	2.34	7.52	0.13	42.7	0.22± 0.05	2.76	2.58	0.18

a: $k_f = \frac{\Phi_{PF}}{\tau_{PF}}$; $k_{ISC} = \frac{1}{\tau_{PF}} \frac{\Phi_{DF}}{\Phi_{PF}+\Phi_{DF}}$; $k_{rISC} = \frac{1}{\tau_{DF}} \frac{\Phi_{PF}+\Phi_{DF}}{\Phi_{PF}}$

b: $k_f = \frac{\Phi_{PF}}{\tau_{PF}}$; $k_{rISC} = \frac{1}{\tau_{DF}}$; $\frac{k_{rISC}}{k_{ISC}} = \frac{1}{3}\, exp\left(\frac{-\Delta E_{ST}}{k_B T}\right)$

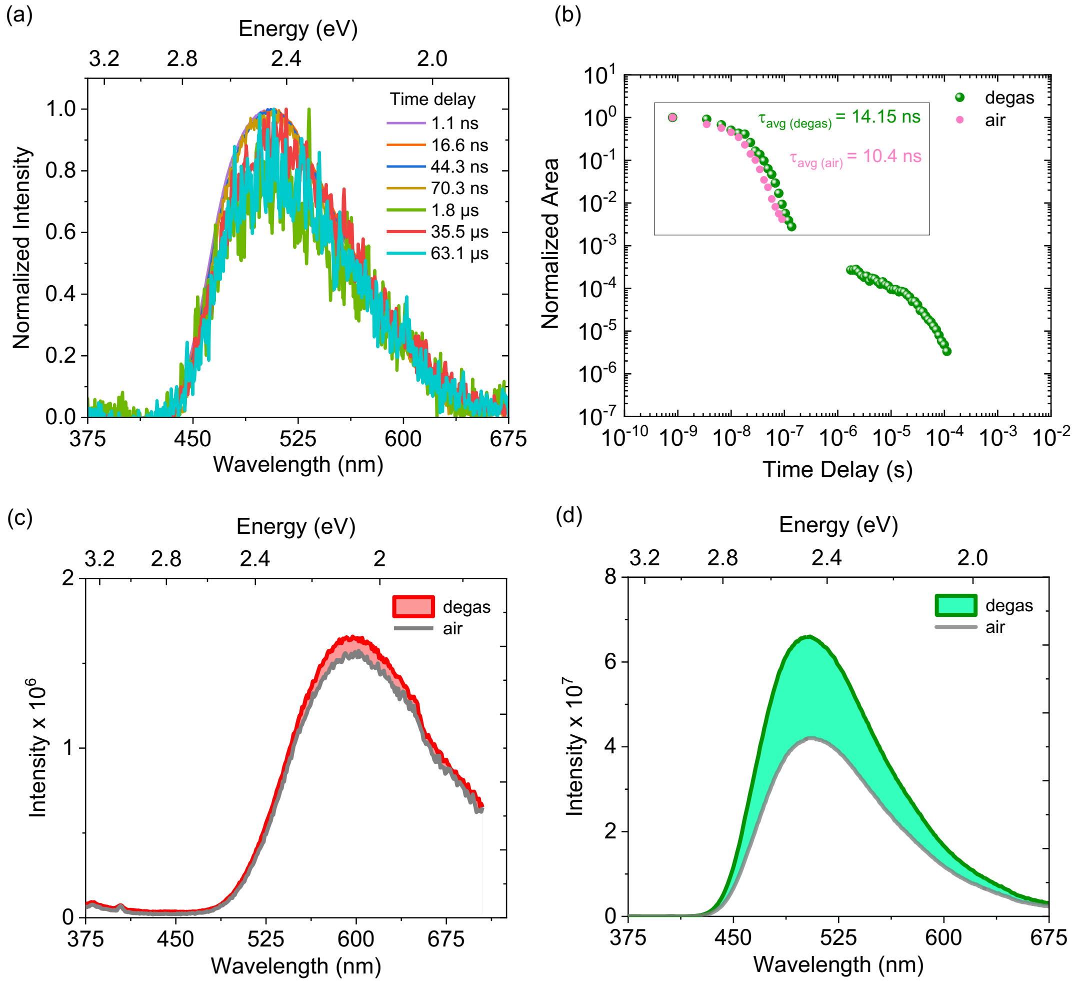

Supplementary Figure 4. Triplet stability studies in toluene and dichloromethane for Cz-PI. (a) Time-resolved emission spectra and (b) time-resolved decay of **Cz-PI** in degassed toluene at room temperature. ($\lambda_{exc.}$ = 355 nm, [c] = 2 × 10^{-5} M). Toluene was used to ensure a smaller ΔE_{ST} as compared to dichloromethane (Figure 1b). Steady-state emission spectra of **Cz-PI** in (c) dichloromethane and (d) toluene measured in air and degassed conditions. (λ_{exc} = 355 nm. [c] = 2 × 10^{-5} M). Lifetime quenching (10.4 ns/14.15 ns=0.73) is observed so that the PLQY

increase upon degassing is partly due to increased prompt singlet emission (Supplementary Figures 3 and 4).

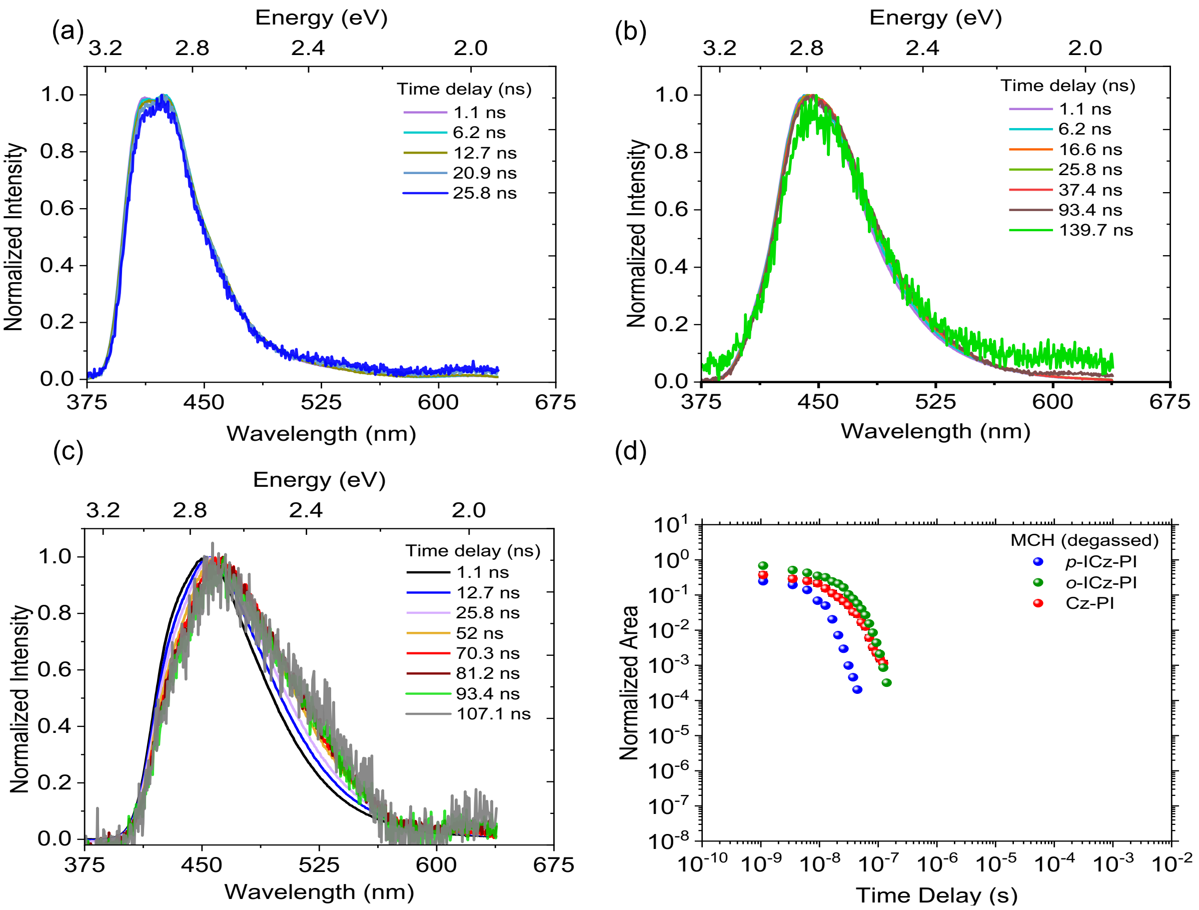

Supplementary Figure 5. Photophysical studies in methylcyclohexane. Prompt fluorescence decay of (a) ***p*-ICz-PI** (b) ***o*-ICz-PI** and (c) **Cz-PI** in degassed non-polar solution (methylcyclohexane) at room temperature, showing mixed LE/CT nature of the ***o*-ICz-PI** in its excited state as compared to ***p*-ICz-PI** having predominantly LE character. (d) Time-resolved decay profile of the same samples. (λ_{exc} = 355 nm, [c] = 2 × 10^{-5} M).

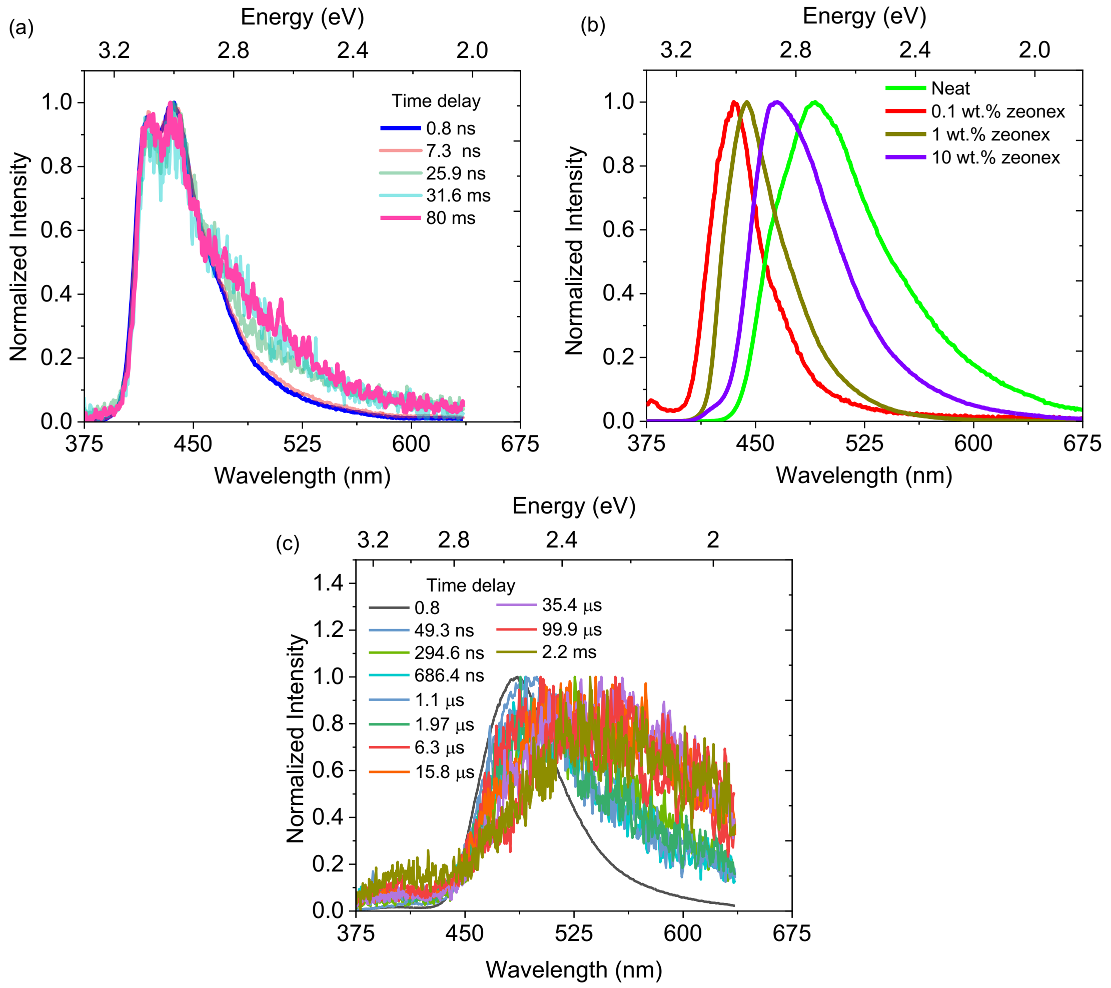

Supplementary Figure 6. Photophysical studies on *p*-ICz-PI films doped in zeonex and neat state. (a) Time-resolved emission spectra of 0.1 wt.% zeonex films doped with ***p*-ICz-PI** at RT. (b) Steady-state emission spectra of ***p*-ICz-PI** in neat and doped zeonex films at different weight ratios showing increasing aggregation features at higher doping percentages. Strong aggregation feature corroborated with 10 wt.% zeonex doped as well as neat films where the emission onset is highly red-shifted with a clear spectral broadening. (λ_{exc} = 355 nm). (c) Time-resolved emission spectra of ***p*-ICz-PI** in neat film at RT.

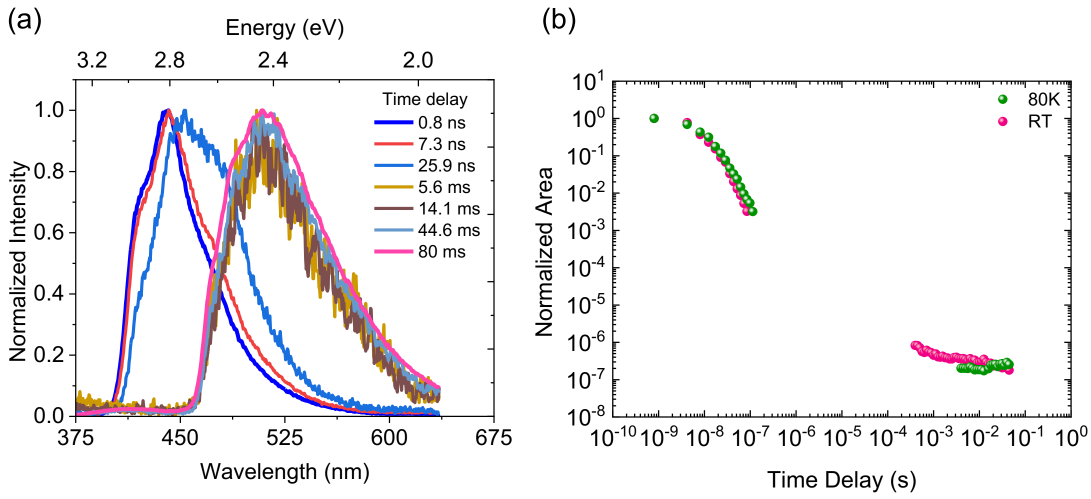

Supplementary Figure 7. Temperature dependent emission measurements 1 wt.% zeonex films doped with *p*-ICz-PI. (a) Time-resolved emission spectra at 80 K and (b) time-resolved decay spectra at room temperature and 80 K for 1 wt.% zeonex films doped with ***p*-ICz-PI**. (λ_{exc} = 355 nm).

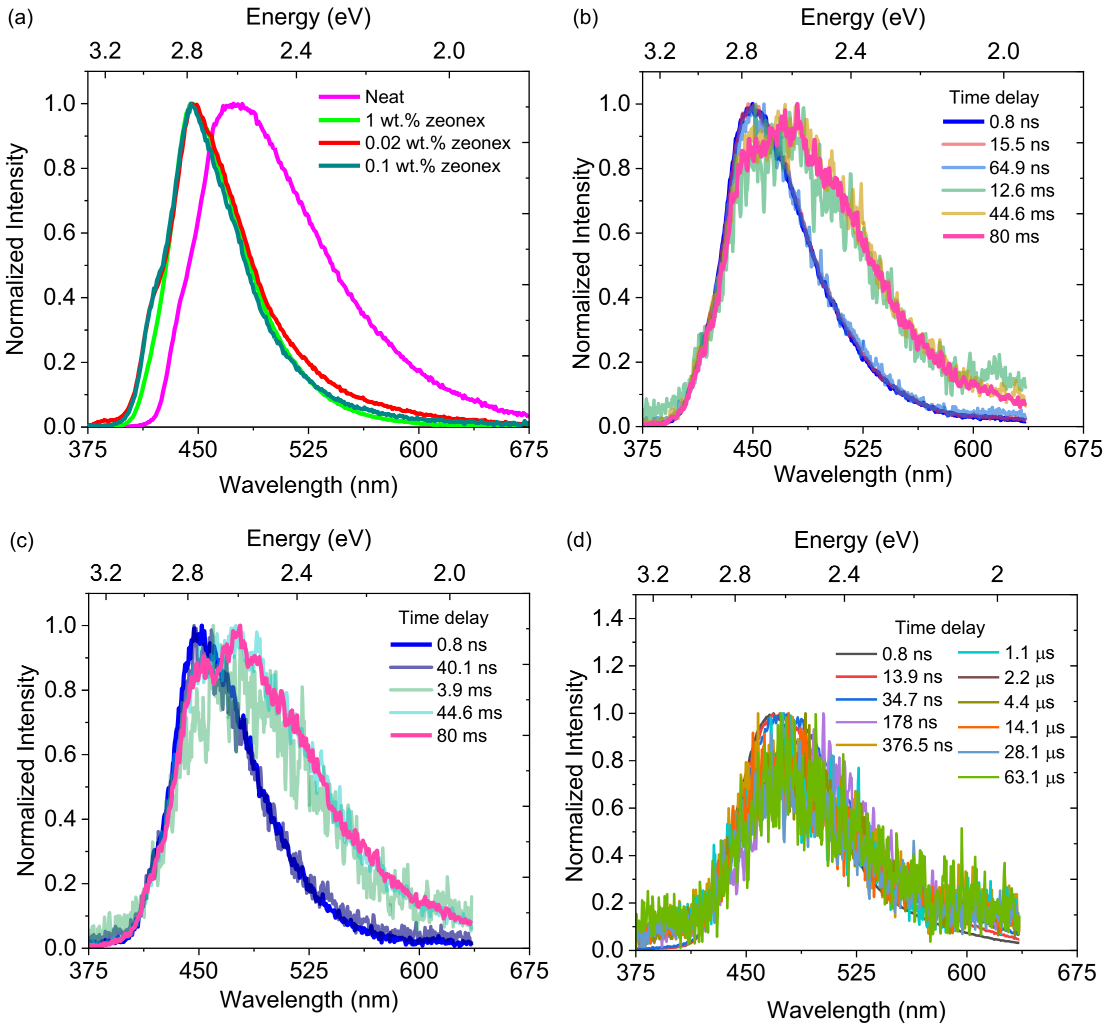

Supplementary Figure 8. Photophysical studies on *o*-ICz-PI films doped in zeonex and neat state. (a) Steady-state emission spectra of ***o*-ICz-PI** in doped zeonex films at different weight ratios and in neat films, showing no aggregation feature at higher doping percentages. Time-resolved emission spectra of (b) 0.02 wt.% and (c) 0.1 wt.% zeonex films doped with ***o*-ICz-PI** at RT. (λ_{exc} = 355 nm). (d) Time-resolved emission spectra of ***o*-ICz-PI** in neat film at RT.

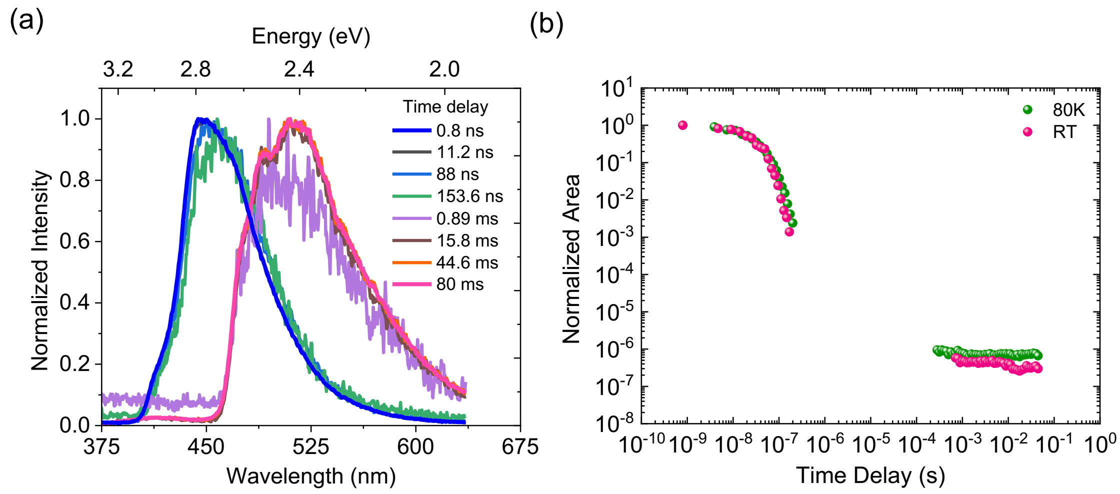

Supplementary Figure 9. Temperature dependent emission measurements of 1 wt.% zeonex films doped with ***o*-ICz-PI.** (a) Time-resolved emission spectra at 80 K and (b) time-resolved decay spectra at room temperature and 80 K for 1 wt.% zeonex films doped with ***o*-ICz-PI**. (λ_{exc} = 355 nm).

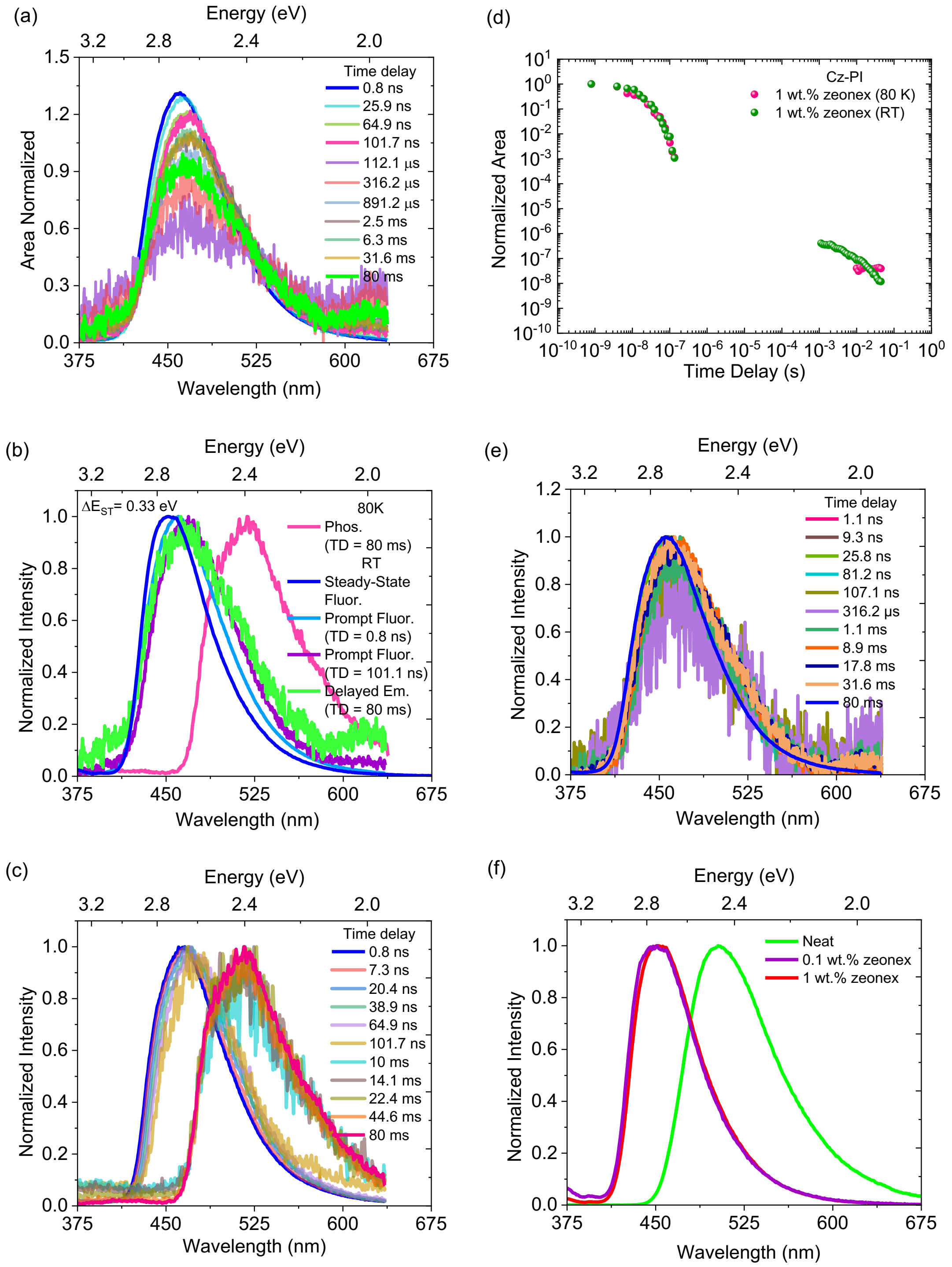

Supplementary Figure 10. Photophysical studies on the Cz-PI films. (a) Time-resolved emission spectra, (b) steady-state photoluminescence (room temperature) and phosphorescence (80 K, 80 ms delay) of **Cz-PI** doped in 1 wt.% zeonex films. (c) Time-resolved emission spectra at 80 K and (d) time-resolved decay spectra at room temperature and 80 K for 1 wt.% zeonex films doped with **Cz-PI**, showing clear thermal activation for delayed fluorescence at room temperature. (e) Time-resolved emission spectra of 0.1 wt.% zeonex film doped with **Cz-PI** at

RT. (f) Steady-state emission spectra at different doping concentrations in zeonex and neat film for **Cz-PI**. (λ_{exc} = 355 nm).

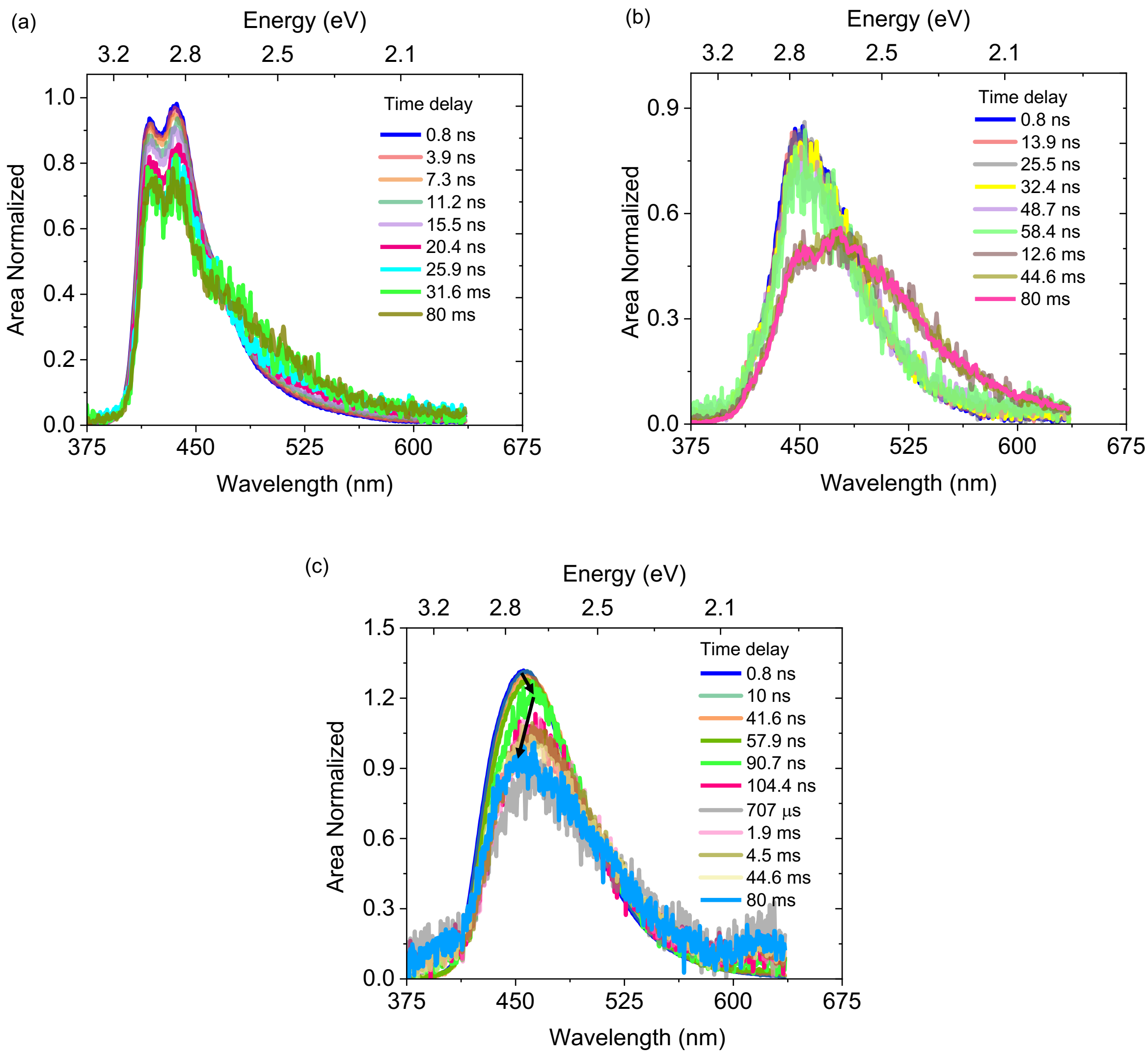

Supplementary Figure 11. Photophysical studies on the doped zeonex films. Time-resolved emission spectra of 0.1 wt.% zeonex film doped with (a) ***p*-ICz-PI**, (b) ***o*-ICz-PI** and (c) **Cz-PI** at RT. (λ_{exc} = 355 nm). The area normalized spectra show **Cz-PI** has a dispersion of CT-emission (black arrow) due to flexible N-C bonds typically seen in D-A TADF chromophores.[5] However, for the ICz-PI isomers the emission spectra show no such dispersion related effect (see Supplementary Fig. 6, 8 and 10e). Low doping concentration was chosen to eliminate any aggregation-related emission feature for this comparison.

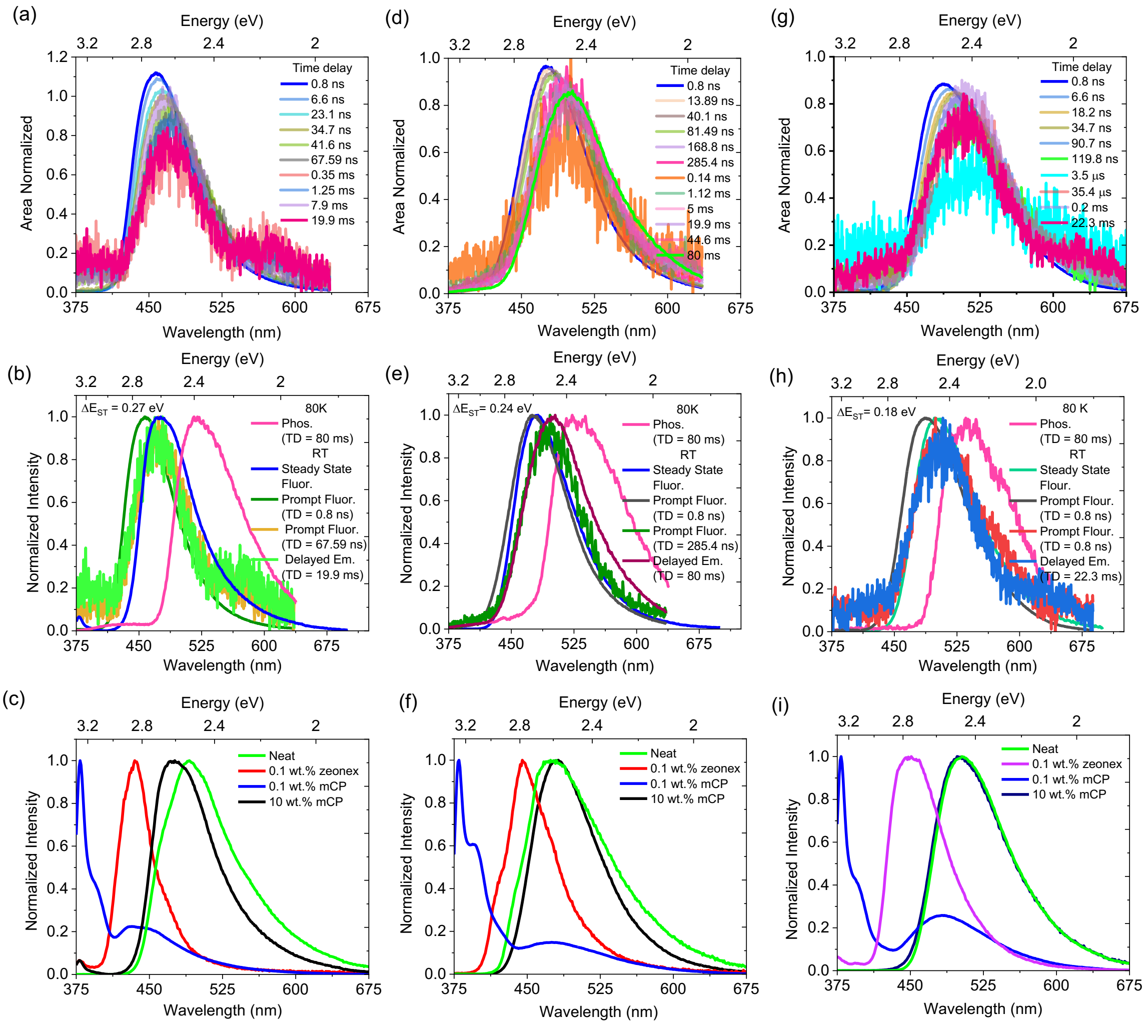

Supplementary Figure 12. Photophysical studies of the investigated molecules doped in mCP. Time-resolved emission spectra at room temperature for (a) ***p*-ICz-PI**, (d) ***o*-ICz-PI** and (g) **Cz-PI**. Steady-state photoluminescence (room temperature) and phosphorescence (80 K, 80 ms delay) of (b) ***p*-ICz-PI**, (e) ***o*-ICz-PI** and (h) **Cz-PI** doped in 10 wt.% mCP films. Steady-state emission spectra at different doping concentrations in mCP for (c) ***p*-ICz-PI**, (f) ***o*-ICz-PI** and (i) **Cz-PI** at room temperature. Neat and 0.1 wt.% zeonex films at low doping ratios are also shown to understand the extent of aggregation effects in mCP. (λ_{exc} = 355 nm).

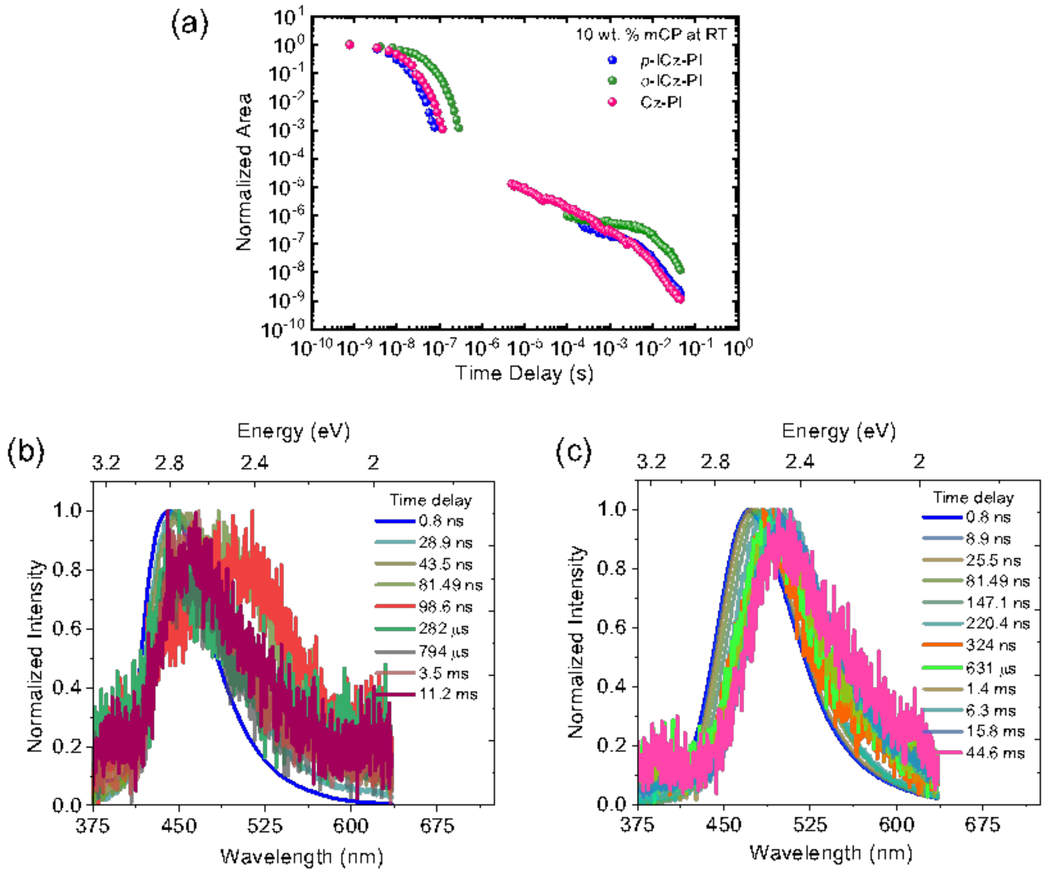

Supplementary Figure 13. Time-resolved emission studies of the investigated molecules doped in mCP at RT. Time-resolved emission spectra of 10 wt.% mCP films doped with ***p*-ICz-PI**, ***o*-ICz-PI** and **Cz-PI** at RT. (λ_{exc} = 355 nm). Time-resolved emission spectra of 0.1 wt.% mCP films doped with (b) ***p*-ICz-PI** and (c) ***o*-ICz-PI** at RT.

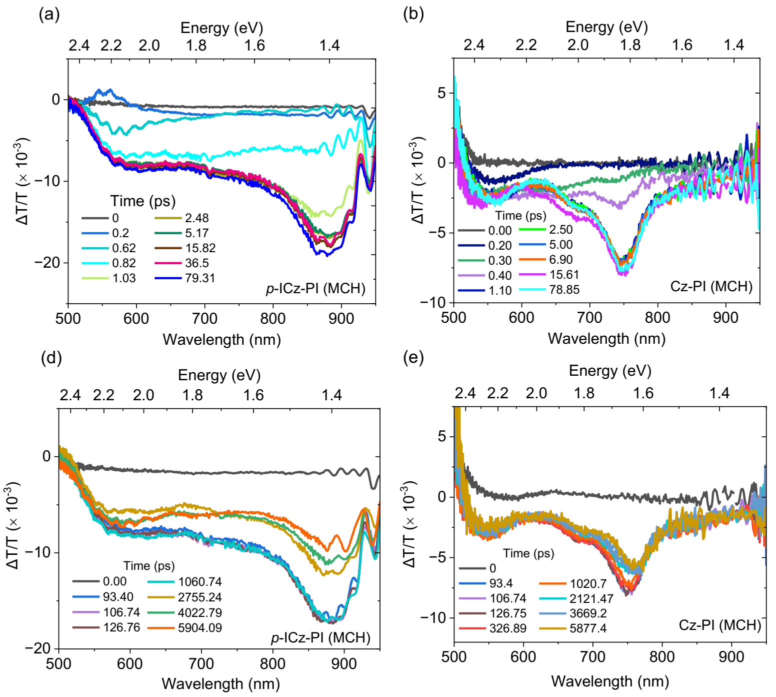

Supplementary Figure 14. Transient photoinduced absorption measurements of all investigated molecules in MCH. Excited state Absorption measurements in the 0-80 ps and 0-6 ns time windows for (a and d) ***p*-ICz-PI**, (b and e) **Cz-PI** and (c and f) ***o*-ICz-PI** in non-polar MCH solutions ([c] = 1 × 10^{-4} M, λ_{exc} = 343 nm).

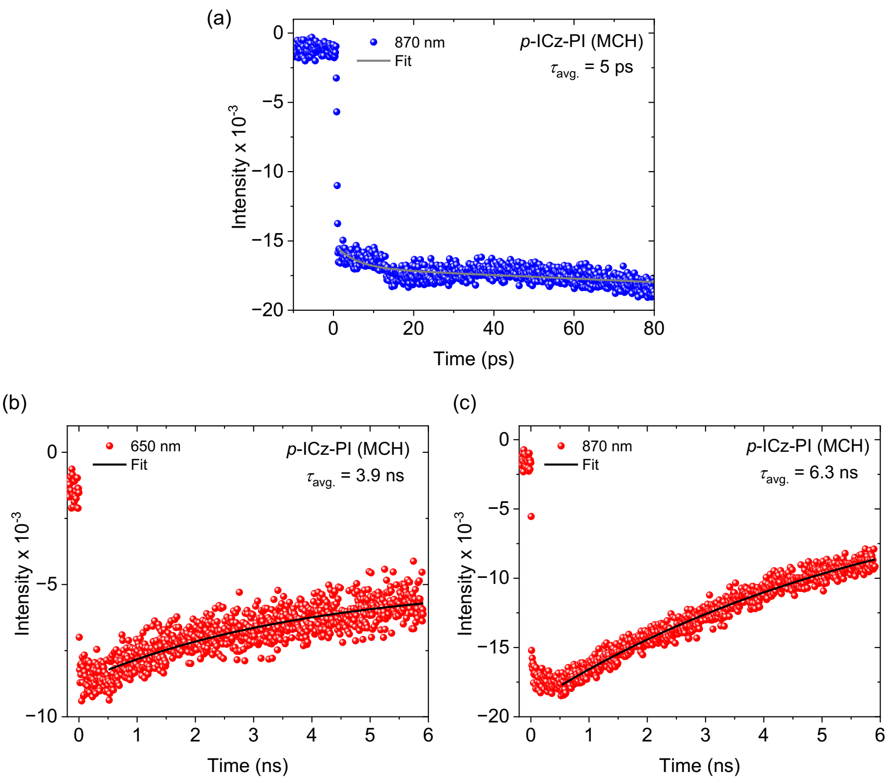

Supplementary Figure 15. Transient photoinduced absorption measurements of *p*-ICz-PI in MCH. Transient absorption decays and lifetimes of ***p*-ICz-PI** in MCH, monitoring the bands at (a) 870 nm in the early ps, (b) 650 nm and (c) 870 nm in the ns time scales. (λ_{exc} = 343 nm, [c] = 1 × 10^{-4} M).

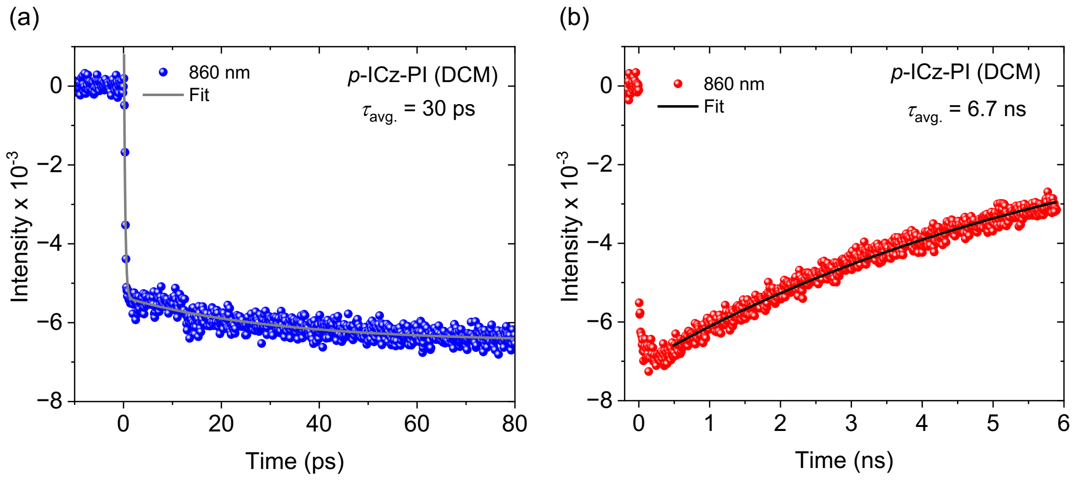

Supplementary Figure 16. Transient photoinduced absorption measurements of *p*-ICz-PI in DCM. Transient absorption decays and lifetimes of ***p*-ICz-PI** in DCM, monitoring the band at 860 nm in the (a) ps and (b) ns time scale. (λ_{exc} = 343 nm, [c] = 1 × 10^{-4} M).

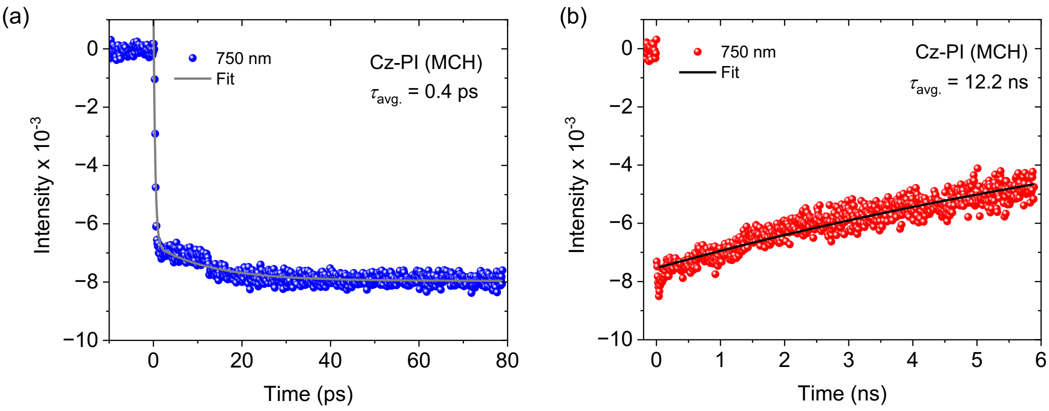

Supplementary Figure 17. Transient photoinduced absorption measurements of Cz-PI in MCH. Transient absorption decays and lifetimes of **Cz-PI** in MCH, monitoring the band at 750 nm in the (a) early ps and (b) in the ns time scales. (λ_{exc} = 343 nm, [c] = 1 × 10^{-4} M).

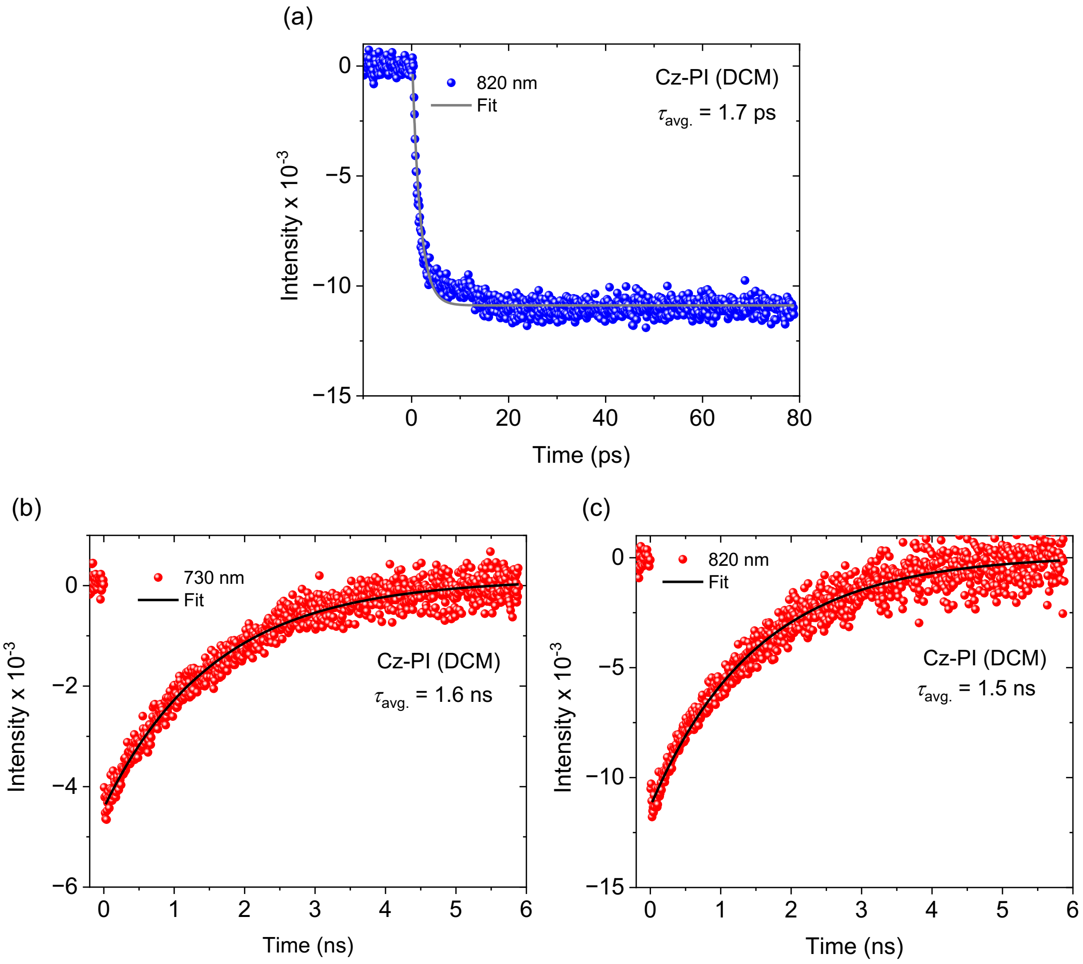

Supplementary Figure 18. Transient photoinduced absorption measurements of Cz-PI in DCM. Transient absorption decays and lifetimes of **Cz-PI** in DCM, monitoring the bands at

(a) 820 nm in the early ps, (b) 730 nm and (c) 820 nm in the ns time scales. (λ_{exc} = 343 nm, [c] = 1 × 10^{-4} M).

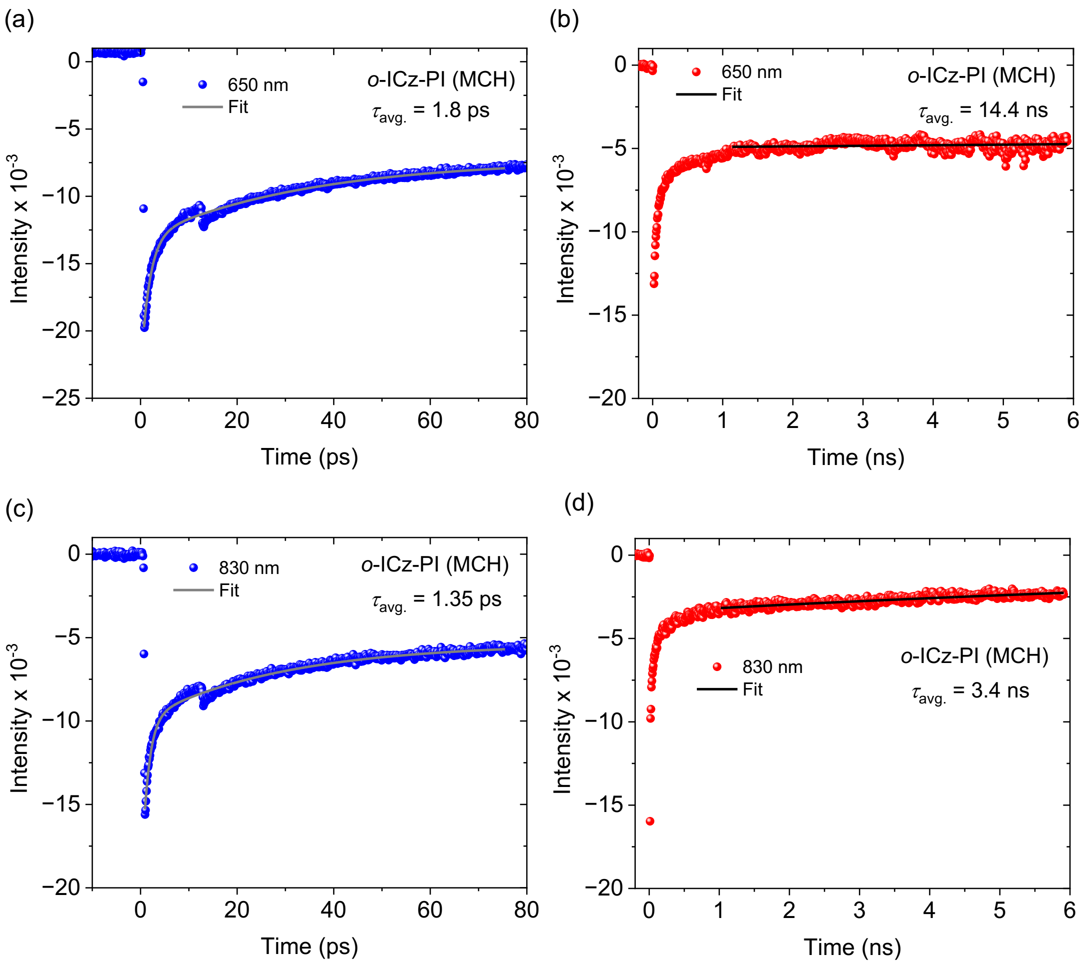

Supplementary Figure 19. Transient photoinduced absorption measurements of *o*-ICz-PI in MCH. Transient absorption decays and lifetimes of ***o*-ICz-PI** in MCH, monitoring the bands at (a) 650 nm and (c) 830 nm in the early ps, (b) 650 nm and (d) 830 nm in the ns time scales. (λ_{exc} = 343 nm, [c] = 1 × 10^{-4} M).

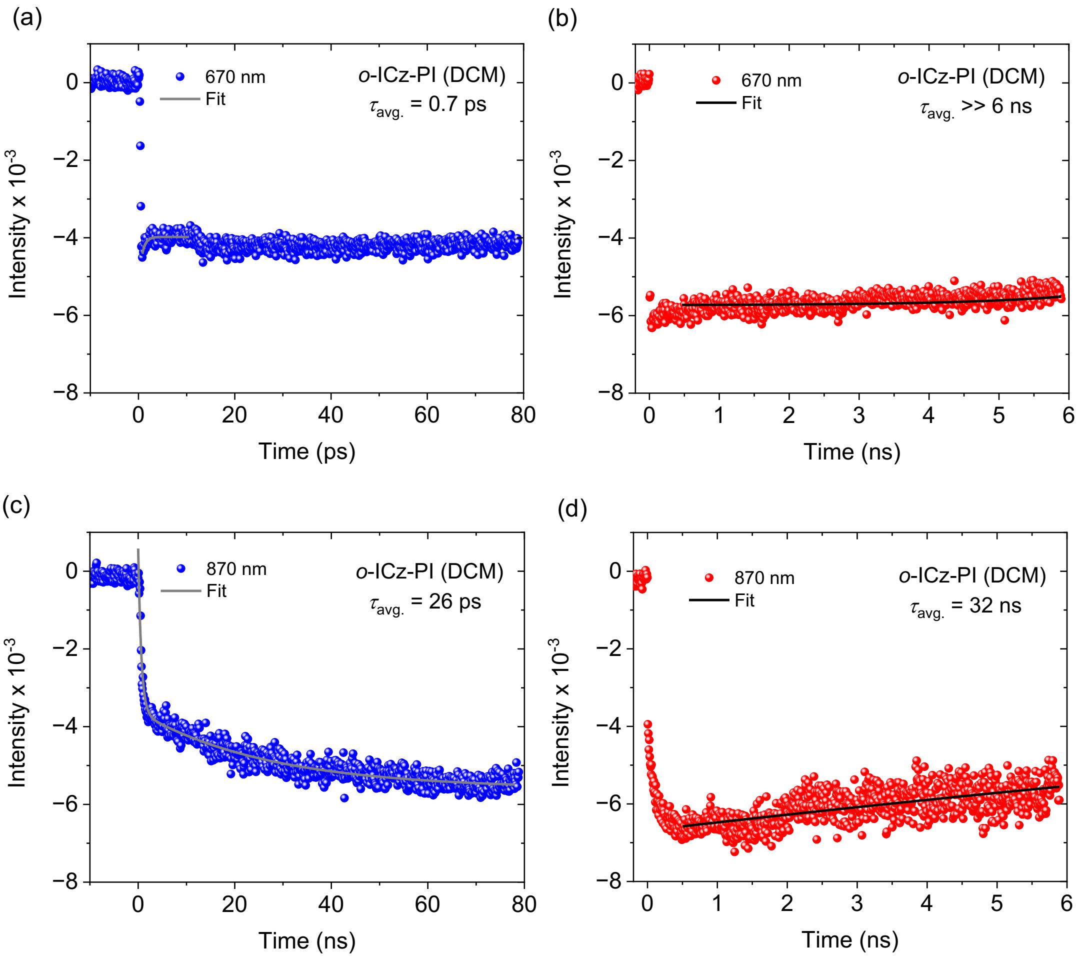

Supplementary Figure 20. Transient photoinduced absorption measurements of *o*-ICz-PI in DCM. Transient absorption decays and lifetimes of ***o*-ICz-PI** in DCM, monitoring the bands (a) 670 nm and (c) 870 nm in the early ps, (b) 670 nm and (d) 870 nm in the ns time scales. (λ_{exc} = 343 nm, [c] = 1 × 10^{-4} M).

Supplementary Table 3. Summary of the different monitored transient absorption bands studied in degassed MCH and DCM, in the ps and ns time range. ESA decay/grow-in times were calculated from simple exponential fits using the equation, $y = A_1 \times e^{-\frac{x}{t_1}} + y_0$.

λ_{exc} = 343 nm, [c] = 1 × 10^{-4} M. mon. is monitored.

Solvent	***p*-ICz-PI**			**Cz-PI**			***o*-ICz-PI**		
	$\lambda_{mon.}$ [nm]	Time[a] [ps]	Time [ns]	$\lambda_{mon.}$ [nm]	Time[a] [ps]	Time [ns]	$\lambda_{mon.}$ [nm]	Time[a] [ps]	Time [ns]
MCH	650	-	3.9				650	1.8	14.4
	870	5	6.3	750	0.40	12.2	830	1.35	3.4
DCM				730	-	1.6	670	0.7	>> 6
	860	30	6.7	820	1.7	1.5	870	26	32

[a]Fitted grow-in time.

5. Electronic Structure Simulations.

This section reports the electronic structure of the three studied molecules at the minimum energy of the ground state (GS), of S1 and T1 for the gas phase and different solvents. XYZ Cartesian coordinates files are available for download as an archive. Energies are given in eV relative to the ground state energy at the ground state minimum.

5.1. *p*-ICz-PI

5.1.1 Electronic structure – Gas Phase

State	f	Δ E / eV	State	f	Δ E / eV	State	f	Δ E / eV
S_0	—	0.00	S_0	—	0.28	S_0	—	0.25
S_1	0.167	3.42	S_1	0.083	3.20	S_1	0.072	3.31
S_2	0.072	3.69	S_2	0.104	3.75	S_2	0.150	3.74
S_3	0.055	3.92	S_3	0.098	3.91	S_3	0.059	3.86
S_4	0.000	4.02	S_4	0.000	4.05	S_4	0.000	4.00
S_5	0.171	4.19	S_5	0.243	4.25	S_5	0.242	4.34
S_6	0.056	4.38	S_6	0.087	4.55	S_6	0.004	4.48
S_7	0.001	4.55	S_7	0.048	4.57	S_7	0.062	4.56
S_8	0.625	4.56	S_8	0.040	4.65	S_8	0.092	4.67
S_9	0.101	4.61	S_9	0.268	4.75	S_9	0.723	4.85
S_{10}	0.099	4.73	S_{10}	0.461	4.90	S_{10}	0.260	4.92
T_1	—	2.88	T_1	—	2.75	T_1	—	2.66
T_2	—	2.99	T_2	—	3.02	T_2	—	3.07
T_3	—	3.14	T_3	—	3.26	T_3	—	3.27
T_4	—	3.52	T_4	—	3.68	T_4	—	3.63
T_5	—	3.63	T_5	—	3.68	T_5	—	3.69
T_6	—	3.79	T_6	—	3.82	T_6	—	3.79
T_7	—	3.81	T_7	—	3.89	T_7	—	3.97
T_8	—	3.98	T_8	—	4.09	T_8	—	4.04
T_9	—	4.03	T_9	—	4.18	T_9	—	4.11
T_{10}	—	4.14	T_{10}	—	4.22	T_{10}	—	4.18

Supplementary Table 4. Electronic structure of ***p*-ICz-PI** in gas phase at the ground state (left), S1 (centre) and T1 (right) minimum energy geometries. The associated differences of electronic density are reported in Supplementary Fig. 21, 22 and 23.

	SOC / cm-1	S1	S2	T1	T2	T3	T4	T5
GS	T1	0.022	0.037	---	---	---	---	---
	T2	0.036	0.055	0.244	---	---	---	---
	T3	0.073	0.054	0.093	0.073	---	---	---
	T4	0.118	0.091	2.120	0.118	0.091	---	---
	T5	5.033	2.929	55.496	5.033	2.929	2.075	---

	SOC / cm-1	S1	S2	T1	T2	T3	T4	T5
S1	T1	0.014	0.073	---	---	---	---	---
	T2	0.037	0.079	0.146	---	---	---	---
	T3	0.062	0.032	0.221	0.062	---	---	---
	T4	3.029	2.850	42.650	3.029	2.850	---	---
	T5	2.410	2.311	33.882	2.410	2.311	2.349	---

	SOC / cm-1	S1	S2	T1	T2	T3	T4	T5
T1	T1	0.068	0.163	---	---	---	---	---
	T2	0.085	0.064	0.321	---	---	---	---
	T3	0.051	0.060	0.149	0.051	---	---	---
	T4	4.636	4.913	53.577	4.636	4.913	---	---
	T5	0.044	0.143	0.792	0.044	0.143	0.121	---

Supplementary Table 5. Spin-orbit coupling matrix elements ***p*-ICz-PI** in gas phase at the ground state (top), S1 (middle) and T1 (bottom) minimum energy geometries.

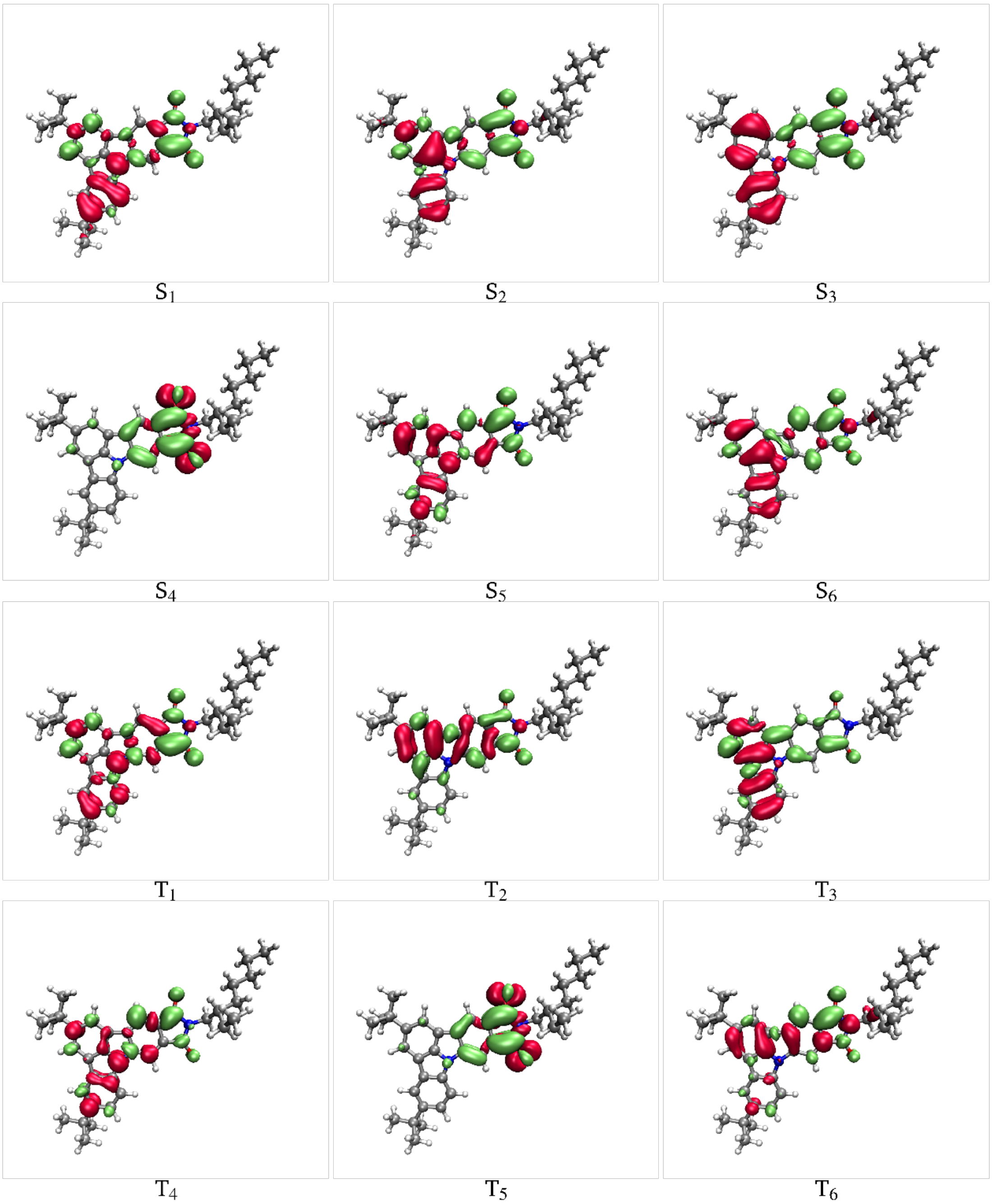

Supplementary Figure 21. Electron structure calculations of *p*-ICz-PI. Difference of electronic density associated to the transitions corresponding to the 6 lowest singlet and 6 lowest triplet states for ***p*-ICz-PI** at the GS geometry in gas phase. Red: Loss of electronic density. Green: Gain of electronic density.

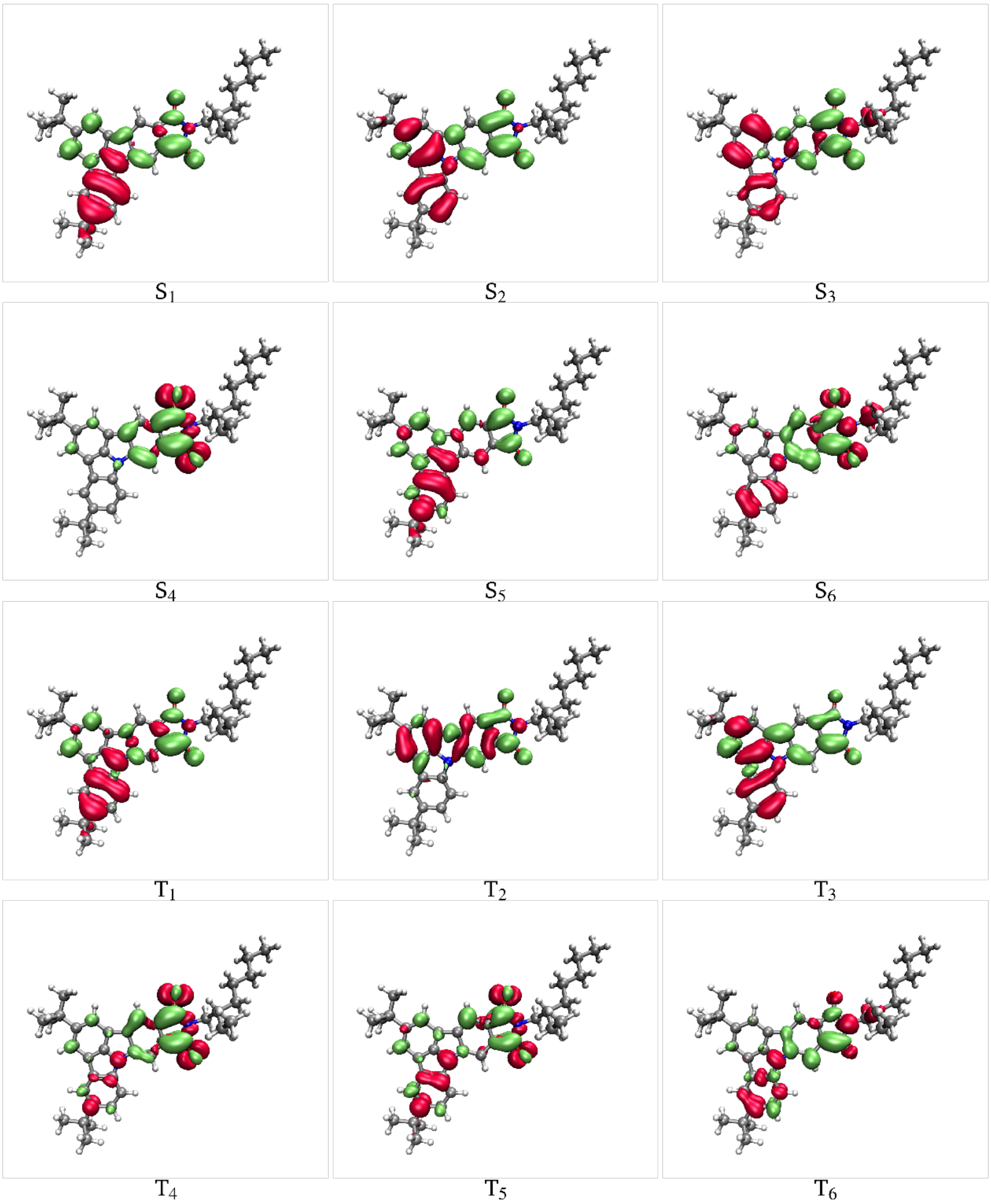

Supplementary Figure 22. Electron structure calculations of *p*-ICz-PI. Difference of electronic density associated to the transitions corresponding to the 6 lowest singlet and 6 lowest triplet states for ***p*-ICz-PI** at the S1 geometry in gas phase. Red: Loss of electronic density. Green: Gain of electronic density.

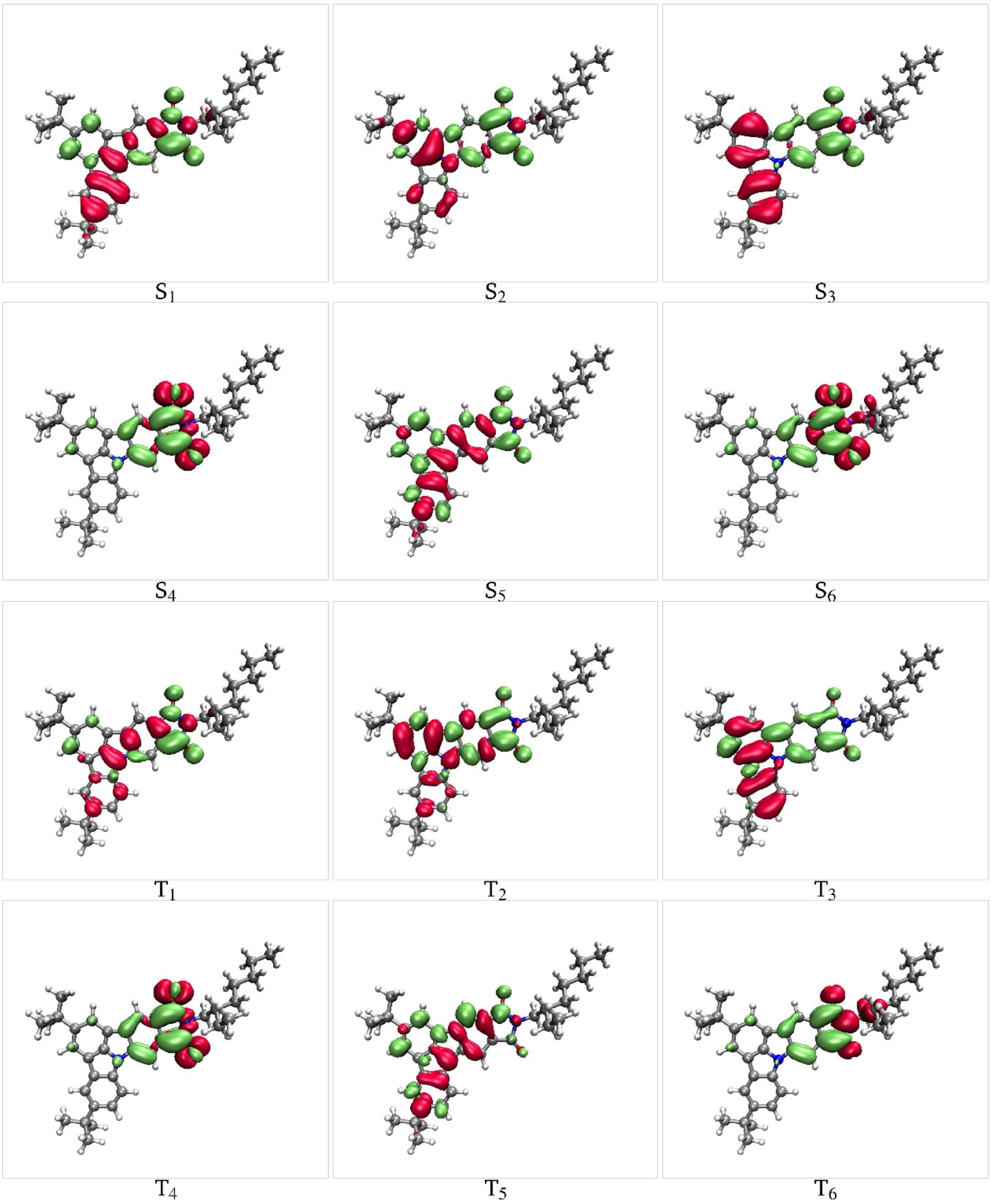

Supplementary Figure 23. Electron structure calculations of *p*-ICz-PI. Difference of electronic density associated to the transitions corresponding to the 6 lowest singlet and 6 lowest triplet states for ***p*-ICz-PI** at the T1 geometry in gas phase. Red: Loss of electronic density. Green: Gain of electronic density.

5.1.2 Electronic structure – Toluene

State	f	Δ E / eV	State	f	Δ E / eV	State	f	Δ E / eV
S_0	—	0.00	S_0	—	0.27	S_0	—	0.25
S_1	0.299	3.31	S_1	0.172	3.10	S_1	0.163	3.19
S_2	0.088	3.59	S_2	0.156	3.61	S_2	0.202	3.61
S_3	0.100	3.79	S_3	0.173	3.75	S_3	0.126	3.72
S_4	0.250	4.09	S_4	0.000	4.09	S_4	0.000	4.06
S_5	0.006	4.10	S_5	0.317	4.15	S_5	0.373	4.24
S_6	0.146	4.31	S_6	0.069	4.47	S_6	0.040	4.45
S_7	0.770	4.47	S_7	0.146	4.56	S_7	0.005	4.53
S_8	0.066	4.54	S_8	0.000	4.58	S_8	0.114	4.61
S_9	0.000	4.61	S_9	0.445	4.66	S_9	0.891	4.71
S_{10}	0.192	4.65	S_{10}	0.528	4.80	S_{10}	0.376	4.81
T_1	—	2.81	T_1	—	2.66	T_1	—	2.59
T_2	—	2.95	T_2	—	2.97	T_2	—	3.01
T_3	—	3.11	T_3	—	3.21	T_3	—	3.23
T_4	—	3.48	T_4	—	3.63	T_4	—	3.65
T_5	—	3.71	T_5	—	3.72	T_5	—	3.70
T_6	—	3.75	T_6	—	3.80	T_6	—	3.79
T_7	—	3.79	T_7	—	3.86	T_7	—	3.92
T_8	—	3.96	T_8	—	4.05	T_8	—	4.02
T_9	—	3.99	T_9	—	4.13	T_9	—	4.10
T_{10}	—	4.12	T_{10}	—	4.20	T_{10}	—	4.15

Supplementary Table 6. Electronic structure of ***p*-ICz-PI** in toluene at the ground state (left), S1 (centre) and T1 (right) minimum energy geometries.

5.1.3 Electronic structure – MCH

State	f	Δ E / eV	State	f	Δ E / eV	State	f	Δ E / eV
S_0	—	0.00	S_0	—	0.27	S_0	—	0.25
S_1	0.265	3.33	S_1	0.155	3.11	S_1	0.145	3.21
S_2	0.085	3.61	S_2	0.149	3.63	S_2	0.196	3.63
S_3	0.085	3.82	S_3	0.160	3.77	S_3	0.112	3.74
S_4	0.002	4.09	S_4	0.000	4.09	S_4	0.000	4.05
S_5	0.234	4.11	S_5	0.310	4.16	S_5	0.357	4.25
S_6	0.120	4.32	S_6	0.080	4.48	S_6	0.042	4.47
S_7	0.744	4.49	S_7	0.067	4.58	S_7	0.007	4.53
S_8	0.067	4.56	S_8	0.059	4.58	S_8	0.109	4.62
S_9	0.000	4.60	S_9	0.415	4.67	S_9	0.869	4.73
S_{10}	0.171	4.66	S_{10}	0.522	4.82	S_{10}	0.357	4.83
T_1	—	2.82	T_1	—	2.68	T_1	—	2.60
T_2	—	2.96	T_2	—	2.98	T_2	—	3.02
T_3	—	3.12	T_3	—	3.22	T_3	—	3.23
T_4	—	3.48	T_4	—	3.64	T_4	—	3.66
T_5	—	3.70	T_5	—	3.72	T_5	—	3.69
T_6	—	3.75	T_6	—	3.81	T_6	—	3.79
T_7	—	3.79	T_7	—	3.87	T_7	—	3.93
T_8	—	3.97	T_8	—	4.05	T_8	—	4.02
T_9	—	3.99	T_9	—	4.14	T_9	—	4.11
T_{10}	—	4.12	T_{10}	—	4.20	T_{10}	—	4.15

Supplementary Table 7. Electronic structure of ***p*-ICz-PI** in methylcyclohexane at the ground state (left), S1 (centre) and T1 (right) minimum energy geometries.

5.1.4 Electronic structure – Dichloromethane

State	f	Δ E / eV	State	f	Δ E / eV	State	f	Δ E / eV
S_0	—	0.00	S_0	—	0.26	S_0	—	0.25
S_1	0.302	3.26	S_1	0.268	3.01	S_1	0.257	3.08
S_2	0.076	3.53	S_2	0.179	3.50	S_2	0.212	3.51
S_3	0.098	3.72	S_3	0.233	3.64	S_3	0.206	3.61
S_4	0.219	4.05	S_4	0.326	4.08	S_4	0.000	4.10
S_5	0.000	4.15	S_5	0.000	4.11	S_5	0.421	4.17
S_6	0.147	4.28	S_6	0.027	4.40	S_6	0.032	4.38
S_7	0.722	4.47	S_7	0.223	4.50	S_7	0.007	4.55
S_8	0.035	4.51	S_8	0.045	4.58	S_8	0.169	4.55
S_9	0.241	4.61	S_9	0.555	4.59	S_9	0.956	4.63
S_{10}	0.001	4.64	S_{10}	0.543	4.73	S_{10}	0.448	4.74
T_1	—	2.75	T_1	—	2.59	T_1	—	2.53
T_2	—	2.93	T_2	—	2.93	T_2	—	2.96
T_3	—	3.09	T_3	—	3.16	T_3	—	3.19
T_4	—	3.45	T_4	—	3.60	T_4	—	3.63
T_5	—	3.72	T_5	—	3.74	T_5	—	3.74
T_6	—	3.76	T_6	—	3.79	T_6	—	3.79
T_7	—	3.79	T_7	—	3.84	T_7	—	3.89
T_8	—	3.91	T_8	—	4.02	T_8	—	4.01
T_9	—	4.00	T_9	—	4.07	T_9	—	4.04
T_{10}	—	4.11	T_{10}	—	4.17	T_{10}	—	4.18

Supplementary Table 8. Electronic structure of ***p*-ICz-PI** in dichloromethane at the ground state (left), S1 (centre) and T1 (right) minimum energy geometries. The associated differences of electronic density are reported in Supplementary Fig. 24, 25 and 26.

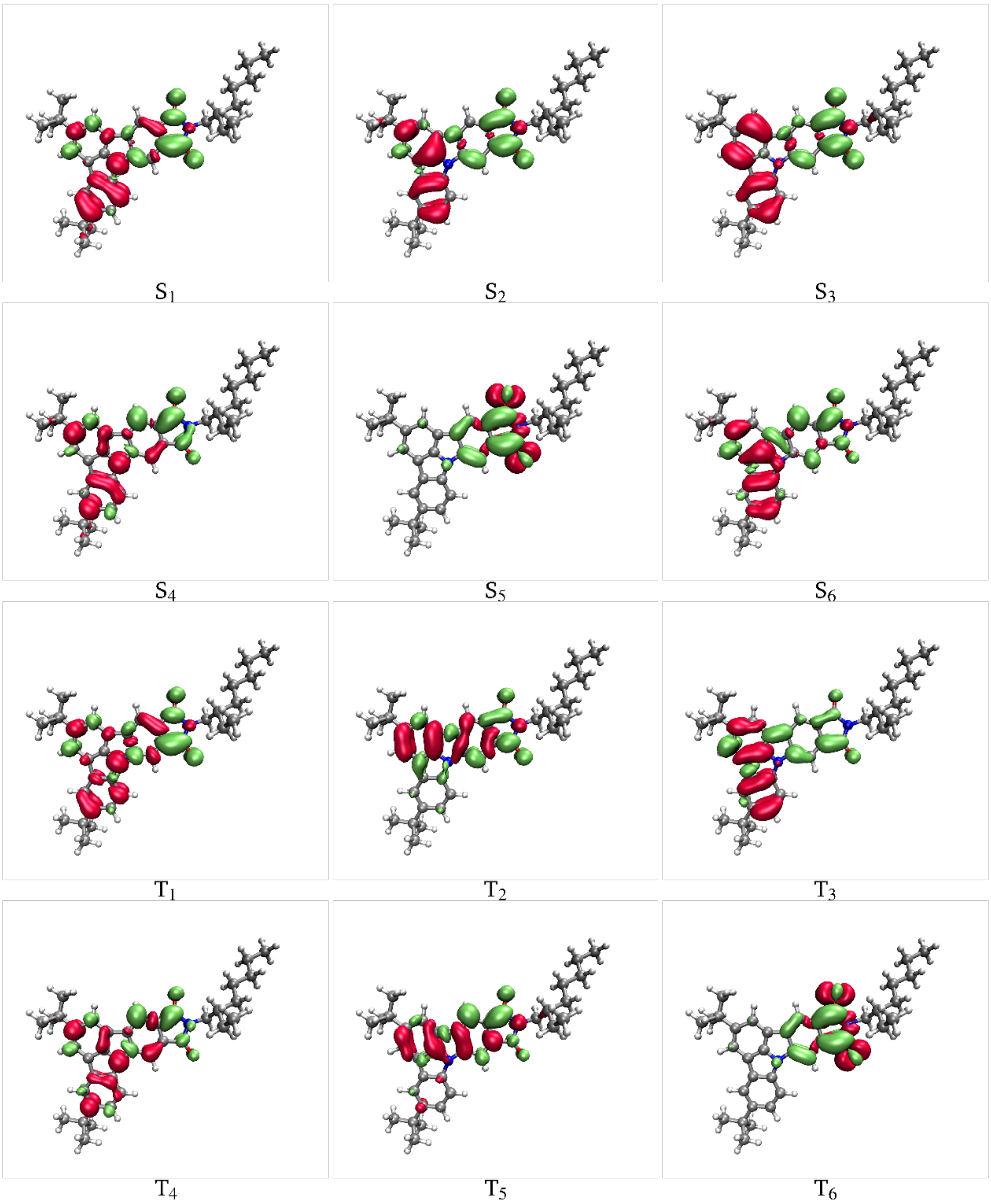

Supplementary Figure 24. Electron structure calculations of *p*-ICz-PI. Difference of electronic density associated to the transitions corresponding to the 6 lowest singlet and 6 lowest triplet states for ***p*-ICz-PI** at the GS geometry in dichloromethane. Red: Loss of electronic density. Green: Gain of electronic density.

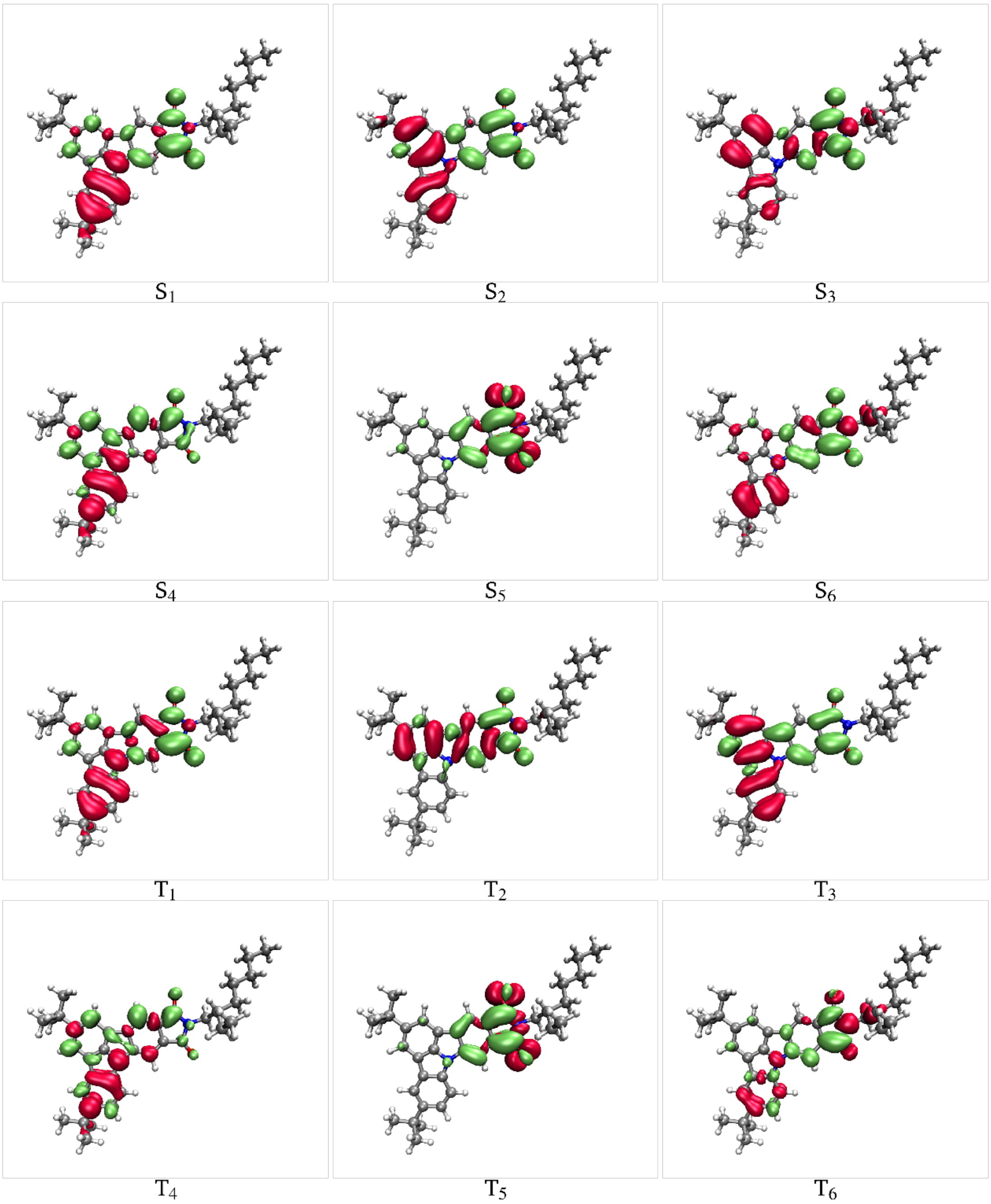

Supplementary Figure 25. Electron structure calculations of *p*-ICz-PI. Difference of electronic density associated to the transitions corresponding to the 6 lowest singlet and 6 lowest triplet states for ***p*-ICz-PI** at the S1 geometry in dichloromethane. Red: Loss of electronic density. Green: Gain of electronic density.

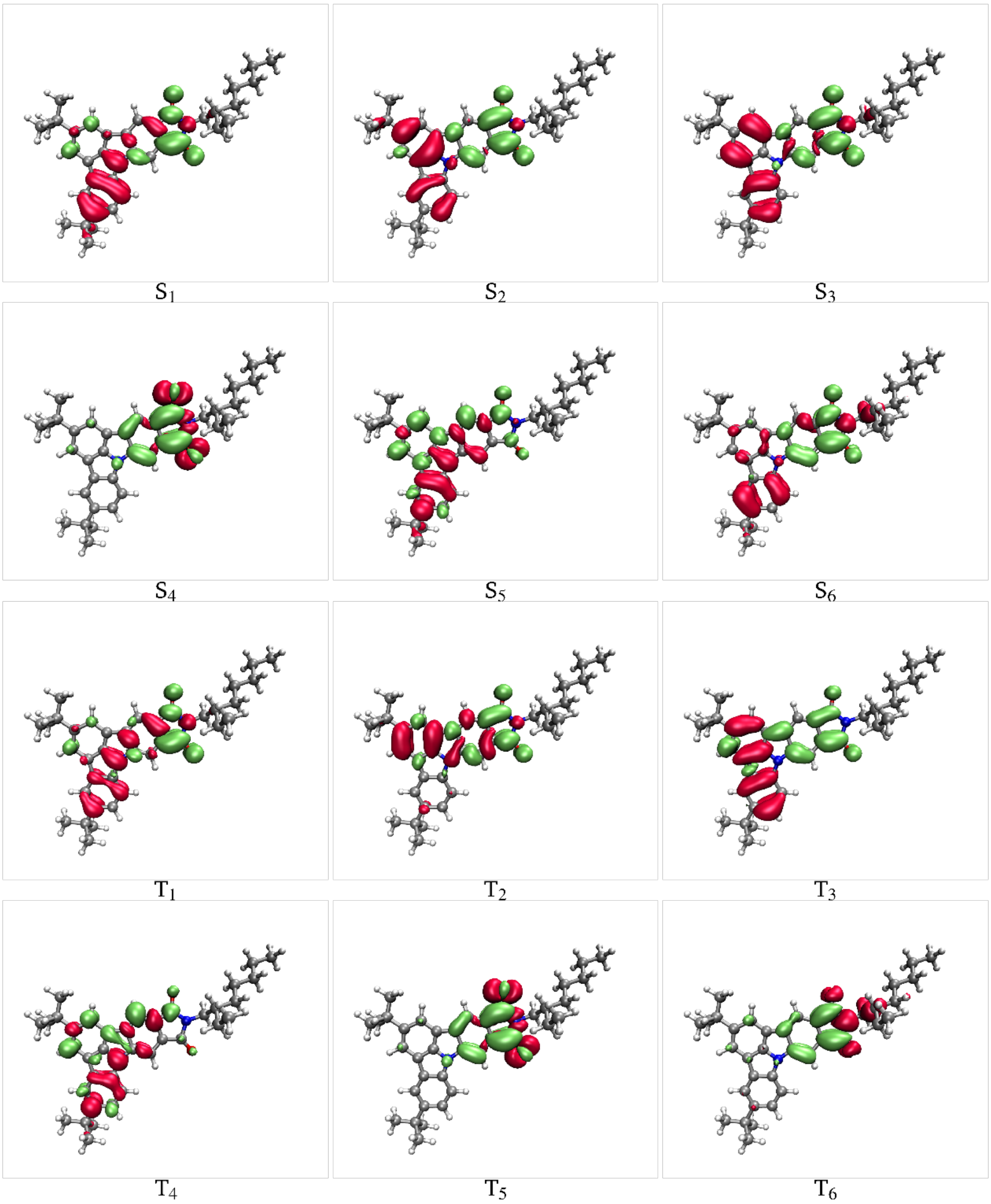

Supplementary Figure 26. Electron structure calculations of *p*-ICz-PI. Difference of electronic density associated to the transitions corresponding to the 6 lowest singlet and 6 lowest triplet states for ***p*-ICz-PI** at the T1 geometry in dichloromethane. Red: Loss of electronic density. Green: Gain of electronic density.

5.2. *o*-ICz-PI

5.2.1 Electronic structure – Gas Phase

State	f	Δ E / eV	State	f	Δ E / eV	State	f	Δ E / eV
S_0	—	0.00	S_0	—	0.32	S_0	—	0.24
S_1	0.010	3.28	S_1	0.005	3.00	S_1	0.019	3.10
S_2	0.023	3.64	S_2	0.007	3.61	S_2	0.012	3.61
S_3	0.045	3.76	S_3	0.011	3.87	S_3	0.006	3.80
S_4	0.002	3.99	S_4	0.000	3.99	S_4	0.000	3.92
S_5	0.596	4.09	S_5	0.431	4.16	S_5	0.185	4.09
S_6	0.045	4.15	S_6	0.340	4.31	S_6	0.564	4.28
S_7	0.033	4.48	S_7	0.003	4.45	S_7	0.020	4.39
S_8	0.008	4.53	S_8	0.048	4.49	S_8	0.044	4.43
S_9	0.356	4.62	S_9	0.019	4.84	S_9	0.157	4.86
S_{10}	0.217	4.72	S_{10}	0.064	4.90	S_{10}	0.168	4.93
T_1	—	2.81	T_1	—	2.66	T_1	—	2.58
T_2	—	3.06	T_2	—	3.25	T_2	—	3.22
T_3	—	3.17	T_3	—	3.31	T_3	—	3.26
T_4	—	3.35	T_4	—	3.46	T_4	—	3.48
T_5	—	3.55	T_5	—	3.60	T_5	—	3.54
T_6	—	3.59	T_6	—	3.71	T_6	—	3.63
T_7	—	3.66	T_7	—	3.76	T_7	—	3.69
T_8	—	3.83	T_8	—	3.92	T_8	—	3.85
T_9	—	3.91	T_9	—	4.05	T_9	—	3.96
T_{10}	—	4.11	T_{10}	—	4.07	T_{10}	—	4.00

Supplementary Table 9. Electronic structure of ***o*-ICz-PI** in gas phase at the ground state (left), S1 (centre) and T1 (right) minimum energy geometries. The associated differences of electronic density are reported in Supplementary Fig. 27, 28 and 29.

	SOC / cm^{-1}	S1	S2	T1	T2	T3	T4	T5
GS	T1	0.032	0.083	---	---	---	---	---
	T2	0.032	0.032	0.304	---	---	---	---
	T3	0.099	0.022	0.566	0.099	---	---	---
	T4	0.087	0.050	1.258	0.087	0.050	---	---
	T5	0.404	0.237	6.381	0.404	0.237	0.189	---

	SOC / cm-1	S1	S2	T1	T2	T3	T4	T5
S1	T1	0.040	0.111	---	---	---	---	---
	T2	0.051	0.037	0.238	---	---	---	---
	T3	0.086	0.082	0.723	0.086	---	---	---
	T4	0.112	0.051	0.266	0.112	0.051	---	---
	T5	2.486	1.651	55.464	2.486	1.651	5.093	---

	SOC / cm-1	S1	S2	T1	T2	T3	T4	T5
T1	T1	0.079	0.108	---	---	---	---	---
	T2	0.130	0.086	0.727	---	---	---	---
	T3	0.036	0.037	0.215	0.036	---	---	---
	T4	0.124	0.051	2.089	0.124	0.051	---	---
	T5	3.466	1.593	54.019	3.466	1.593	5.260	---

Supplementary Table 10. Spin-orbit coupling matrix elements ***o*-ICz-PI** in gas phase at the ground state (top), S1 (middle) and T1 (bottom) minimum energy geometries.

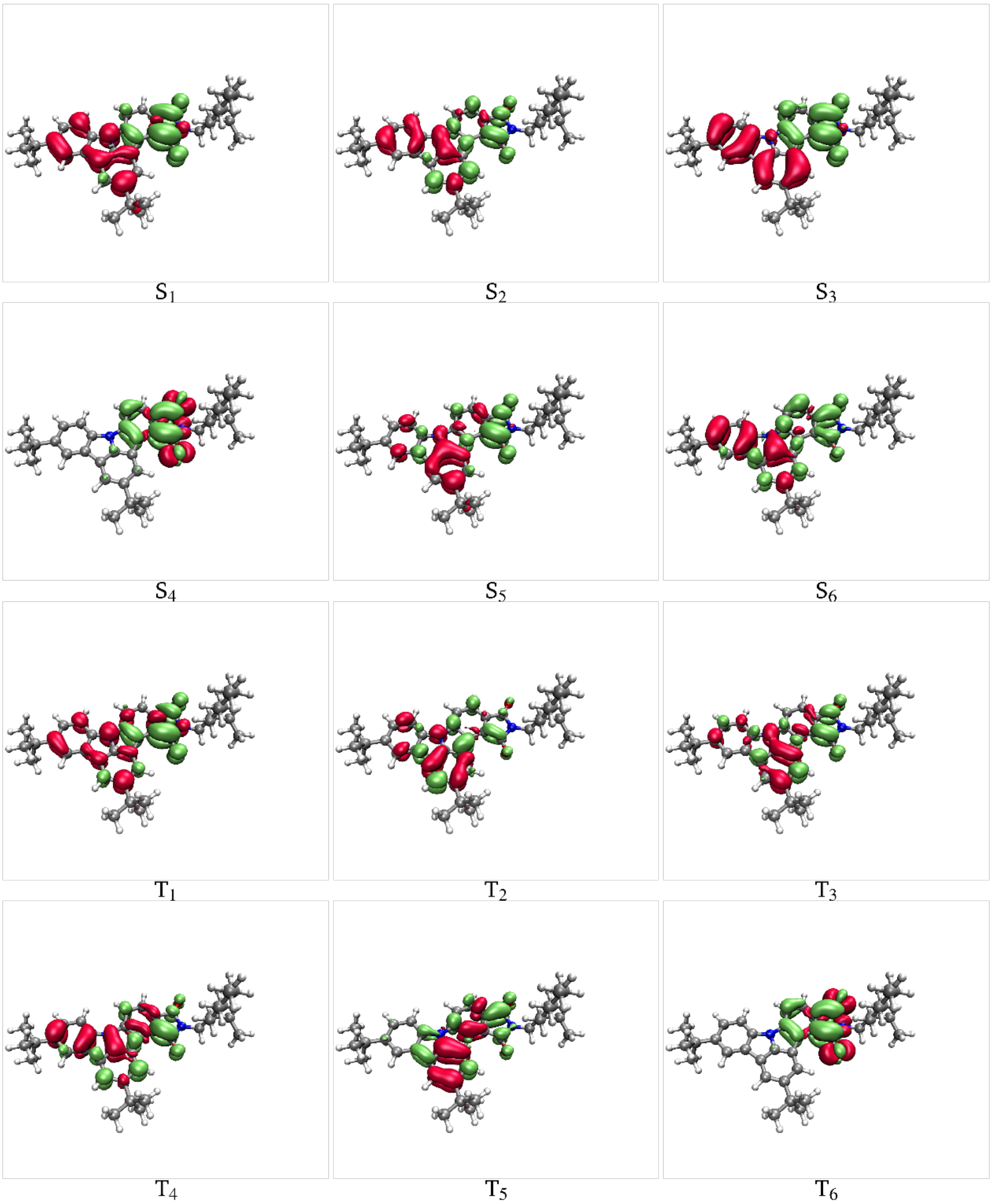

Supplementary Figure 27. Electron structure calculations of *o*-ICz-PI. Difference of electronic density associated to the transitions corresponding to the 6 lowest singlet and 6 lowest triplet states for ***o*-ICz-PI** at the GS geometry in gas phase. Red: Loss of electronic density. Green: Gain of electronic density.

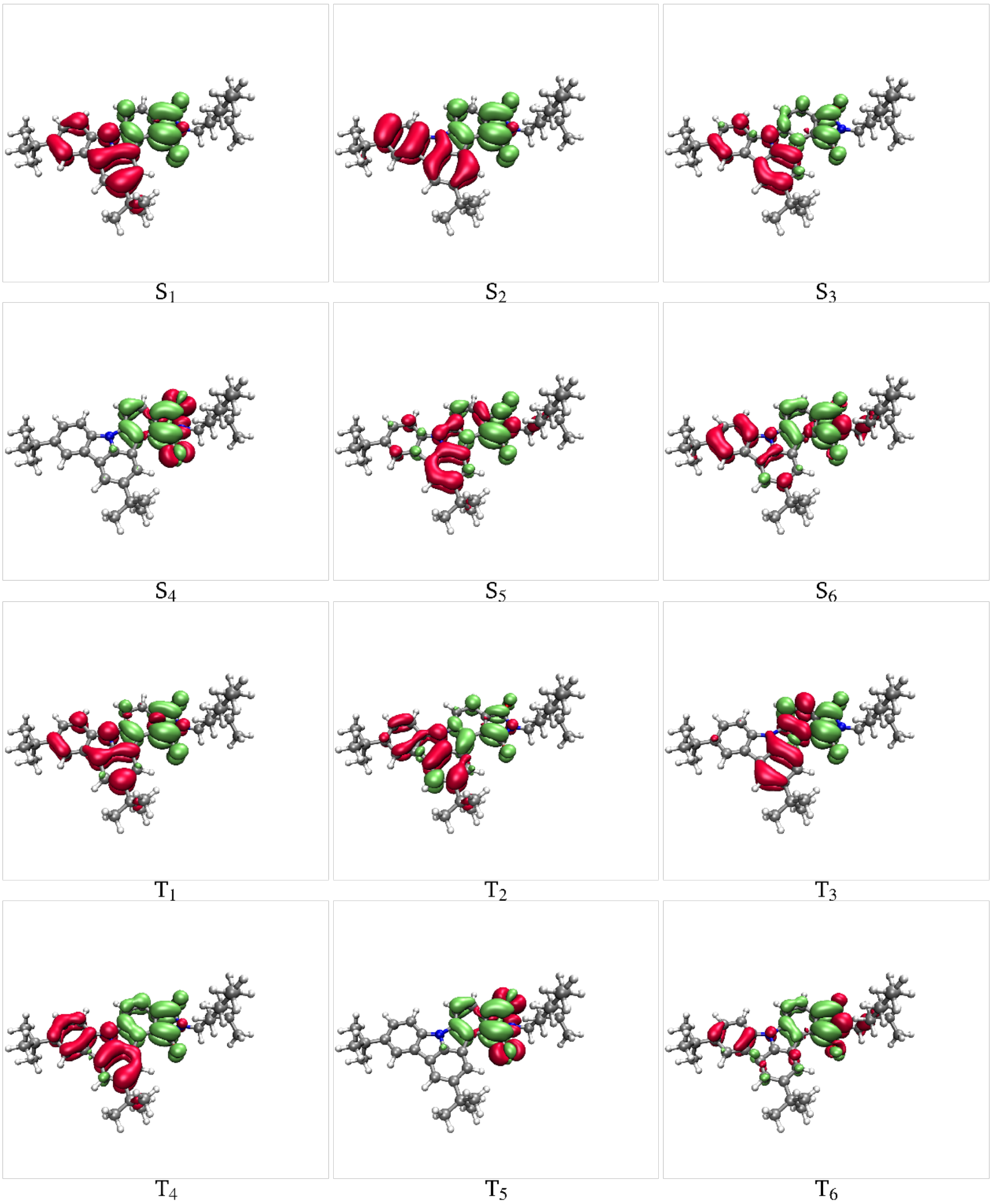

Supplementary Figure 28. Electron structure calculations of *o*-ICz-PI. Difference of electronic density associated to the transitions corresponding to the 6 lowest singlet and 6 lowest triplet states for ***o*-ICz-PI** at the S1 geometry in gas phase. Red: Loss of electronic density. Green: Gain of electronic density.

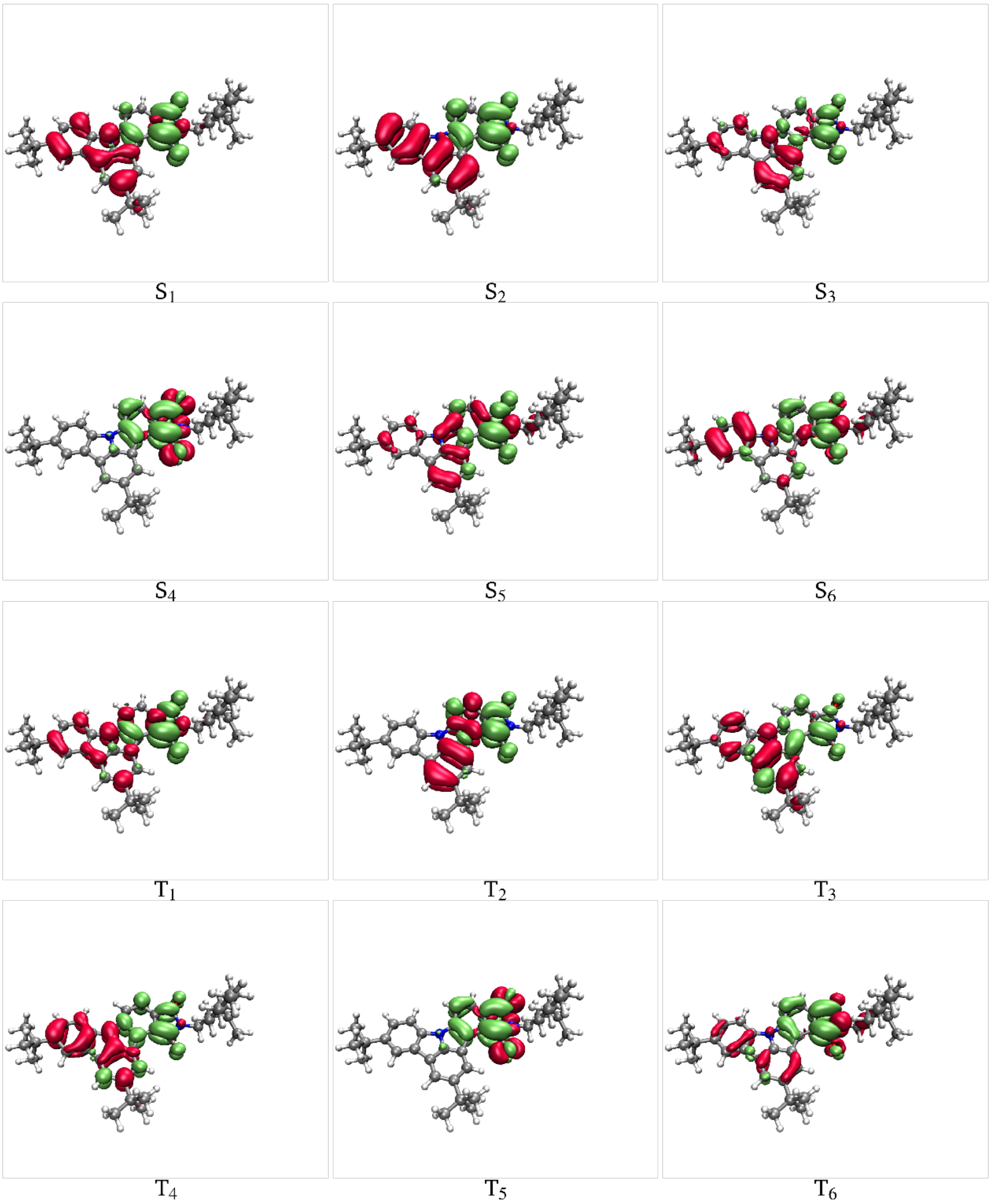

Supplementary Figure 29. Electron structure calculations of *o*-ICz-PI. Difference of electronic density associated to the transitions corresponding to the 6 lowest singlet and 6 lowest triplet states for ***o*-ICz-PI** at the T1 geometry in gas phase. Red: Loss of electronic density. Green: Gain of electronic density.

5.2.2 Electronic structure – Toluene

State	f	Δ E / eV	State	f	Δ E / eV	State	f	Δ E / eV
S_0	—	0.00	S_0	—	0.30	S_0	—	0.24
S_1	0.021	3.18	S_1	0.010	2.90	S_1	0.037	2.99
S_2	0.020	3.59	S_2	0.021	3.52	S_2	0.027	3.52
S_3	0.102	3.67	S_3	0.005	3.79	S_3	0.011	3.74
S_4	0.923	3.95	S_4	0.635	4.00	S_4	0.051	3.97
S_5	0.001	4.05	S_5	0.130	4.01	S_5	0.418	3.98
S_6	0.034	4.10	S_6	0.363	4.18	S_6	0.636	4.16
S_7	0.092	4.41	S_7	0.037	4.42	S_7	0.078	4.39
S_8	0.408	4.53	S_8	0.001	4.45	S_8	0.002	4.42
S_9	0.001	4.55	S_9	0.028	4.78	S_9	0.010	4.80
S_{10}	0.273	4.69	S_{10}	0.170	4.84	S_{10}	0.498	4.85
T_1	—	2.74	T_1	—	2.57	T_1	—	2.50
T_2	—	3.05	T_2	—	3.20	T_2	—	3.16
T_3	—	3.14	T_3	—	3.22	T_3	—	3.23
T_4	—	3.31	T_4	—	3.41	T_4	—	3.44
T_5	—	3.53	T_5	—	3.62	T_5	—	3.60
T_6	—	3.60	T_6	—	3.67	T_6	—	3.63
T_7	—	3.66	T_7	—	3.72	T_7	—	3.66
T_8	—	3.82	T_8	—	3.87	T_8	—	3.83
T_9	—	3.91	T_9	—	4.03	T_9	—	3.96
T_{10}	—	4.14	T_{10}	—	4.07	T_{10}	—	4.05

Supplementary Table 11. Electronic structure of ***o*-ICz-PI** at the GS geometry in toluene at the ground state (left), S1 (centre) and T1 (right) minimum energy geometries.

5.2.3 Electronic structure – MCH

State	f	Δ E / eV	State	f	Δ E / eV	State	f	Δ E / eV
S_0	—	0.00	S_0	—	0.30	S_0	—	0.24
S_1	0.018	3.19	S_1	0.009	2.92	S_1	0.034	3.01
S_2	0.020	3.60	S_2	0.018	3.53	S_2	0.024	3.54
S_3	0.083	3.69	S_3	0.005	3.80	S_3	0.008	3.75
S_4	0.855	3.98	S_4	0.001	4.00	S_4	0.001	3.97
S_5	0.003	4.04	S_5	0.707	4.03	S_5	0.413	4.00
S_6	0.028	4.11	S_6	0.368	4.20	S_6	0.642	4.18
S_7	0.082	4.42	S_7	0.035	4.43	S_7	0.072	4.39
S_8	0.035	4.55	S_8	0.003	4.45	S_8	0.002	4.42
S_9	0.359	4.55	S_9	0.015	4.79	S_9	0.031	4.81
S_{10}	0.253	4.70	S_{10}	0.162	4.84	S_{10}	0.468	4.87
T_1	—	2.75	T_1	—	2.58	T_1	—	2.51
T_2	—	3.05	T_2	—	3.21	T_2	—	3.17
T_3	—	3.14	T_3	—	3.23	T_3	—	3.23
T_4	—	3.32	T_4	—	3.42	T_4	—	3.45
T_5	—	3.53	T_5	—	3.62	T_5	—	3.59
T_6	—	3.61	T_6	—	3.67	T_6	—	3.63
T_7	—	3.65	T_7	—	3.73	T_7	—	3.67
T_8	—	3.82	T_8	—	3.88	T_8	—	3.84
T_9	—	3.91	T_9	—	4.03	T_9	—	3.96
T_{10}	—	4.14	T_{10}	—	4.07	T_{10}	—	4.04

Supplementary Table 12. Electronic structure of ***o*-ICz-PI** at the GS geometry in methylcyclohexane at the ground state (left), S1 (centre) and T1 (right) minimum energy geometries.

5.2.4 Electronic structure – Dichloromethane

State	f	Δ E / eV	State	f	Δ E / eV	State	f	Δ E / eV
S_0	—	0.00	S_0	—	0.28	S_0	—	0.25
S_1	0.023	3.11	S_1	0.017	2.83	S_1	0.055	2.90
S_2	0.003	3.55	S_2	0.041	3.44	S_2	0.045	3.45
S_3	0.093	3.63	S_3	0.028	3.73	S_3	0.045	3.70
S_4	0.902	3.93	S_4	0.985	3.89	S_4	0.713	3.90
S_5	0.043	4.09	S_5	0.006	4.03	S_5	0.007	4.01
S_6	0.001	4.09	S_6	0.315	4.10	S_6	0.552	4.07
S_7	0.097	4.36	S_7	0.053	4.38	S_7	0.112	4.36
S_8	0.374	4.54	S_8	0.000	4.46	S_8	0.001	4.45
S_9	0.001	4.58	S_9	0.090	4.70	S_9	0.055	4.73
S_{10}	0.251	4.70	S_{10}	0.206	4.82	S_{10}	0.491	4.81
T_1	—	2.68	T_1	—	2.49	T_1	—	2.44
T_2	—	3.04	T_2	—	3.13	T_2	—	3.12
T_3	—	3.11	T_3	—	3.18	T_3	—	3.20
T_4	—	3.28	T_4	—	3.38	T_4	—	3.41
T_5	—	3.52	T_5	—	3.63	T_5	—	3.62
T_6	—	3.56	T_6	—	3.65	T_6	—	3.64
T_7	—	3.70	T_7	—	3.69	T_7	—	3.64
T_8	—	3.81	T_8	—	3.84	T_8	—	3.83
T_9	—	3.91	T_9	—	4.00	T_9	—	3.95
T_{10}	—	4.13	T_{10}	—	4.09	T_{10}	—	4.08

Supplementary Table 13. Electronic structure of ***o*-ICz-PI** at the GS geometry in dichloromethane at the ground state (left), S1 (centre) and T1 (right) minimum energy geometries. The associated differences of electronic density are reported in Figure 30, 31 and 32.

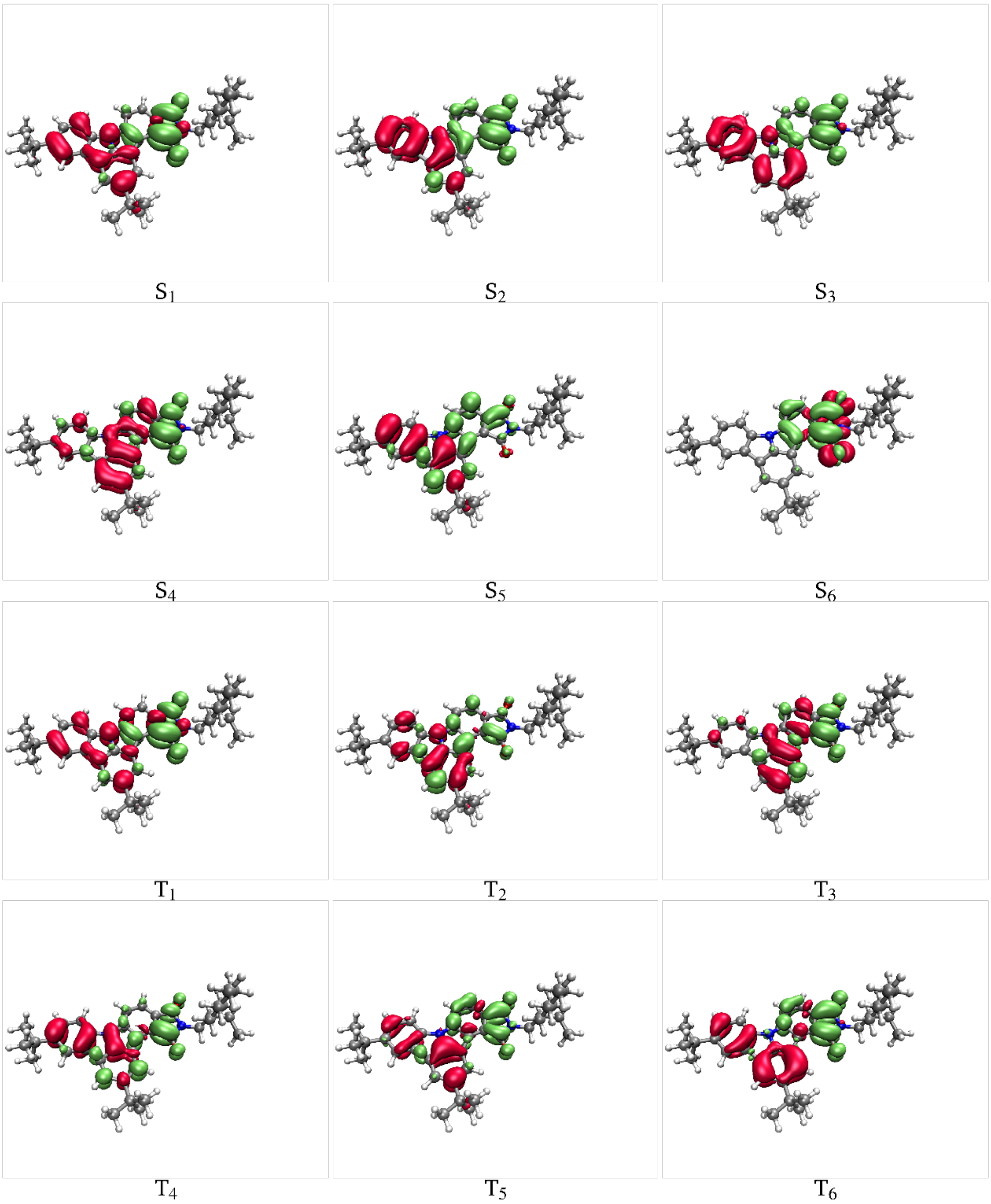

Supplementary Figure 30. Electron structure calculations of *o*-ICz-PI. Difference of electronic density associated to the transitions corresponding to the 6 lowest singlet and 6 lowest triplet states for ***o*-ICz-PI** at the GS geometry in dichloromethane. Red: Loss of electronic density. Green: Gain of electronic density.

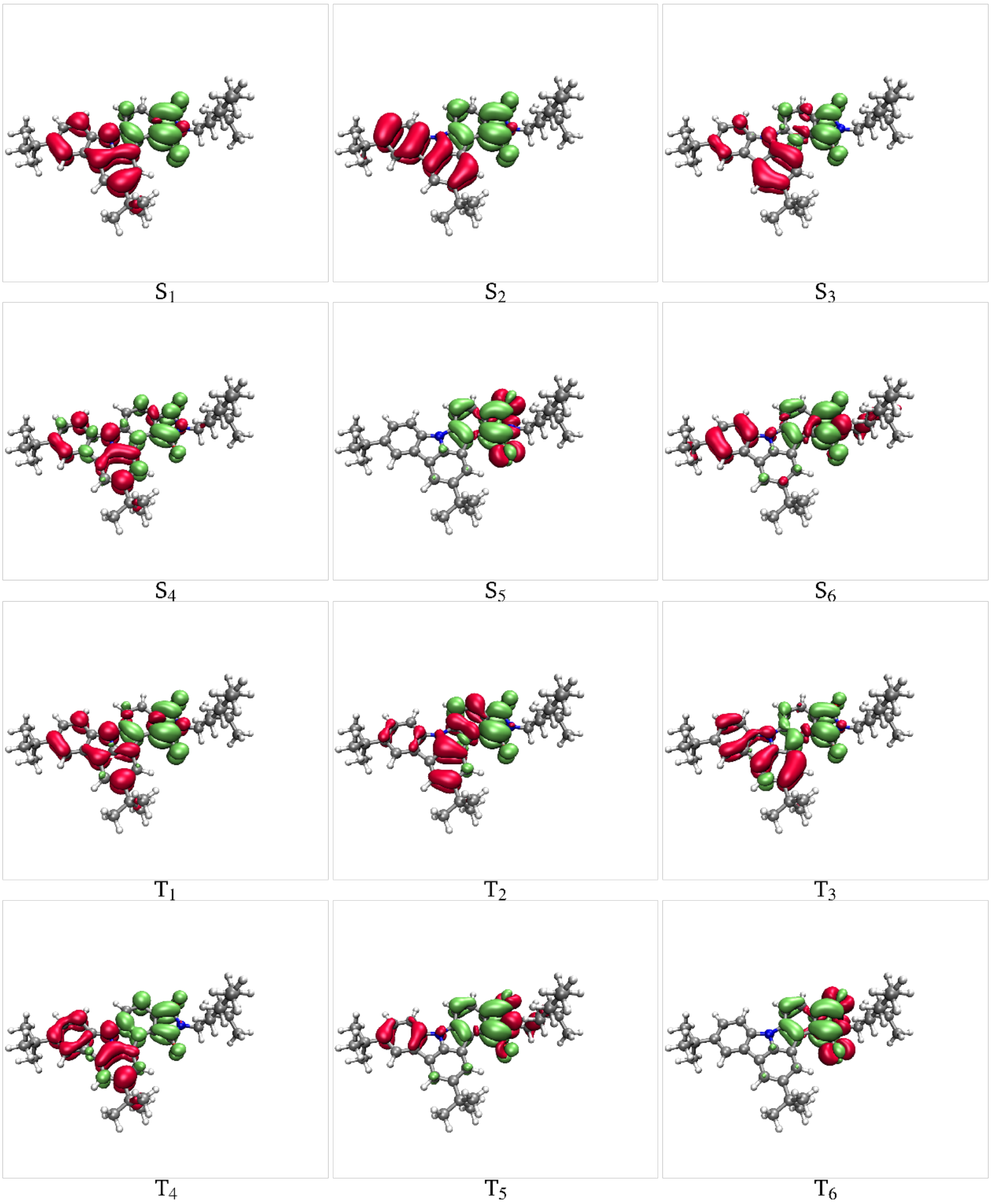

Supplementary Figure 31. Electron structure calculations of *o*-ICz-PI. Difference of electronic density associated to the transitions corresponding to the 6 lowest singlet and 6 lowest triplet states for ***o*-ICz-PI** at the S1 geometry in dichloromethane. Red: Loss of electronic density. Green: Gain of electronic density.

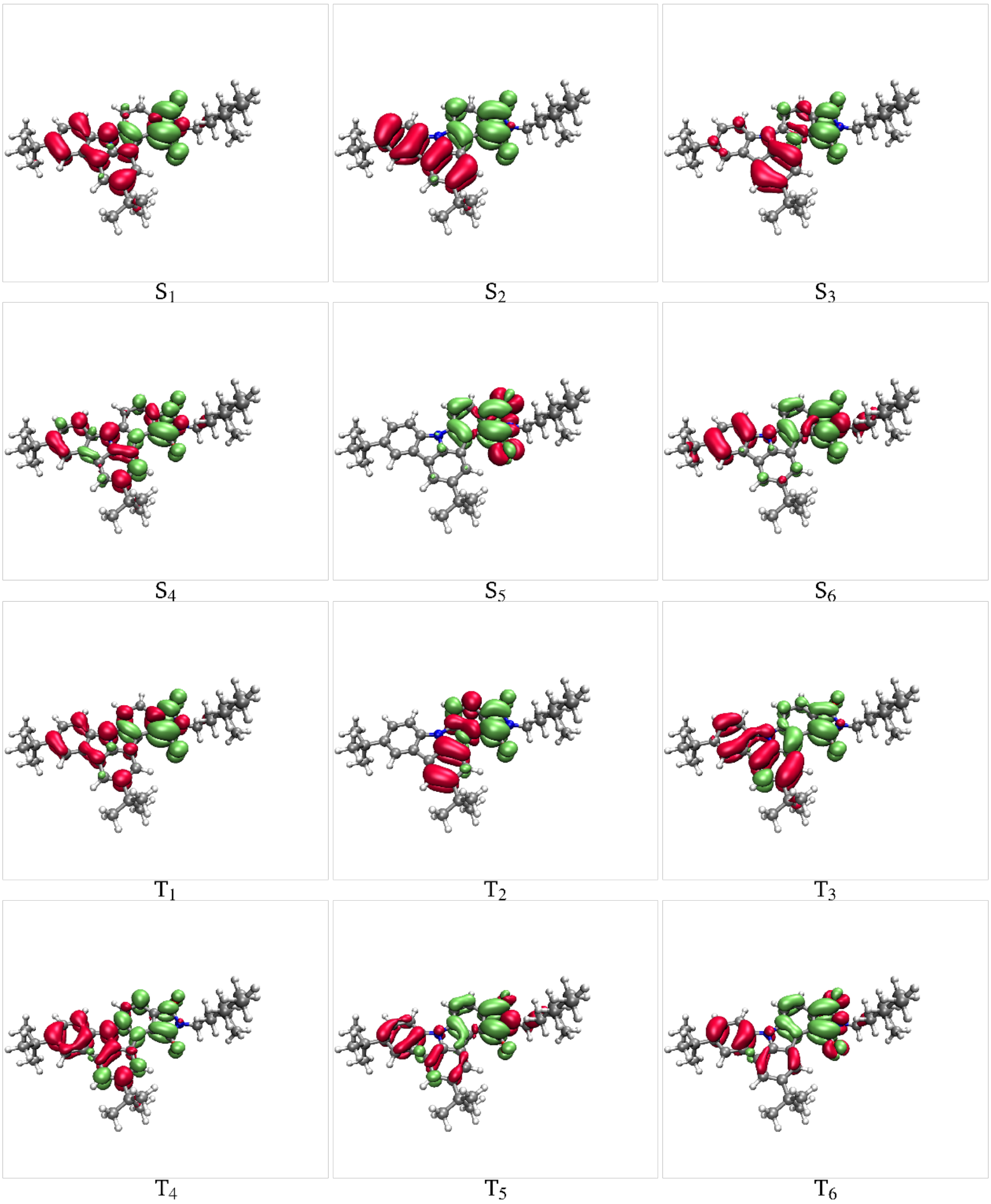

Supplementary Figure 32. Electron structure calculations of *o*-ICz-PI. Difference of electronic density associated to the transitions corresponding to the 6 lowest singlet and 6 lowest triplet states for ***o*-ICz-PI** at the T1 geometry in dichloromethane. Red: Loss of electronic density. Green: Gain of electronic density.

5.3. Cz-PI

5.3.1 Electronic structure – Gas Phase

State	f	Δ E / eV	State	f	Δ E / eV	State	f	Δ E / eV
S_0	—	0.00	S_0	—	0.37	S_0	—	0.24
S_1	0.136	2.95	S_1	0.000	2.47	S_1	0.107	2.68
S_2	0.000	3.38	S_2	0.002	3.10	S_2	0.000	3.22
S_3	0.112	3.74	S_3	0.001	3.72	S_3	0.025	3.86
S_4	0.001	3.97	S_4	0.000	3.93	S_4	0.072	3.87
S_5	0.053	4.09	S_5	0.007	3.96	S_5	0.010	4.05
S_6	0.001	4.24	S_6	0.005	4.14	S_6	0.008	4.11
S_7	0.007	4.34	S_7	0.001	4.37	S_7	0.055	4.29
S_8	0.002	4.37	S_8	0.000	4.43	S_8	0.000	4.37
S_9	0.000	4.52	S_9	0.064	4.44	S_9	0.002	4.49
S_{10}	0.138	4.63	S_{10}	0.002	4.93	S_{10}	0.063	4.84
T_1	—	2.65	T_1	—	2.46	T_1	—	2.40
T_2	—	3.31	T_2	—	3.09	T_2	—	3.19
T_3	—	3.36	T_3	—	3.36	T_3	—	3.38
T_4	—	3.44	T_4	—	3.53	T_4	—	3.47
T_5	—	3.47	T_5	—	3.61	T_5	—	3.53
T_6	—	3.56	T_6	—	3.69	T_6	—	3.63
T_7	—	3.67	T_7	—	3.77	T_7	—	3.73
T_8	—	3.94	T_8	—	3.88	T_8	—	3.75
T_9	—	4.01	T_9	—	3.94	T_9	—	3.96
T_{10}	—	4.10	T_{10}	—	4.02	T_{10}	—	4.02

Supplementary Table 14. Electronic structure of **Cz-PI** in gas phase at the ground state (left), S1 (centre) and T1 (right) minimum energy geometries. The associated differences of electronic density are reported in Supplementary Fig. 33, 34 and 35.

GS

SOC / cm^{-1}	S1	S2	T1	T2	T3	T4	T5
T1	0.362	0.242	---	---	---	---	---
T2	0.248	0.208	1.646	---	---	---	---
T3	0.439	0.052	1.935	0.439	---	---	---
T4	0.278	0.228	1.641	0.278	0.228	---	---
T5	0.247	0.167	4.151	0.247	0.167	0.327	---

S1

SOC / cm-1	S1	S2	T1	T2	T3	T4	T5
T1	0.024	0.134	---	---	---	---	---
T2	0.181	0.022	0.873	---	---	---	---
T3	0.923	0.459	2.393	0.923	---	---	---
T4	1.055	0.478	55.474	1.055	0.478	---	---
T5	0.203	0.066	0.974	0.203	0.066	0.140	---

T1

SOC / cm-1	S1	S2	T1	T2	T3	T4	T5
T1	0.333	0.259	---	---	---	---	---
T2	0.327	0.058	2.073	---	---	---	---
T3	0.735	0.336	0.723	0.735	---	---	---
T4	3.328	0.182	54.916	3.328	0.182	---	---
T5	0.323	0.096	2.226	0.323	0.096	0.523	---

Supplementary Table 15. Spin-orbit coupling matrix elements **Cz-PI** in gas phase at the ground state (top), S1 (middle) and T1 (bottom) minimum energy geometries.

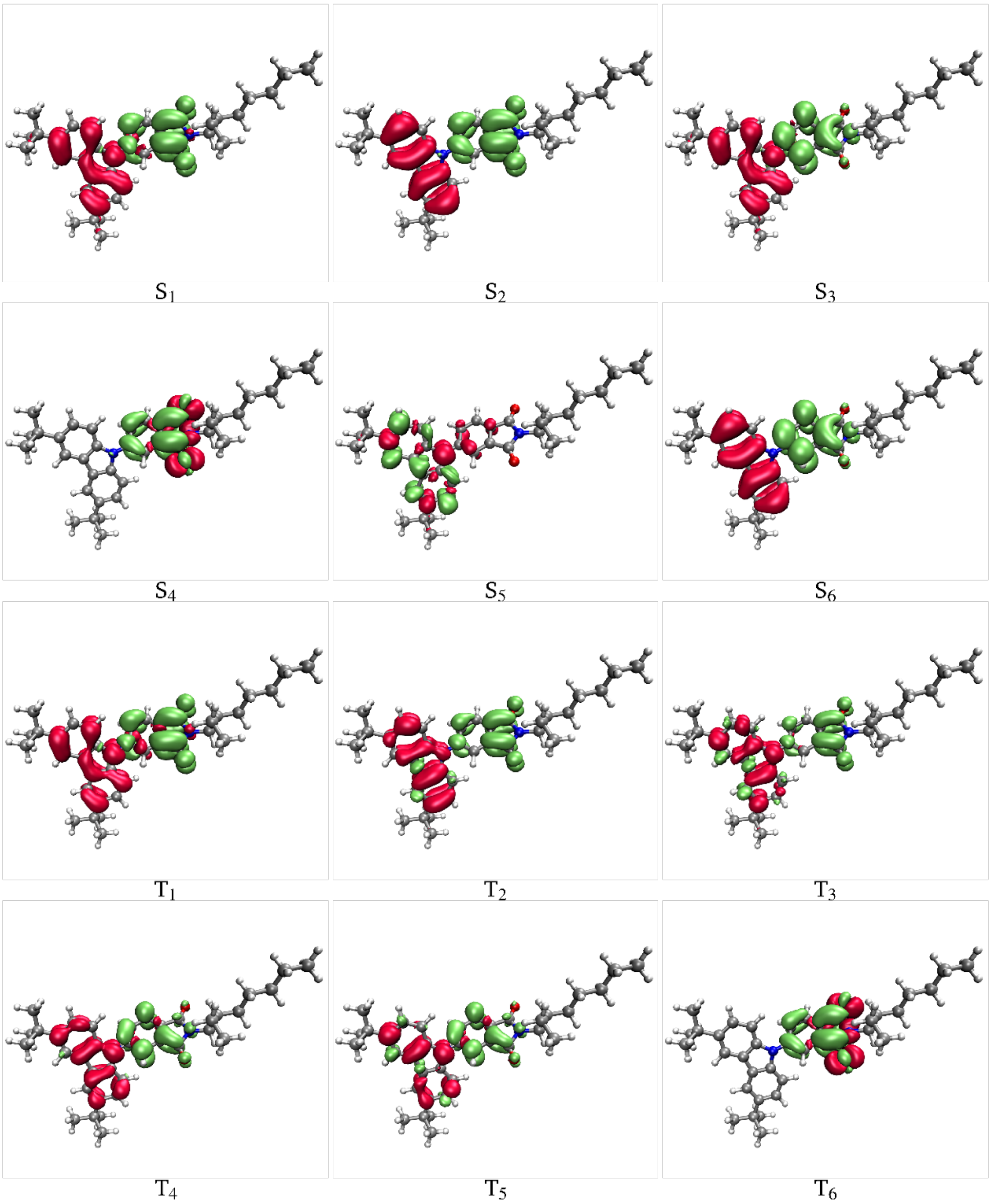

Supplementary Figure 33. Electron structure calculations of Cz-PI. Difference of electronic density associated to the transitions corresponding to the 6 lowest singlet and 6 lowest triplet states for **Cz-PI** at the GS geometry in gas phase. Red: Loss of electronic density. Green: Gain of electronic density.

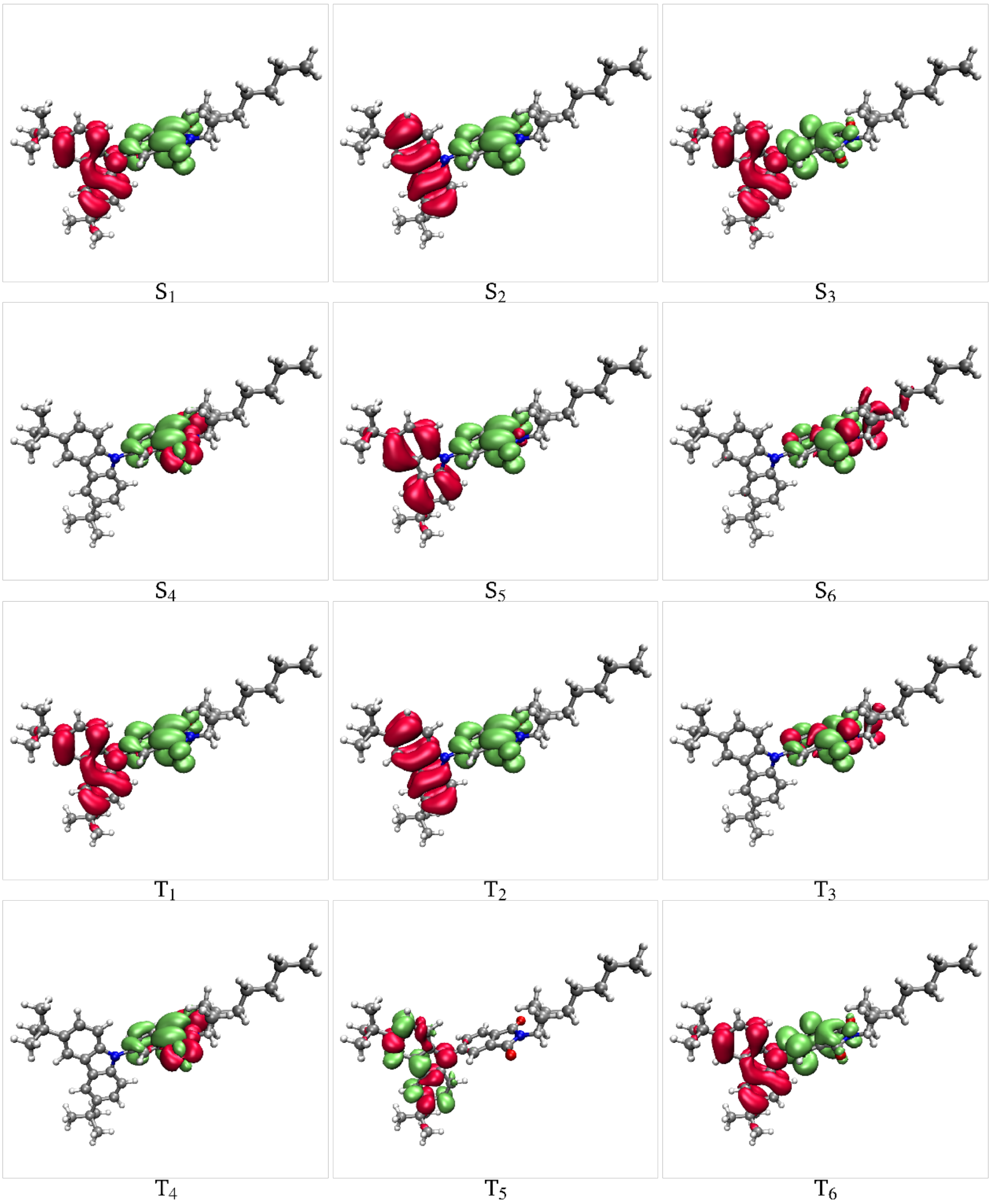

Supplementary Figure 34. Electron structure calculations of Cz-PI. Difference of electronic density associated to the transitions corresponding to the 6 lowest singlet and 6 lowest triplet states for **Cz-PI** at the S1 geometry in gas phase. Red: Loss of electronic density. Green: Gain of electronic density.

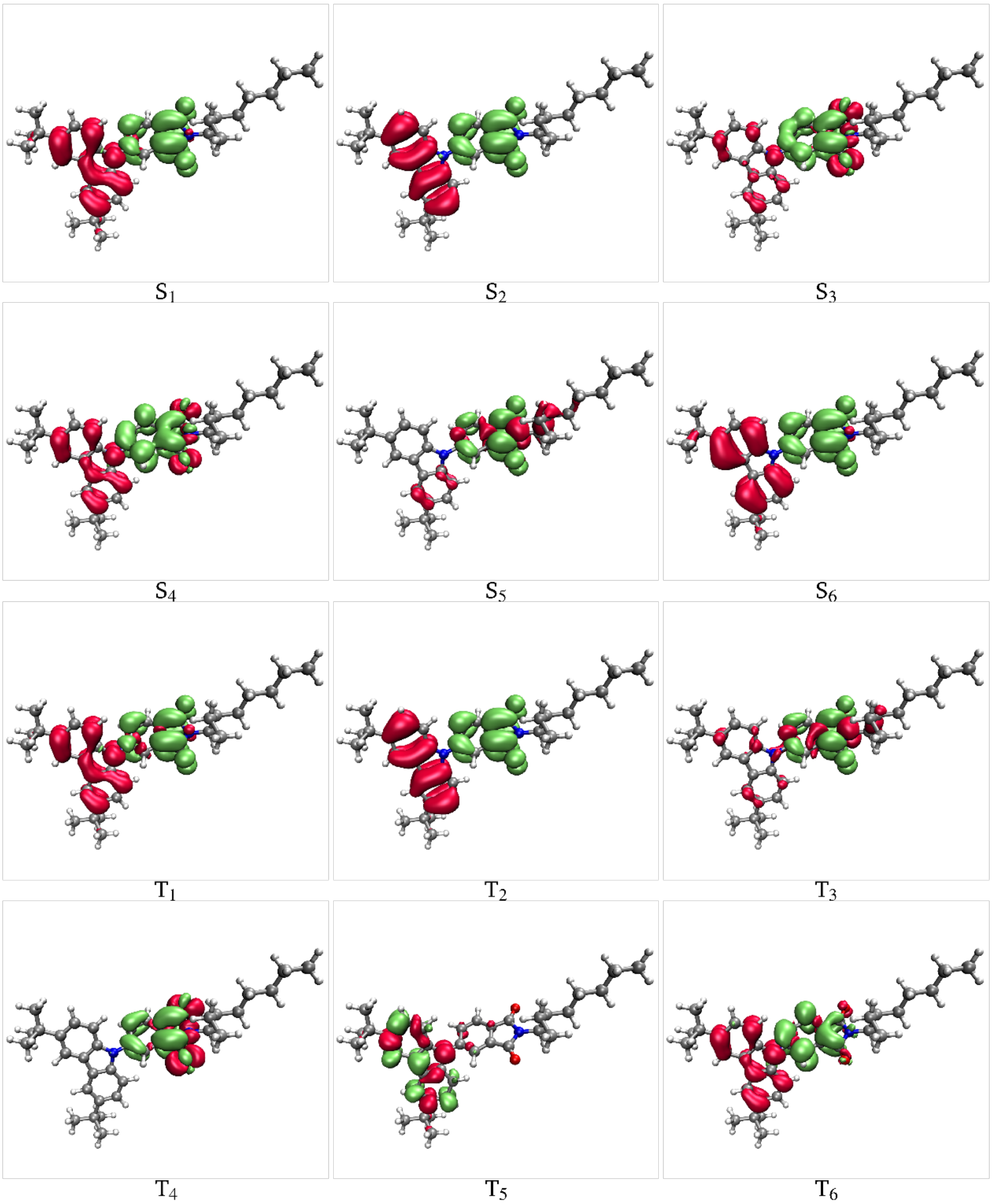

Supplementary Figure 35. Electron structure calculations of Cz-PI. Difference of electronic density associated to the transitions corresponding to the 6 lowest singlet and 6 lowest triplet states for **Cz-PI** at the T1 geometry in gas phase. Red: Loss of electronic density. Green: Gain of electronic density.

5.3.2 Electronic structure – Dichloromethane

State	f	Δ E / eV	State	f	Δ E / eV	State	f	Δ E / eV
S_0	—	0.00	S_0	—	0.36	S_0	—	0.25
S_1	0.157	2.85	S_1	0.003	2.49	S_1	0.211	2.63
S_2	0.000	3.33	S_2	0.003	3.16	S_2	0.001	3.24
S_3	0.125	3.77	S_3	0.003	3.88	S_3	0.015	3.91
S_4	0.075	4.05	S_4	0.001	3.95	S_4	0.097	3.98
S_5	0.001	4.08	S_5	0.002	4.01	S_5	0.120	3.99
S_6	0.007	4.24	S_6	0.017	4.03	S_6	0.021	4.11
S_7	0.005	4.28	S_7	0.108	4.32	S_7	0.083	4.24
S_8	0.002	4.32	S_8	0.000	4.48	S_8	0.000	4.45
S_9	0.250	4.59	S_9	0.020	4.59	S_9	0.003	4.66
S_{10}	0.013	4.60	S_{10}	0.155	4.79	S_{10}	0.275	4.74
T_1	—	2.58	T_1	—	2.47	T_1	—	2.31
T_2	—	3.28	T_2	—	3.14	T_2	—	3.21
T_3	—	3.34	T_3	—	3.26	T_3	—	3.36
T_4	—	3.46	T_4	—	3.59	T_4	—	3.56
T_5	—	3.50	T_5	—	3.63	T_5	—	3.60
T_6	—	3.60	T_6	—	3.78	T_6	—	3.67
T_7	—	3.69	T_7	—	3.84	T_7	—	3.76
T_8	—	3.96	T_8	—	3.89	T_8	—	3.81
T_9	—	3.99	T_9	—	3.89	T_9	—	3.94
T_{10}	—	4.03	T_{10}	—	4.07	T_{10}	—	4.05

Supplementary Table 16. Electronic structure of **Cz-PI** at the GS geometry in dichloromethane at the ground state (left), S1 (centre) and T1 (right) minimum energy geometries. The associated differences of electronic density are reported in Supplementary Fig. 36, 37 and 38.

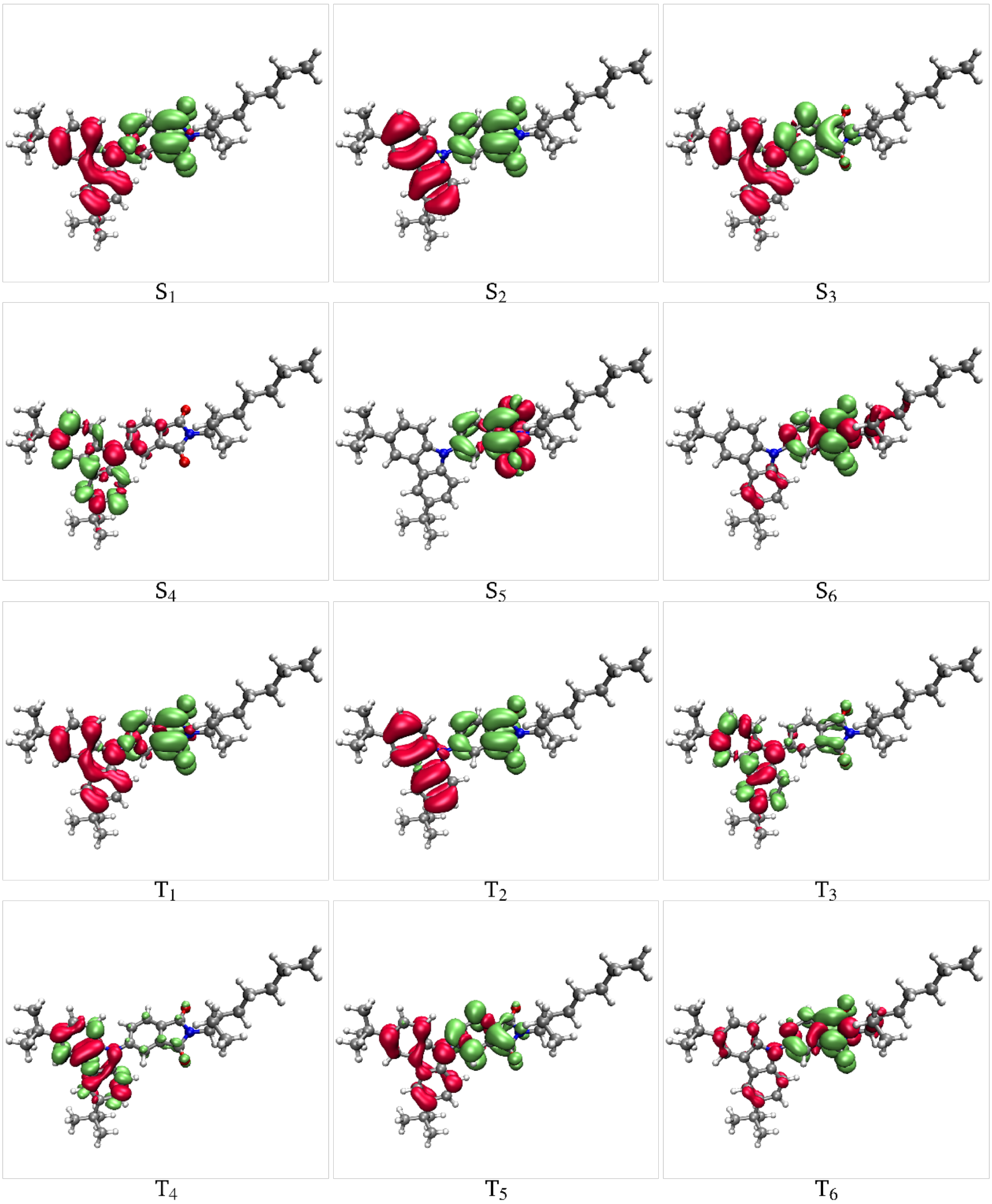

Supplementary Figure 36. Electron structure calculations of Cz-PI. Difference of electronic density associated to the transitions corresponding to the 6 lowest singlet and 6 lowest triplet states for **Cz-PI** at the GS geometry in dichloromethane. Red: Loss of electronic density. Green: Gain of electronic density.

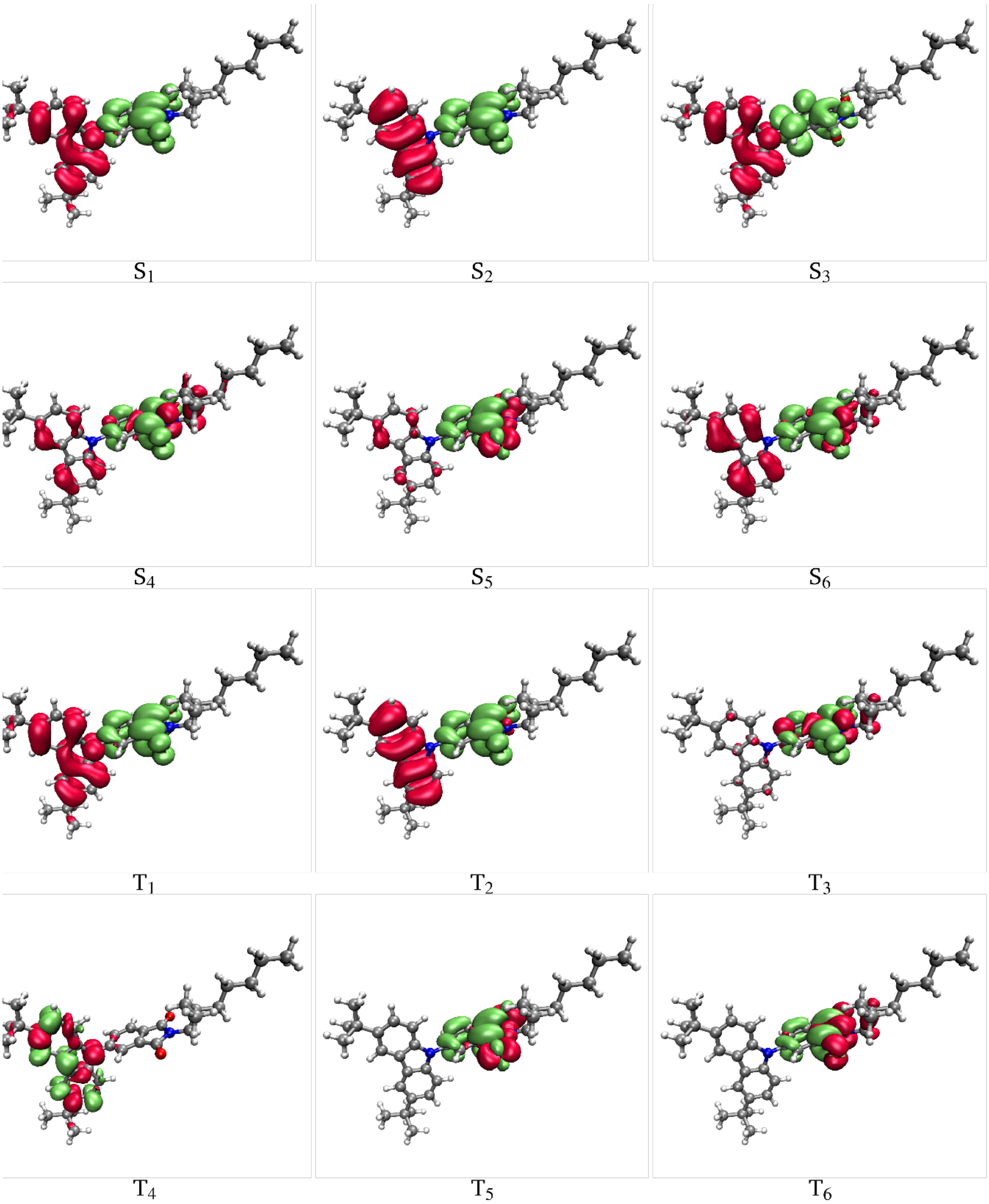

Supplementary Figure 37. Electron structure calculations of Cz-PI. Difference of electronic density associated to the transitions corresponding to the 6 lowest singlet and 6 lowest triplet states for **Cz-PI** at the S1 geometry in dichloromethane. Red: Loss of electronic density. Green: Gain of electronic density.

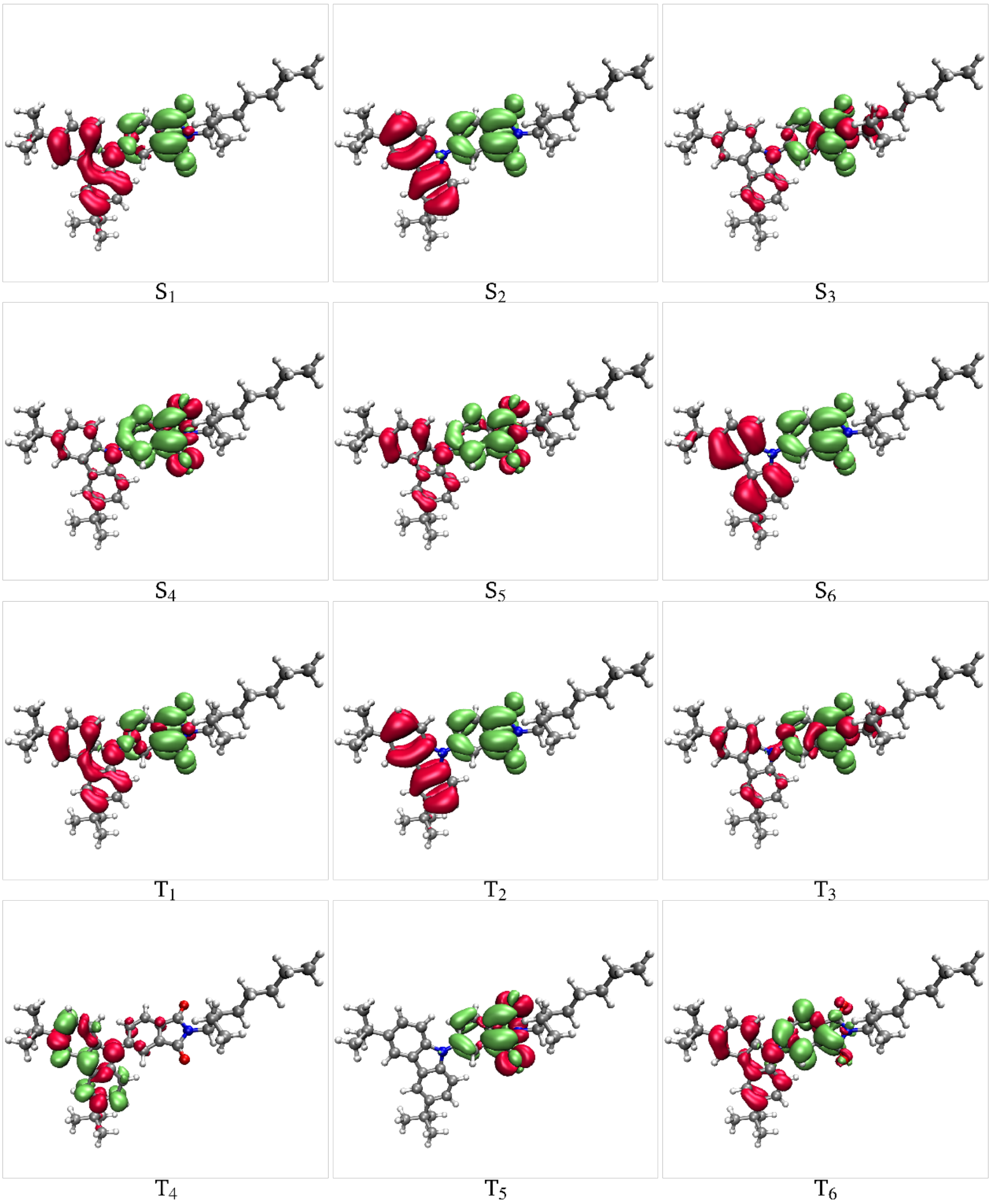

Supplementary Figure 38. Electron structure calculations of Cz-PI. Difference of electronic density associated to the transitions corresponding to the 6 lowest singlet and 6 lowest triplet states for **Cz-PI** at the T1 geometry in dichloromethane. Red: Loss of electronic density. Green: Gain of electronic density.

6. Characterization Spectra.

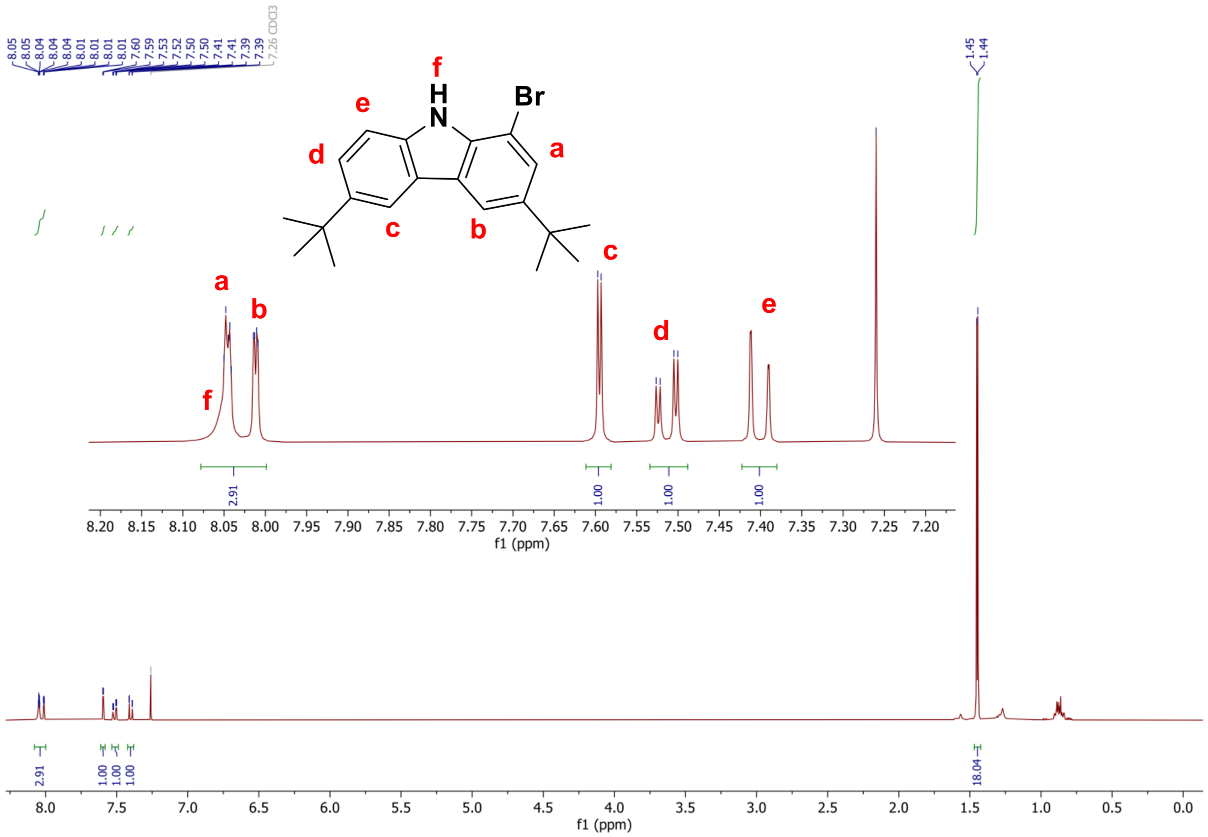

Supplementary Figure 39. ^{1}H NMR spectrum of **2** in $CDCl_3$ at RT.

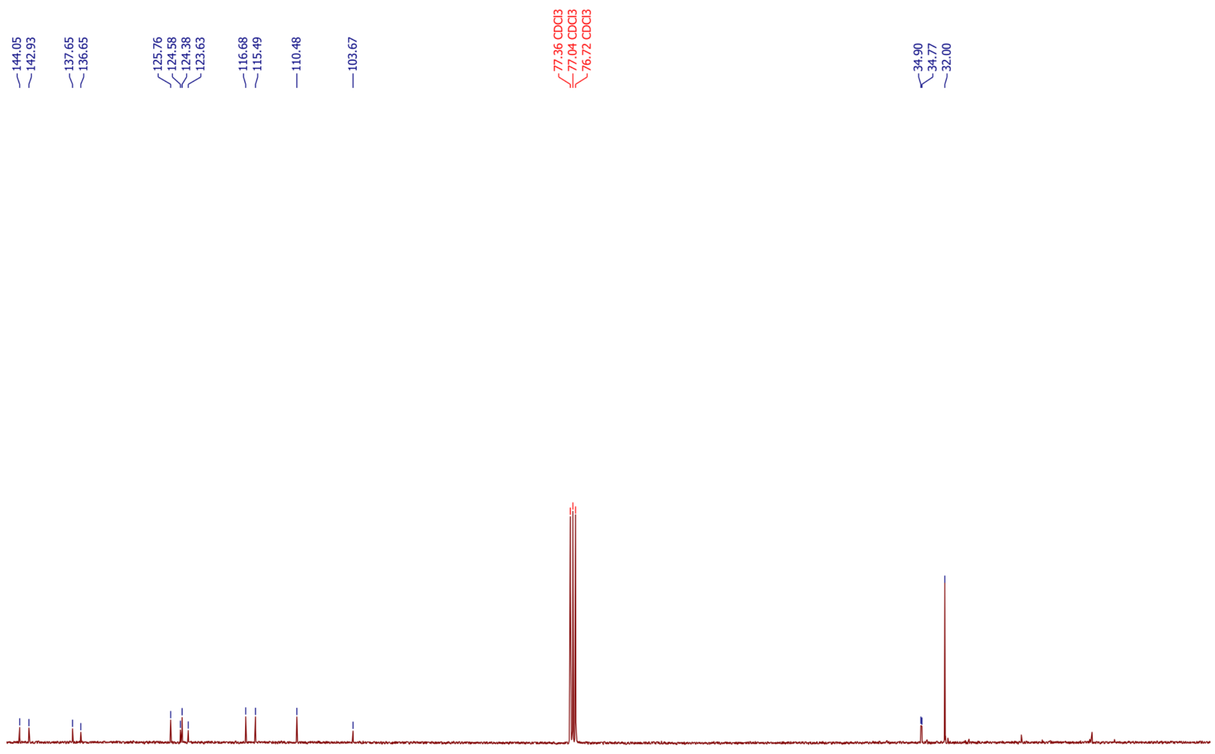

Supplementary Figure 40. ^{13}C NMR spectrum of **2** in $CDCl_3$ at RT.

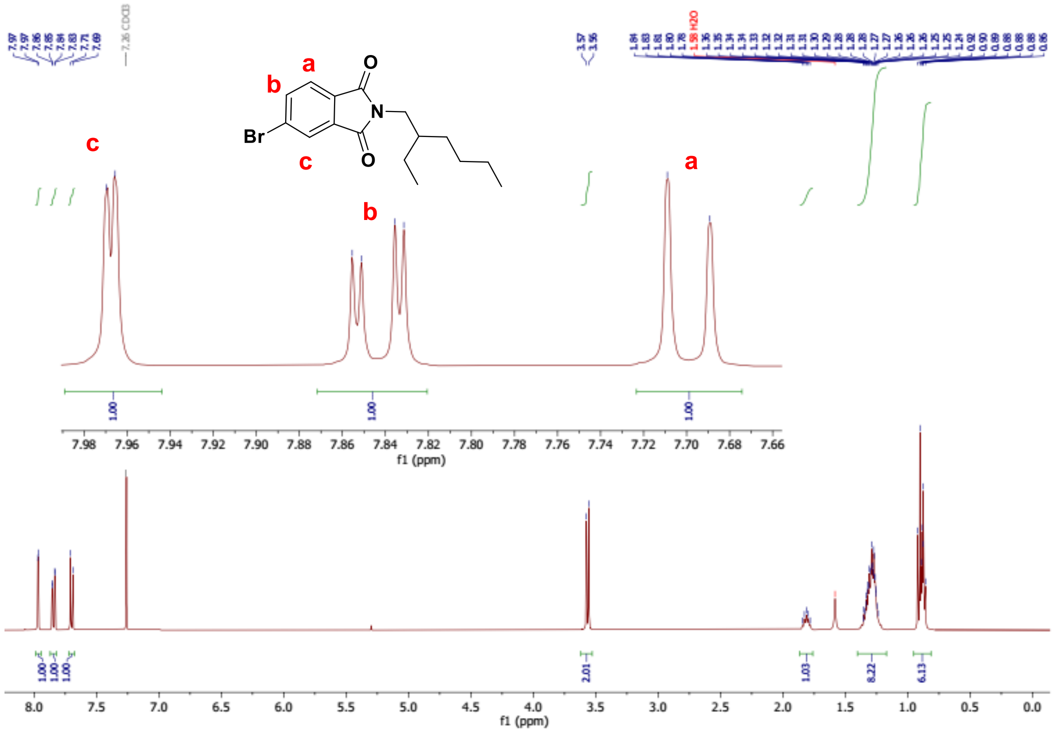

Supplementary Figure 41. ^{1}H NMR spectrum of **5** in $CDCl_3$ at RT.

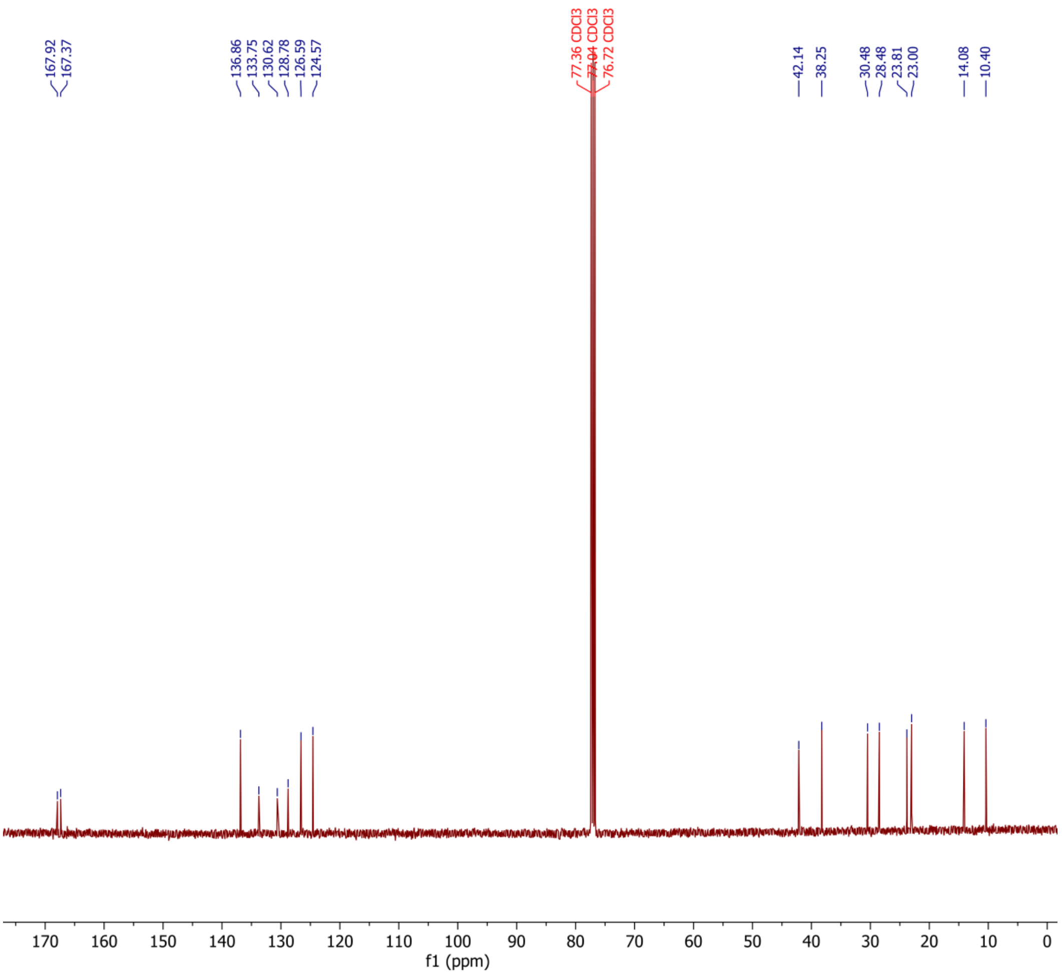

Supplementary Figure 42. ^{13}C NMR spectrum of **5** in $CDCl_3$ at RT.

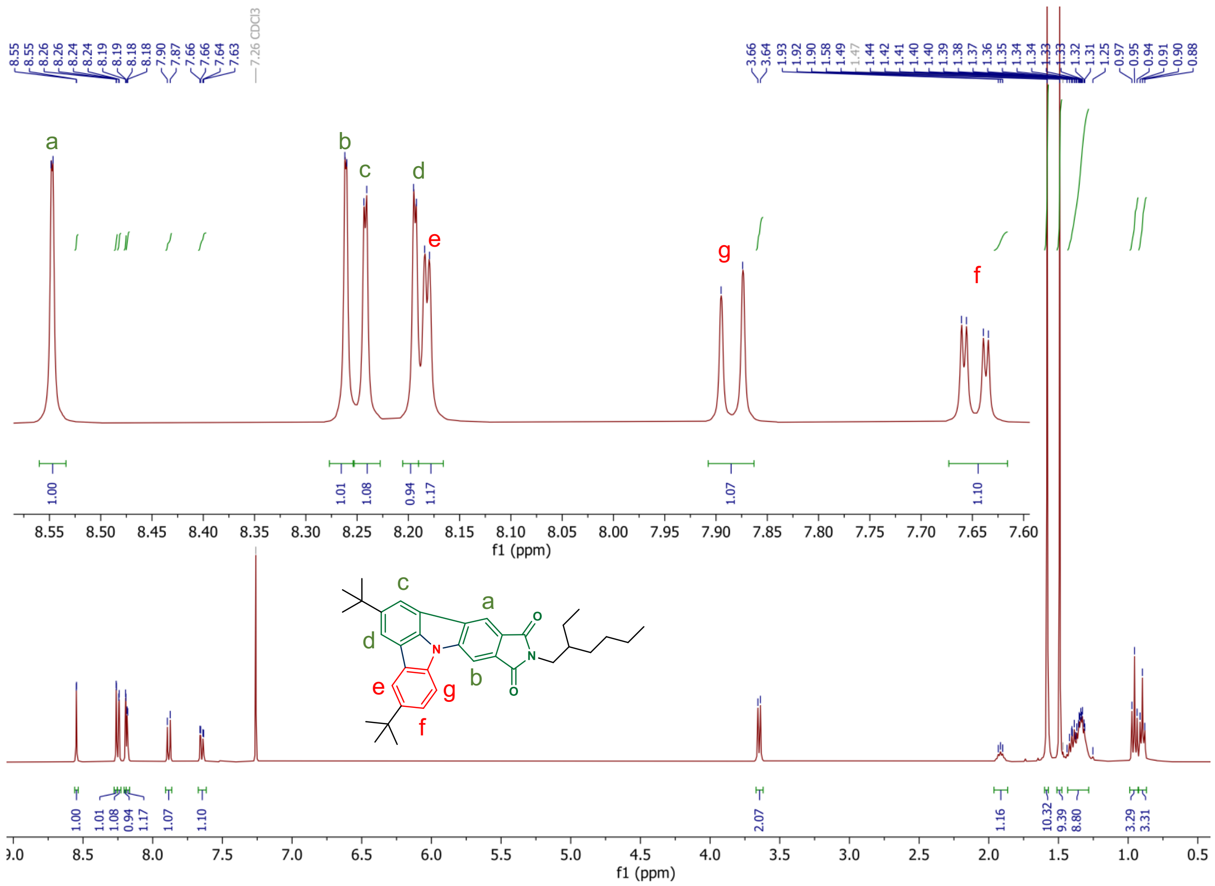

Supplementary Figure 43. ^{1}H NMR spectrum of ***p*-ICz-PI** in $CDCl_3$ at RT.

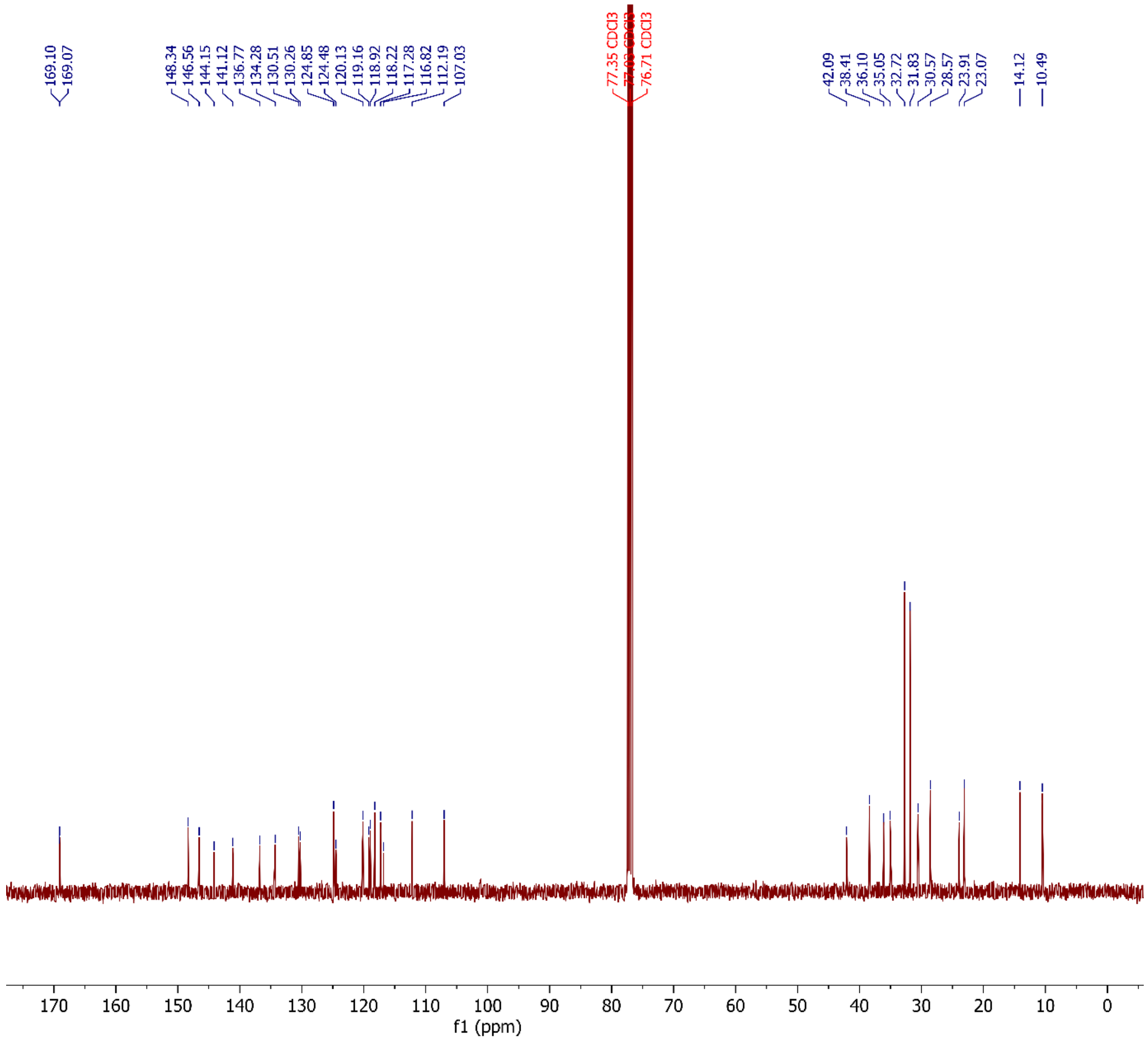

Supplementary Figure 44. ^{13}C NMR spectrum of ***p*-ICz-PI** in $CDCl_3$ at RT.

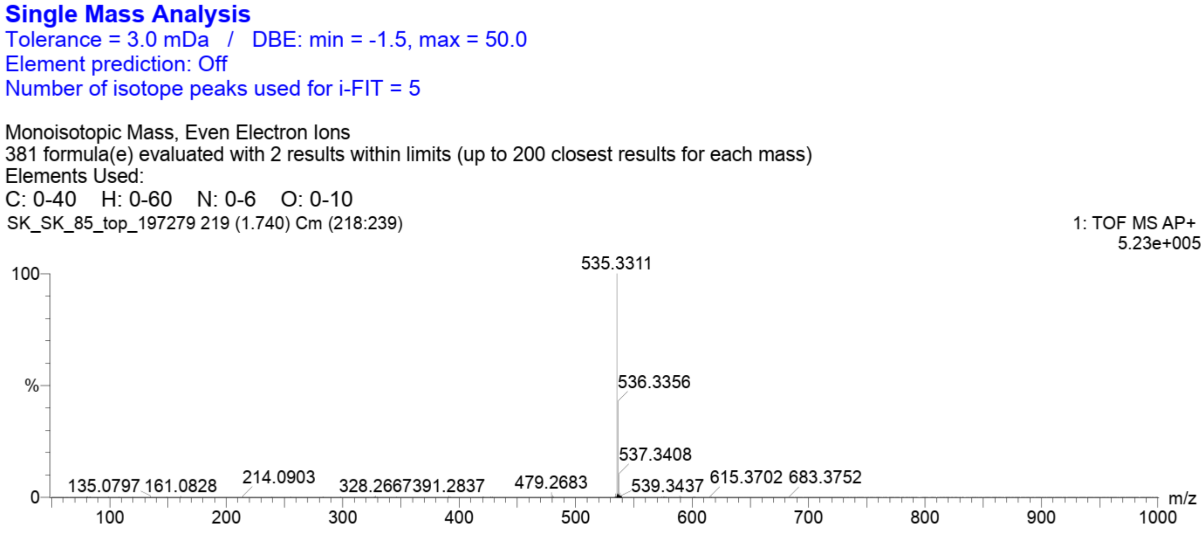

Supplementary Figure 45. HRMS-APCI spectrum of ***p*-ICz-PI**.

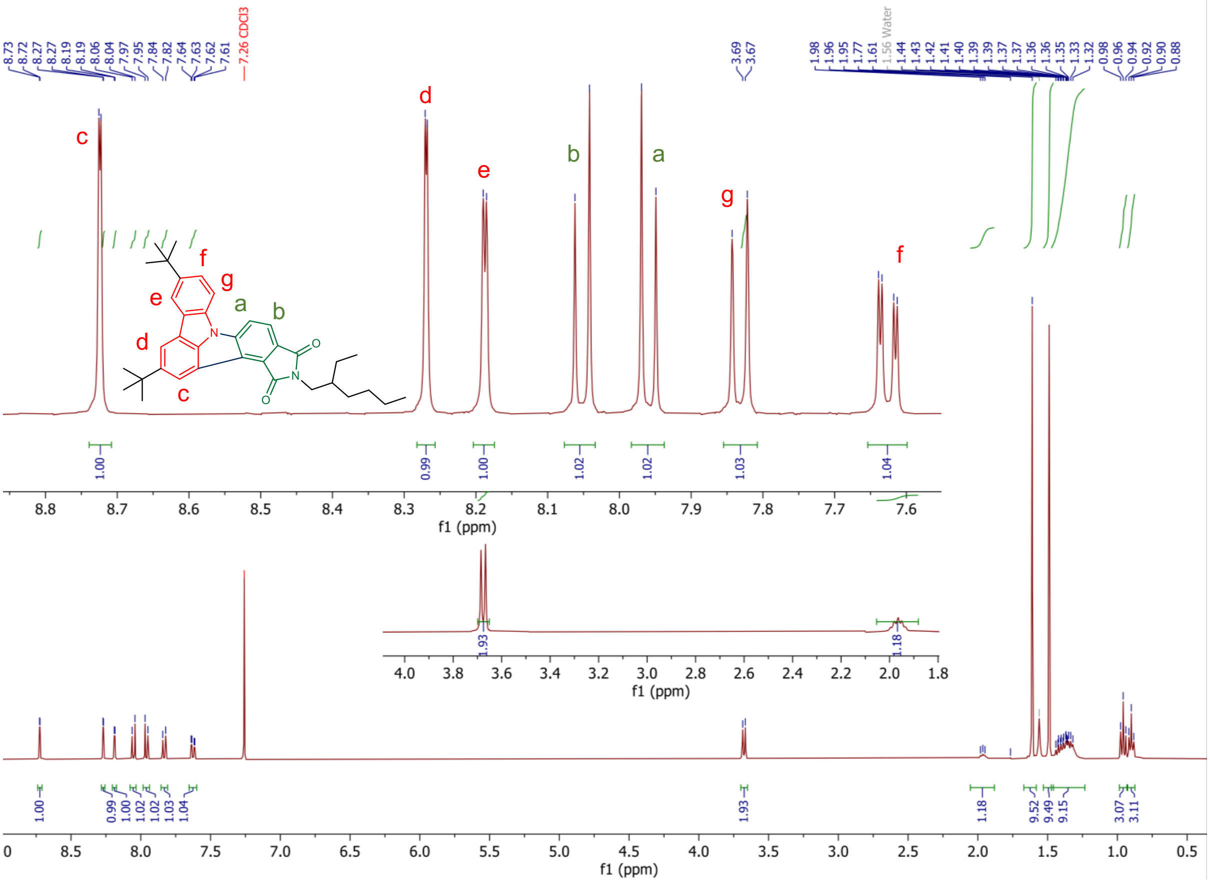

Supplementary Figure 46. ^{1}H NMR spectrum of ***o*-ICz-PI** in $CDCl_3$ at RT.

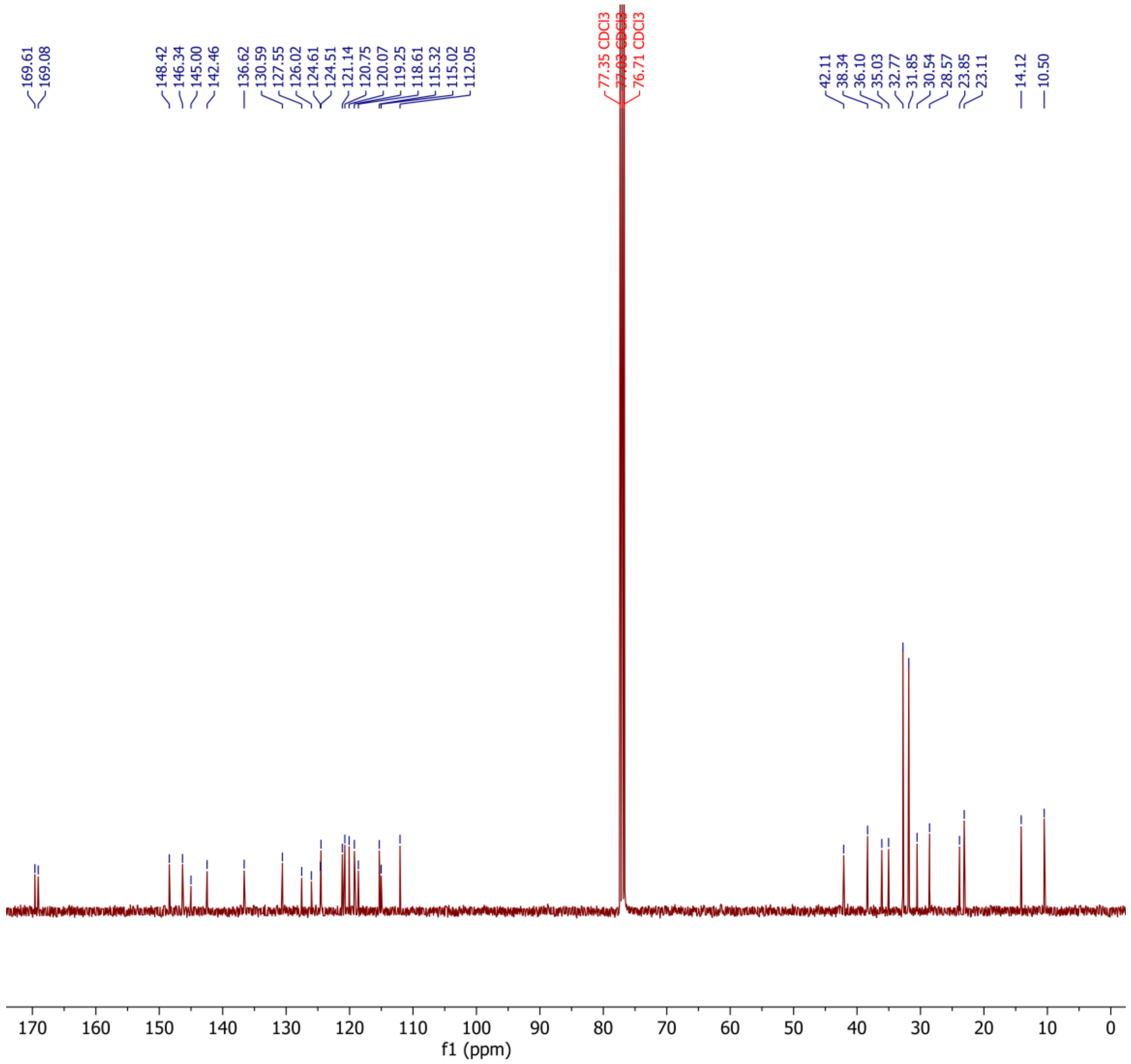

Supplementary Figure 47. ^{13}C NMR spectrum of ***o*-ICz-PI** in $CDCl_3$ at RT.

Single Mass Analysis
Tolerance = 3.0 mDa / DBE: min = -1.5, max = 50.0
Element prediction: Off
Number of isotope peaks used for i-FIT = 5

Monoisotopic Mass, Even Electron Ions
767 formula(e) evaluated with 4 results within limits (up to 200 closest results for each mass)
Elements Used:
C: 0-40 H: 0-60 N: 0-6 O: 0-10 10B: 0-1

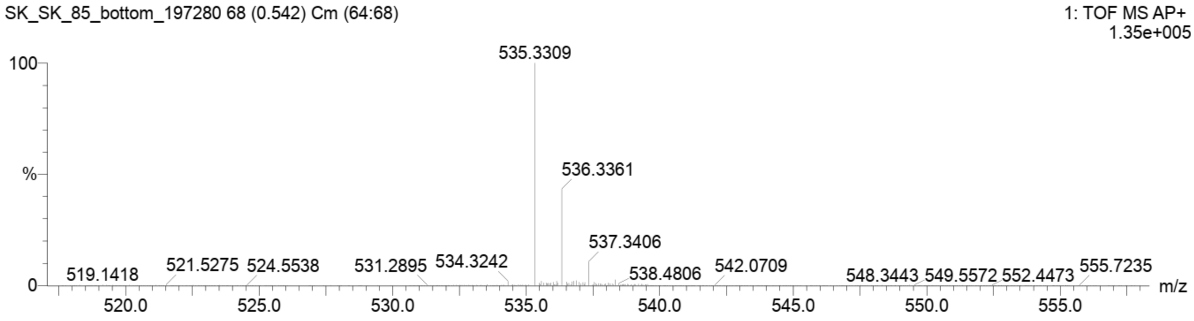

Supplementary Figure 48. HRMS-APCI spectrum of ***o*-ICz-PI**.

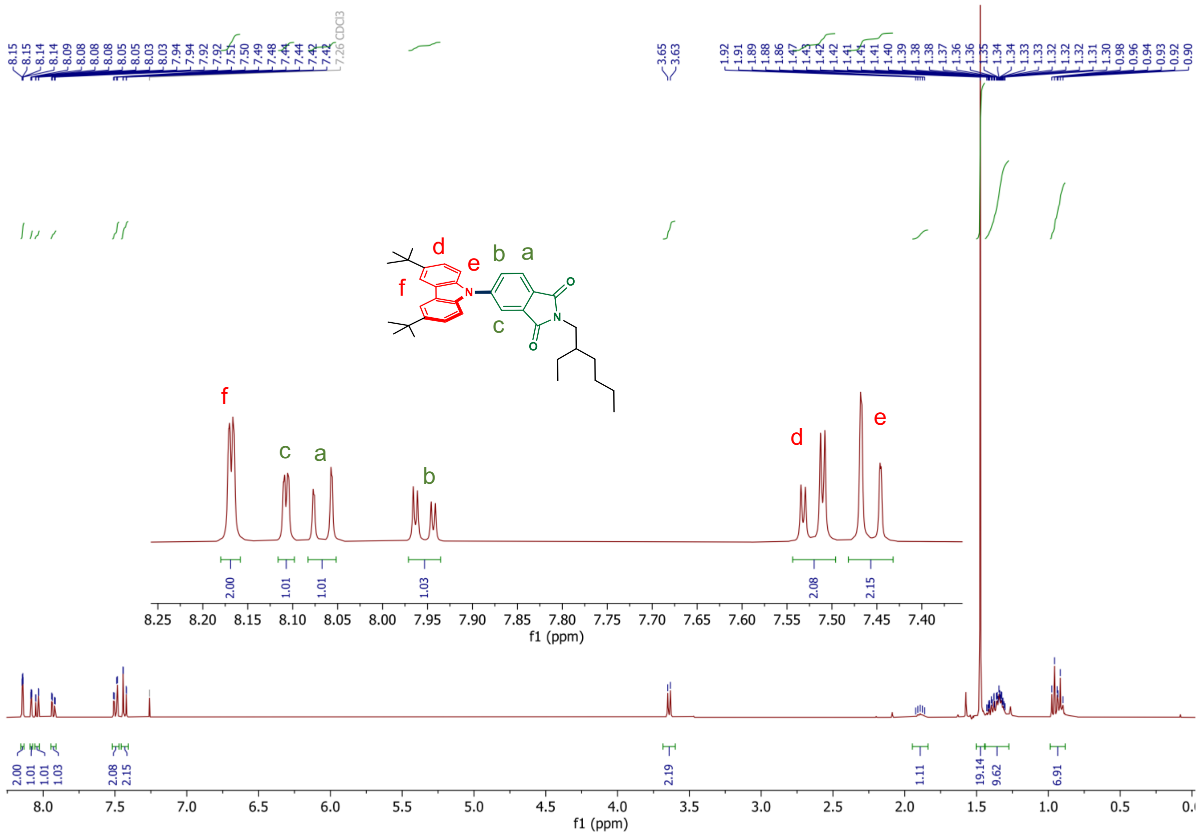

Supplementary Figure 49. ^{1}H NMR spectrum of **Cz-PI** in $CDCl_3$ at RT.

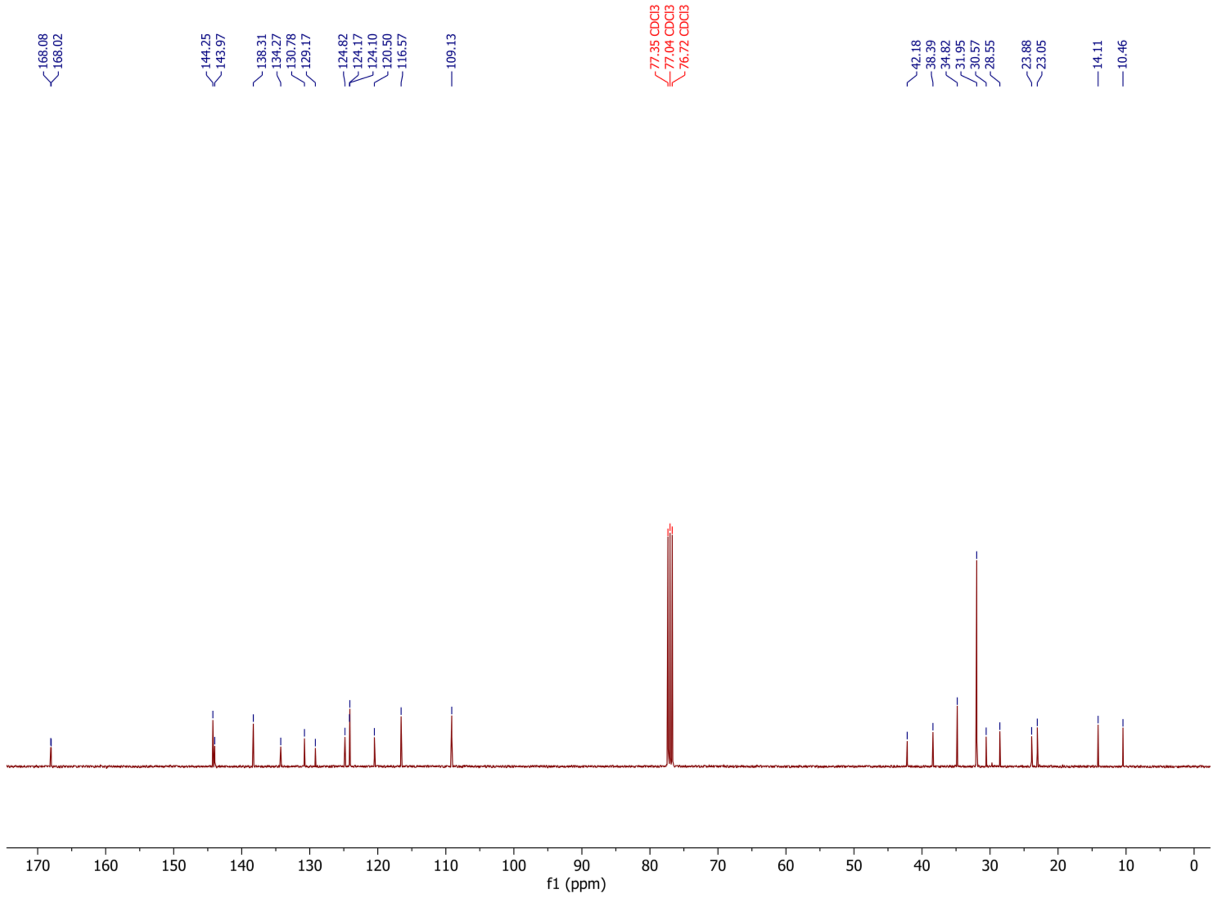

Supplementary Figure 51. ^{13}C NMR spectrum of **Cz-PI** in $CDCl_3$ at RT.

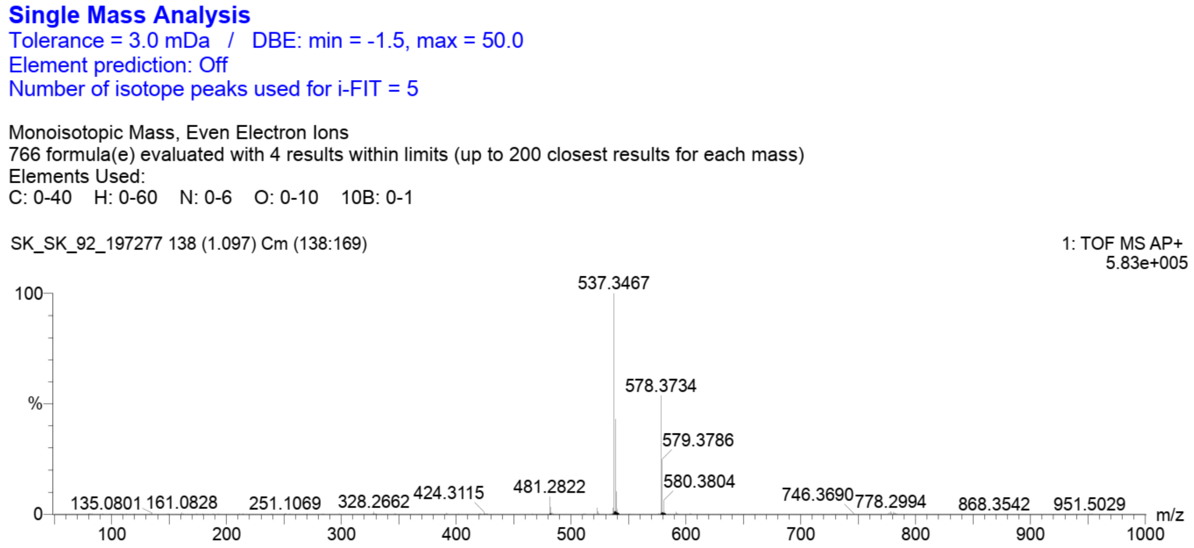

Supplementary Figure 52. HRMS-APCI spectrum of **Cz-PI**.

7. References.

1. Fulmer, G. R. et al. NMR Chemical Shifts of Trace Impurities: Common Laboratory Solvents, Organics, and Gases in Deuterated Solvents Relevant to the Organometallic Chemist. *Organometallics*, **29**, 2176–2179 (2010).

2. Araneda, J. F., Piers, W. E., Heyne, B., Parvez, M., & McDonald, R. High Stokes-Shift Anilido-Pyridine Boron Difluoride Dyes. *Angew. Chem. Int. Ed.* **50**, 12214-12217 (2011).

3. Douglas, J. D. et al. Solution-Processed, Molecular Photovoltaics that Exploit Hole Transfer from Non-Fullerene, n-Type Materials. *Adv. Mater.* **26**, 4313-4319 (2014).

4. Feng, Q. et al. A 9-fluorenyl substitution strategy for aromatic-imide-based TADF emitters towards efficient and stable sky-blue OLEDs with nearly 30% external quantum efficiency. *Mater. Adv.* **2**, 4000-4008 (2021).

5. Penfold, T. J. et al. The theory of thermally activated delayed fluorescence for organic light emitting diodes. *Chem. Commun.* **54**, 3926-3935 (2018).
