# Supplementary material for: Rigid and planar π-conjugated molecules leading to long-lived intramolecular charge-transfer states exhibiting thermally activated delayed fluorescence
Source: Nat Commun. 2024 Nov 7;15:9611. doi: 10.1038/s41467-024-53740-1 (PMC11544105; doi:10.1038/s41467-024-53740-1)
Supplement: Supplementary file 2 — Transparent Peer Review file [file 41467_2024_53740_MOESM2_ESM.pdf]

nature portfolio

Peer Review File

Rigid and planar π-conjugated molecules leading to long-lived intramolecular charge-transfer states exhibiting thermally activated delayed fluorescence

Corresponding Author: Professor Andrew Monkman

This manuscript has been previously reviewed at another journal. This document only contains reviewer comments, rebuttal and decision letters for versions considered at Nature Communications.

Version 0:

Reviewer comments:

Reviewer #1

(Remarks to the Author)
This work can be published.

Reviewer #2

(Remarks to the Author)
The manuscript has been significantly revised in response to my original concerns and now appears ready for publication.

Open Access This Peer Review File is licensed under a Creative Commons Attribution 4.0 International License, which permits use, sharing, adaptation, distribution and reproduction in any medium or format, as long as you give appropriate

credit to the original author(s) and the source, provide a link to the Creative Commons license, and indicate if changes were made.
In cases where reviewers are anonymous, credit should be given to 'Anonymous Referee' and the source.
The images or other third party material in this Peer Review File are included in the article's Creative Commons license, unless indicated otherwise in a credit line to the material. If material is not included in the article's Creative Commons license and your intended use is not permitted by statutory regulation or exceeds the permitted use, you will need to obtain permission directly from the copyright holder.
To view a copy of this license, visit https://creativecommons.org/licenses/by/4.0/
